# Sulfinylnitrenes via Chemoselective S–N Bond Cleavage: Design, Synthesis, and Applications

**DOI:** 10.1021/acs.orglett.6c01673

**Published:** 2026-05-14

**Authors:** Prakash Kafle, Rishav Mukherjee, Shuhei Yasuda, Indrajeet Sharma

**Affiliations:** Department of Chemistry and Biochemistry, 6187 University of Oklahoma, 101 Stephenson Parkway, Norman, Oklahoma 73019-5251, United States

## Abstract

Herein, we report the development of bench-stable, scalable sulfinylnitrene precursors that enable the generation of reactive sulfinylnitrene intermediates via chemoselective S–N bond cleavage under thermal or photolytic conditions, without additives or pyrophoric reagents. These intermediates react with nucleophiles (N or O) to afford sulfonimidamides, a privileged pharmacophore, or sulfonimidates, a versatile intermediate in deoxygenating reactions. This operationally simple transformation provides a general strategy for S–N bond formation and enables late-stage functionalization of amines.

## Graphical abstract

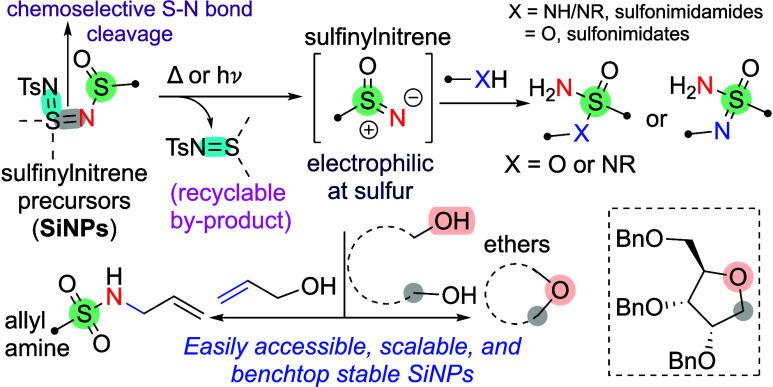

In recent years, sulfinylnitrenes have emerged as important intermediates, providing a versatile platform that broadens the scope of organosulfur chemistry accessible to synthetic chemists.^1^ Organosulfur chemistry is crucial in modern synthetic chemistry because of sulfur’s ability to promote various bond-forming processes and functional group interconversions.^2–15^ The versatility of sulfur-based intermediates arises from sulfur’s distinctive character, which allows effective stabilization of adjacent positive and negative charges.^2–4,9^ This property underpins several fundamental reactivity paradigms, most notably sulfur ylide chemistry, and continues to drive the discovery of new sulfur-based reactive intermediates, including nitrenes.^16–18^ Nitrenes have been widely utilized as versatile reactive intermediates, either as metal-bound nitrenes or generated under mild photoinduced conditions.^19–24^

Sulfur-based nitrenes are classified into sulfonyl-, sulfenyl-, and sulfinyl-nitrenes, each exhibiting different electronic structures and reactivity patterns.^2^ Sulfonylnitrenes^7^ and sulfenylnitrenes^4^ are well-known to be electrophilic at the nitrogen center and undergo nucleophilic attack. In contrast, sulfinylnitrenes exhibit a reversal in reactivity, with the electrophilic site shifting to the sulfur atom, leading to a fundamentally different reactivity pattern (Scheme 1A).^25,26^ This reversal in polarity provides selective methods for creating C–N, S–N, and O–N bonds, making them especially useful for late-stage functionalization.^1^ Additionally, sulfinylnitrenes are ground state singlet nitrenes with S–N bond length ∼1.47 Å.^1,25^

**Scheme 1:**
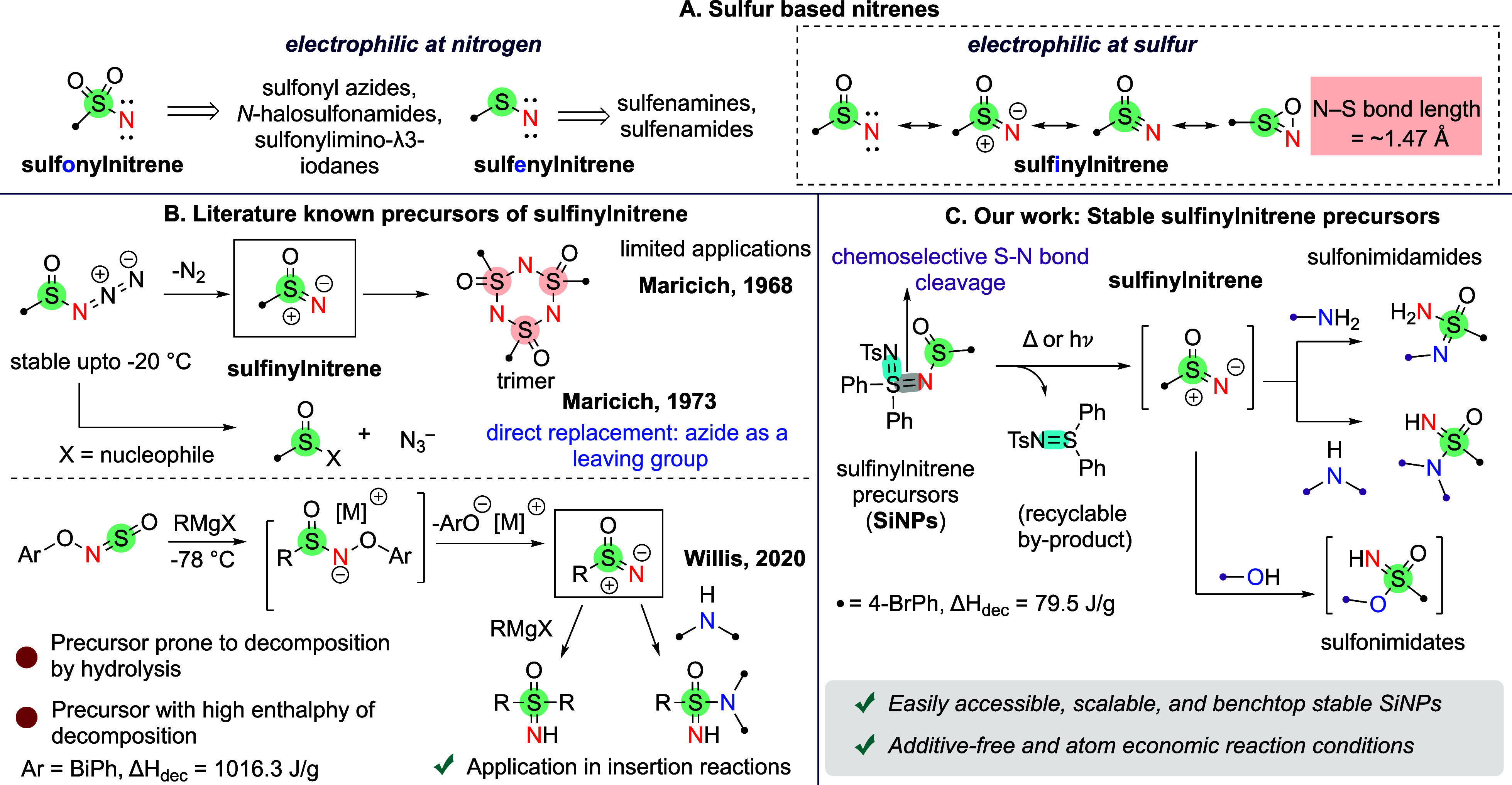
Background and Our Approach to Accessing Sulfinylnitrenes

Maricich’s pioneering work since 1968 has established the formation of sulfinylnitrenes from sulfinyl azides (Scheme 1B).^26–29^ Despite this early progress, the practical usefulness of these precursors has remained limited because sulfinyl azides are inherently unstable and often decompose even under mild conditions.^25,30^ Furthermore, the azide group can act as a competing leaving group in nucleophilic reactions.^29^ Recently, Willis and co-workers demonstrated the synthetic potential of sulfinylnitrenes in the preparation of sulfoximines and sulfonimidamides.^1^ However, this methodology relies on hydrolysis-prone, potentially explosive precursors (enthalpy of decomposition = 1016.3 J/g) and requires pyrophoric reagents.

Sulfonimidamides are common motifs in bioactive natural products and agrochemicals.^31^ The synthesis of sulfonimidamides has traditionally involved sulfenamide and sulfinamide intermediates; however, these methods often require oxidative conditions.^32–39^ Recently, advances in sulfur­(VI) halide exchange chemistry have shown that sulfonimidonyl halides (Cl, F) are versatile electrophiles for reactions with a broad range of nucleophiles.^40–47^ Despite their usefulness, these species are susceptible to hydrolysis and decomposition.^48,49^

To address these challenges, we have developed a bench-stable, scalable sulfinylnitrene precursor that generates highly reactive sulfinylnitrene intermediates via chemoselective S–N cleavage, without the need for additives (Scheme 1C). These intermediates react efficiently with nucleophiles such as amines and alcohols to produce sulfonimidamides, a key pharmacophore in modern medicinal chemistry,^31,50^ and sulfonimidates, a versatile synthetic intermediate,^34,51^ respectively. Based on our previous studies on nitrene generation^2,4,5,52^ we pursued the development of a stable sulfinylnitrene precursor. We began synthesizing sulfinylnitrene precursors (SiNPs) by reacting sulfoximine or *N*-tosylsulfodiimide with sulfinylchloride (Scheme 2A).^53^ Additionally, we synthesized the sulfinylnitrene precursor from a dihydronaphthalene amine, which has the potential to release nitrene via thermal rearomatization.

**Scheme 2:**
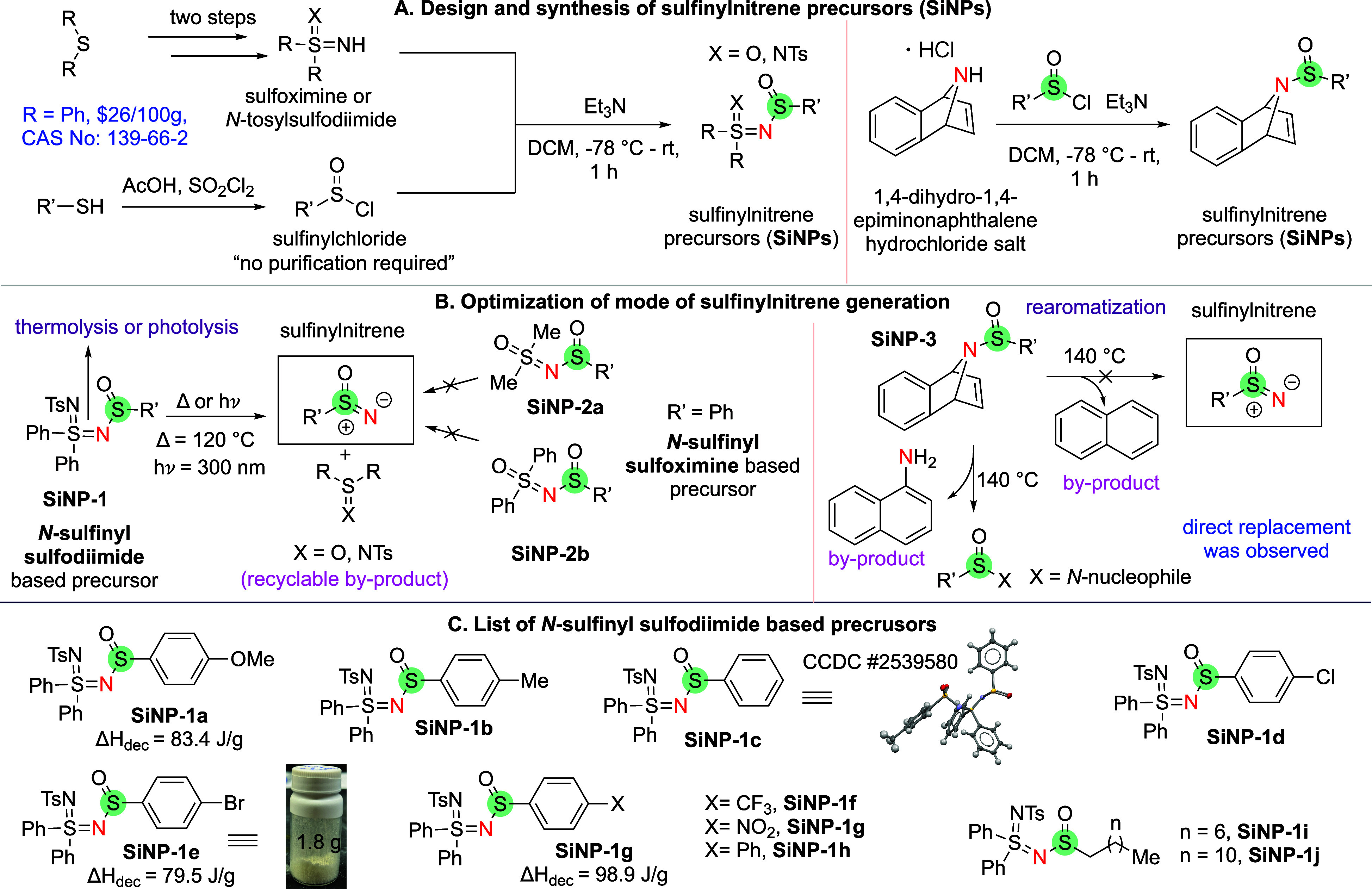
Design, Synthesis, and Optimization for the Sulfinylnitrene Generation

To generate the sulfinylnitrene from these SiNPs precursors, we explored different activation methods (Scheme 2B). Our study found that *N*-sulfinylated sulfodiimides (**SiNP-1**) can produce sulfinylnitrenes through chemoselective thermolysis or photolysis of the S=N bond. However, the corresponding *N*-sulfinylated sulfoximine (**SiNP-2**) could not be activated under either condition. In addition to different activation modes, the formation of recyclable byproducts highlights the efficiency of these precursors (see SI section 4 for details). However, the naphthalene-based design (**SiNP-3**), which efficiently generated sulfenylnitrenes in our previous work, failed to generate sulfinylnitrene, forming a directly replaced product as observed in Maricich’s work.^29^

SiNPs (**SiNP-1a** to **SiNP-1j**) bearing electron-rich (OMe), electron-deficient (CF_3_, NO_2_), halogens (Cl, Br), biphenyl, or long-chain alkyl groups were successfully synthesized using this methodology (Scheme 2C). Furthermore, the structure of **SiNP-1c** was elucidated by X-ray crystallography (CCDC 2539580). The robustness of this sulfinylnitrene precursor synthesis methodology was showcased by the gram-scale synthesis of **SiNP-1e**. Next, we optimized the generation of the sulfinylnitrene in the presence of an amine to access the corresponding sulfonimidamide (Scheme 3). Commercially available piperidine was selected as the model substrate for optimization. Heating **SiNP-1c** (1.0 equiv) at 120 °C in chlorobenzene (0.1 M) in the presence of piperidine (1.5 equiv) resulted in quantitative formation of the desired sulfonimidamide (Scheme 3A). We also screened a range of solvents and found that the reaction performed equally well in dioxane, toluene, dimethylformamide, and *n*-butyl acetate. However, a significant decrease in yield was observed when a tertiary alcohol was used as the solvent, presumably due to the sulfinylnitrene interfering with the alcohol’s acidic proton. Overall, *n*-butyl acetate was selected as the optimal solvent under thermal conditions, owing to its classification as a green solvent.^54^

**Scheme 3:**
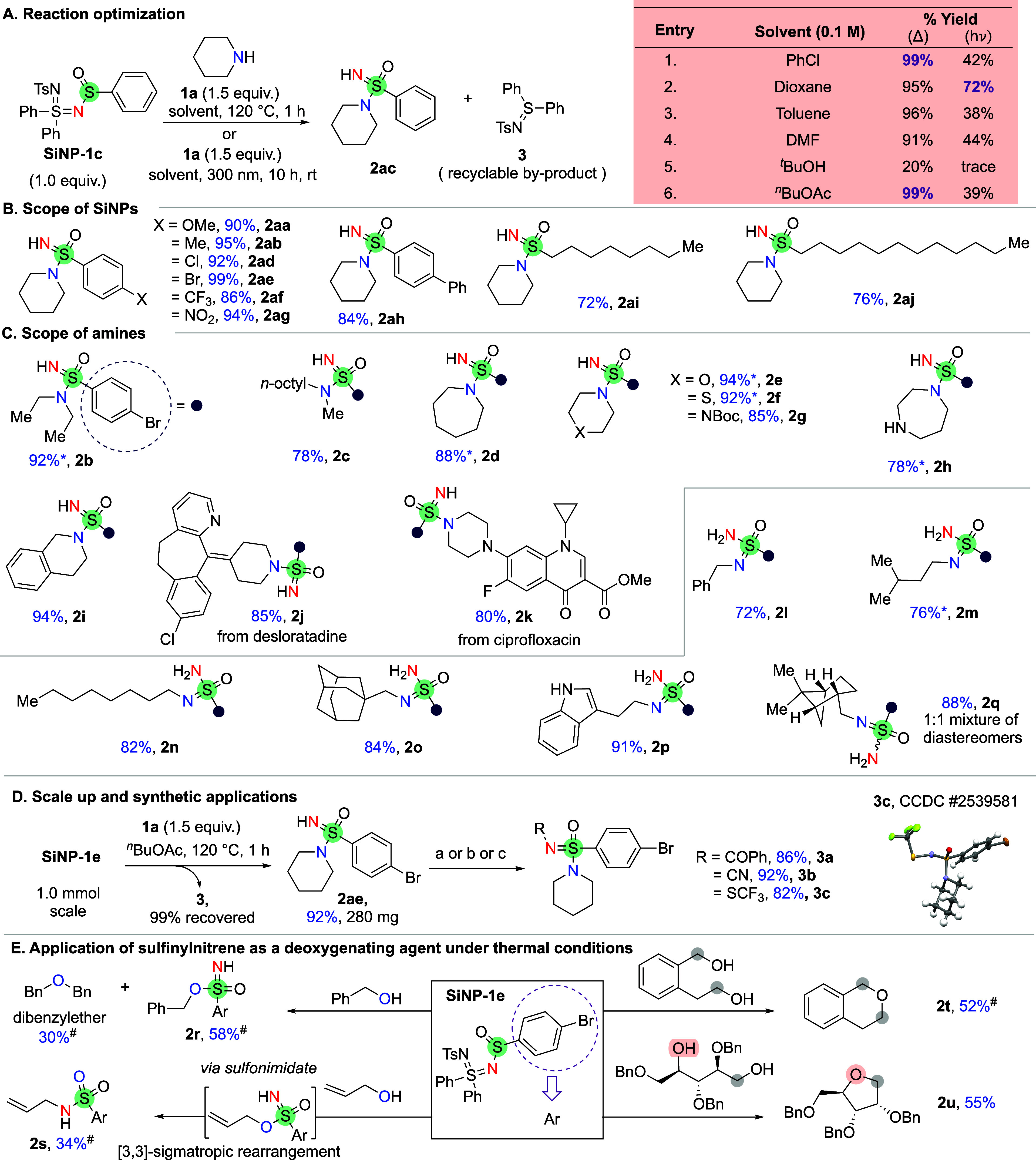
Reaction Optimization and Scope of Sulfinylnitrene Precursors^a^

a Reactions were carried out on a 0.02–1.0 mmol scale in a sealed vial. SiNPs (1.0 equiv), piperidine (1.5 equiv), solvent (0.1 M). Reaction conditions: a) NaH, BzCl; b) NaH, BrCN; c) NBS, AgSCF_3_. *SiNP-1e was used as a limiting reagent. ^#^NMR yield.

We also examined the reaction optimization under photolytic conditions. Compared with the thermal protocol, the photochemical conditions (300 nm) consistently yielded lower yields, underscoring its relatively lower efficiency. Despite these limitations, solvent screening identified dioxane as the optimal solvent for photolytic activation, affording a moderate yield. (Scheme 3A).

Since the thermal conditions outperformed the photolytic conditions, they were chosen as the optimal mode for nitrene generation. **SiNP-1a** to **SiNP-1g** precursors bearing electron-donating (OMe, Me), electron-withdrawing (CF_3_, NO_2_), halogens (Cl, Br) groups were thermally activated to generate sulfinylnitrene and trapped with piperidine to afford the corresponding sulfonimidamides in excellent yields (Scheme 3B). Biphenyl-based precursor **SiNP-1h** and long-chain alkyl-based precursors (**SiNP-1i** and **SiNP-1j**) also afforded the corresponding products in good yields.

**SiNP-1e** was selected as the precursor for the substrate scope study of amines. For amines with low boiling points, sulfinylnitrene precursor was used as the limiting reagent, while for higher-boiling-point amines, the amine was used as the limiting reagent. We began exploring the scope of amines with noncyclic secondary amines. Both diethylamine and octylmethylamine successfully reacted, affording the desired products **2b** and **2c,** respectively, in good yields (Scheme 3C). Cyclic secondary amines such as azepane (**2d**), morpholine (**2e**), and thiomorpholine (**2f**), which contain oxidatively sensitive thioether groups, were well tolerated. 1-Boc-piperazine was also reacted with a sulfinylnitrene to obtain the desired sulfonimidamide (**2g**), demonstrating compatibility with the carbamate protecting group. When 1,4-diazacycloheptane was used at 1.5 equiv. in the presence of a sulfinylnitrene precursor as a limiting reagent, selective amine functionalization was achieved (**2h**). This methodology enabled late-stage functionalization of the tetrahydroisoquinoline (**2i**), antihistamine desloratadine (**2j**), and the antibiotic ciprofloxacin (**2k**), demonstrating its tolerance for heterocycles, cyclopropanes, alkenes, and esters.

We also examined the reactivity of primary amines, including benzylamine, isoamylamine, 1-octylamine, and 1-adamantanemethylamine, which efficiently reacted with sulfinylnitrene to furnish the desired products (**2l**–**2o**). Tryptamine was also efficiently functionalized (**2p**), demonstrating that the transformation is compatible with the indole NH group. When a chiral amine like (−)-cis-myrtanylamine was used as a nucleophile, a diastereomeric mixture of products (**2q**) was formed, as the newly formed sulfur center is chiral.

We further evaluated the robustness of this reaction by performing it on a millimole scale (Scheme 3D). The obtained sulfonimidamide **2af** was functionalized with various electrophiles under basic conditions. Furthermore, **2af** was transformed into *N*-trifluoromethylthiolated sulfonimidamide (**3c**) (CCDC 2539581), which is known for its antitubercular activity.^55^ The application of sulfinylnitrene as a useful reactive intermediate in deoxygenation reaction was further evaluated by reacting it with alcohols as nucleophiles to access versatile sulfonimidates (**2r**) in moderate yield, along with ether as a byproduct (Scheme 3E) (see SI for details).^56^

When allyl alcohol is used as a nucleophile, the formed sulfonimidate undergoes a [3,3]-sigmatropic rearrangement to afford the alkylated sulfonamide (**2s**) (see SI for details). Using SiNP as a limiting reagent in the presence of bis-nucleophiles, direct replacement of in situ generated sulfonimidate was achieved to access cyclic ethers (**2t**–**2u**). Furthermore, the robustness of this reactivity was used to synthesize 1-deoxyribose without racemization at the C-4 center (**2u**).

In summary, these sulfinylnitrene precursors can be synthesized on a large scale and activated efficiently under thermal conditions to generate sulfinylnitrene. These reactive intermediates react with various amines to install medicinally or agrochemically relevant sulfonimidamide functionality. Preliminary results indicate that these precursors also show potential as a deoxygenating reagent. This operationally simple, metal-free methodology enables the installation of polar, three-dimensional functionality, thereby expanding access to uncharted chemical space in drug discovery.

## Supplementary Material



## Data Availability

The data underlying this study are available in the published article and its Supporting Information.

## Notes

Experimental procedures and NMR spectroscopic and analytical data for all compounds (PDF)
