# Supplementary material for: Sulfinylnitrenes via Chemoselective S–N Bond Cleavage: Design, Synthesis, and Applications
Source: Org Lett. 2026 May 14;28(21):6791–6. doi: 10.1021/acs.orglett.6c01673 (PMC13220345; doi:10.1021/acs.orglett.6c01673)
Supplement: Supplementary file 1 [file ol6c01673_si_001.pdf]

Supporting Information for

Sulfinylnitrenes via Chemoselective S-N Bond Cleavage: Design, Synthesis, and Applications

Prakash Kafle[a], Rishav Mukherjee[a], Shuhei Yasuda[a], and Indrajeet Sharma*[a]

*Corresponding author: Indrajeet Sharma, isharma@ou.edu
Webpage: https://indrajeetsharma.com

[a]Department of Chemistry and Biochemistry, University of Oklahoma, 101 Stephenson

Parkway, Norman, Oklahoma-73019-5251, United States

Table of Contents

1. Materials and Methods 2

2. Synthesis and Characterization of Sulfinylnitrene Precursors 3

    2.1 General procedure for the synthesis of sulfinyl chloride
    2.2 Characterization of Sulfinylnitrene Precursors-1 (**SiNPs**)
    2.3 Synthesis of **SiNP-2a**
    2.4 Synthesis of **SiNP-2b**
    2.5 Synthesis of **SiNP-3**
    2.6 Differential Scanning Calorimetry of Sulfinylnitrene Precursors (**SiNPs**)

3 General Procedure for the Amine Insertion into Sulfinylnitrenes 14

4 Scale-up and Post-modifications 25

    4.1 Reaction scale-up

    4.2 Flow chart for the recycling of the by-product

    4.3 Mechanism for the deoxygenation by sulfinylnitrene

    4.4 Limitations of the methodology

5 Data for Single X-ray Diffraction 28

6 DFT Calculations on the Bond Length and Singlet-Triplet Energy Gap 32

7 References 34

8 NMR Spectra 35

1. Materials and Methods

Reagents

Reagents and solvents were obtained from Sigma-Aldrich (www.sigma-aldrich.com), ChemImpex (www.chemimpex.com) or Acros Organics (www.fishersci.com) and used without further purification unless otherwise indicated. Dry solvents (acetonitrile) were obtained from Acros Organics (www.fishersci.com), and dichloromethane was distilled over CaH_2 under N_2 unless otherwise indicated. THF purchased from Sigma-Aldrich was distilled over Na metal with benzophenone indicator.

Reactions

All reactions were performed in flame-dried glassware under positive N_2 pressure with magnetic stirring unless otherwise noted. Cold baths were generated as follows: 0 °C with wet ice/water, –5 °C with Julabo (MeOH as a coolant), and −78 °C with dry ice/acetone. Syringe pump addition reactions were conducted using a CMA/100 microinjection pump.

Chromatography

TLC was performed on 0.25 mm E. Merck silica gel 60 F254 plates and visualized under UV light (254 nm) or by staining with potassium permanganate ($KMnO_4$), cerium ammonium molybdenate (CAM), phosphomolybdic acid (PMA), and ninhydrin. Silica flash chromatography was performed on Sorbtech 230–400 mesh silica gel 60.

Analytical Instrumentation

NMR spectra were recorded on a Varian VNMRS 400, 500, and 600 MHz NMR spectrometer in $CDCl_3$ unless otherwise indicated. Chemical shifts are expressed in ppm relative to solvent signals: $CDCl_3$ (^1H, 7.26 ppm, ^{13}C, 77.16 ppm); coupling constants are expressed in Hz. NMR spectra were processed using Mnova (www.mestrelab.com/software/mnova-nmr). Mass spectra were obtained using an Advion TLC-Mass Spectrometry (ESI).

2. **Synthesis of Sulfinylnitrene Precursors (SiNPs)**

2.1 General procedure for the synthesis of sulfinyl chloride

General procedure-1 (GP1):

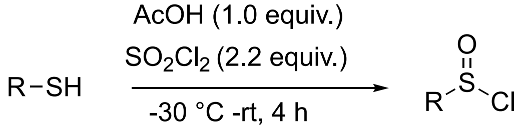

To a thiol (1 equiv.) in a round bottom flask under N_2, acetic acid (1 equiv.) was added, and the resulting solution was cooled to -30 °C. To the solidified reaction mixture upon cooling, SO_2Cl_2 (2.2 equiv.) was added dropwise over 5 min and the resulting mixture was stirred at -30 °C for 30 min. The resulting solution was slowly warmed to room temperature over the period of 1 hour and stirred at room temperature for 2 hours. After the completion of reaction, all the volatiles were removed under reduced pressure (bath temperature 20 °C) and used immediately without further purification. (All the aromatic sulfinyl chloride were synthesized using GP1).

General procedure-2 (GP2):

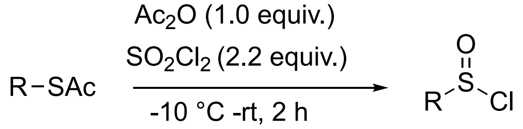

To a thioacetate (1 equiv.) in a round bottom flask under N_2 at -10 °C, acetic anhydride (1 equiv.) was added. To the resulting mixture, SO_2Cl_2 (2.2 equiv.) was added dropwise over 5 min. The resulting solution was slowly warmed to room temperature over the period of 30 min and stirred at room temperature for 1 hour. After the completion of reaction, all the volatiles were removed under reduced pressure (bath temperature 20 °C) and used immediately without further purification. (All the aliphatic sulfinyl chloride were synthesized using GP2).

2.2 Synthesis of SiNP-1

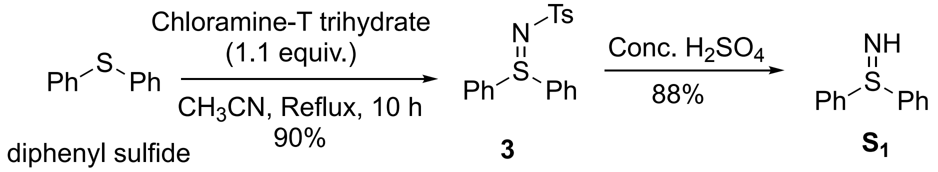

Step-1: To a solution of diphenyl sulfide (26.8 mmol) in 100 mL of CH_3CN was added chloramine T trihydrate (29.5 mmol), and the resulting solution was refluxed for 10 h. After the completion of reaction, CH_3CN was dried under reduced pressure, and the resulting solid was dissolved in dichloromethane. The resulting solution was filtered, and the filtrate was concentrated under reduced pressure. The crude solid was recrystallized from ethyl acetate/hexane to obtain compound **3** as white needle solid (8.6g, 90% yield). The spectral data of product **3** matched with previously reported literature.[1]

^{1}H NMR: (400 MHz, $CDCl_3$) δ 7.74 (d, J = 7.8 Hz, 2H), 7.62 (d, J = 7.7 Hz, 4H), 7.47 (dt, J = 14.9, 7.2 Hz, 6H), 7.14 (d, J = 7.9 Hz, 2H), 2.34 (s, 3H).

^{13}C NMR: (100 MHz, $CDCl_3$) δ 141.8, 141.4, 136.6, 132.4, 130.0, 129.3, 127.4, 126.4, 21.5.

Step-2: To a 50 mL round bottom flask charged with compound **3** (24.2 mmol) at 0 °C, was added 25 mL concentrated H_2SO_4 dropwise. It was then stirred at room temperature for the next 1 hour, during which all solid compound dissolved. The reaction mixture was then added dropwise to an ice-cold water solution over 5 minutes, and the resulting aqueous solution was washed with dichloromethane. The organic layer was back-extracted with water and combined with rest of the aqueous layer. The resulting aqueous solution was basified to pH 10 using 3N NaOH in ice-cold conditions and extracted with dichloromethane (3 times). The organic portion was washed with brine, dried over Na_2SO_4, and concentrated under reduced pressure to obtain compound **S_1** as a white crystalline solid (4.1 g, 88% yield). The spectral data of product **S_1** matched with previously reported literature.[2]

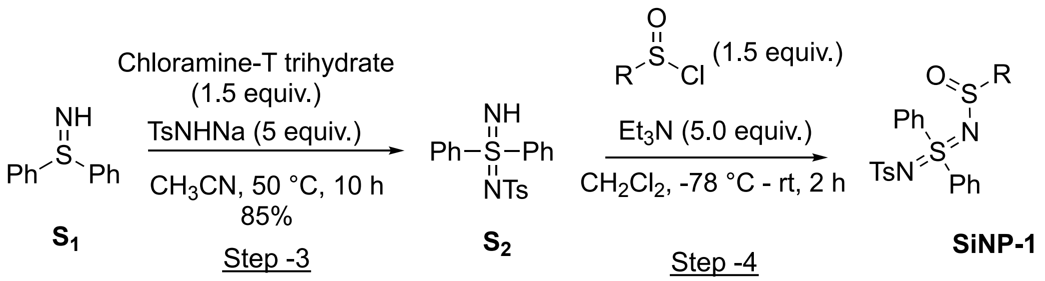

Step-3: To a solution of compound **S_1** (5.0g, 25.34 mmol) in CH_3CN (0.2 M) was added chloramine T trihydrate (10.71g), TsNHNa (24.47g), and heated to 50 °C for 10 h. After completion, the resulting mixture was diluted with dichloromethane. The mixture was then filtered, and the filtrate was washed with saturated aq. $NaHCO_3$, brine, and dried over Na_2SO_4. It was then concentrated under reduced pressure and purified by silica-gel column chromatography to obtain compound **S_2** as a white solid (8.05g, 85% yield, R_f = 0.2 in 50% EtOAc/Hexane). The spectral data of product **S_2** matched with previously reported literature.[3]
^{1}H NMR: (400 MHz, $CDCl_3$) δ 8.08 – 8.00 (m, 4H), 7.80 – 7.75 (m, 2H), 7.55 – 7.49 (m, 2H), 7.49 – 7.40 (m, 4H), 7.16 – 7.11 (m, 2H), 3.01 (s, 1H), 2.33 (s, 3H).
^{13}C NMR: (100 MHz, $CDCl_3$) δ 142.3, 141.6, 141.4, 133.2, 129.5, 129.2, 127.6, 126.8, 21.5.

General procedure-3 (GP3)

Step-4: To a solution of **S_2** (1.0 equiv.) in CH_2Cl_2 (0.1 M), Et_3N (5.0 equiv.) was added at -78 °C followed by the addition of sulfinyl chloride (1.5 equiv.) as a CH_2Cl_2 solution (0.5 M) (synthesized using general procedure GP1 or GP2). The resulting solution was slowly warmed to room temperature and stirred at room temperature for 1 h until the complete consumption of compound **S_2**. The reaction mixture was diluted with dichloromethane and washed with aq. saturated $NaHCO_3$ (3 times), water (2 times), brine, and dried over Na_2SO_4 and purified by silica-gel column chromatography to obtain **SiNP-1.**

2.2 Characterization of Sulfinylnitrene Precursors-1 (SiNPs)

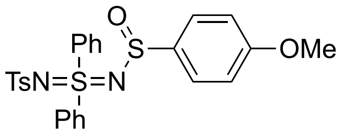

SiNP-1a

***N*-((((4-Methoxyphenyl)sulfinyl)imino)diphenyl-λ6-sulfaneylidene)-4 methylbenzenesulfonamide (SiNP-1a):** Synthesized according to GP3. The compound **SiNP-1a** was obtained as a white solid. (melting point: 137-138 °C; 390 mg, 92% yield, R_f = 0.2 in 40% EtOAc/Hexane).

^{1}H NMR: (400 MHz, $CDCl_3$)δ 8.15 (d, *J* = 7.9 Hz, 2H), 7.96 (d, *J* = 7.9 Hz, 2H), 7.81 (d, *J* = 8.8 Hz, 2H), 7.63 (dd, *J* = 20.4, 7.7 Hz, 3H), 7.55 (t, *J* = 7.9 Hz, 3H), 7.44 (t, *J* = 7.9 Hz, 2H), 7.09 (d, *J* = 8.2 Hz, 2H), 7.00 (d, *J* = 8.6 Hz, 2H), 3.87 (s, 3H), 2.33 (s, 3H).

^{13}C NMR:(100 MHz, $CDCl_3$) δ 161.9, 142.9, 140.2, 140.0, 138.6, 138.1, 134.3, 133.8, 129.8, 129.7, 129.3, 128.8, 128.2, 127.2, 126.8, 114.3, 55.6, 21.6.

LRMS(ESI): calculated $C_{26}H_{24}N_2NaO_4S_3(M+Na)^+$:547.1 ; found:547.0

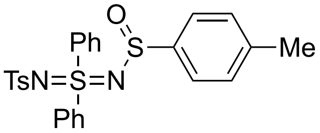

SiNP-1b

***N*-(Diphenyl((*p*-tolylsulfinyl)imino)-λ6-sulfaneylidene)-4-methylbenzenesulfonamide (SiNP-1b):** Synthesized according to GP3. The compound **SiNP-1b** was obtained as a white solid. (melting point: 144-146 °C; 370 mg, 89% yield, R_f = 0.25 in 40% EtOAc/Hexane).

^{1}H NMR: (400 MHz, $CDCl_3$) δ 8.18 – 8.11 (m, 2H), 7.99 – 7.92 (m, 2H), 7.79 – 7.73 (m, 2H), 7.68 – 7.59 (m, 3H), 7.59 – 7.52 (m, 3H), 7.48 – 7.41 (m, 2H), 7.34 – 7.29 (m, 2H), 7.11 – 7.06 (m, 2H), 2.43 (s, 3H), 2.33 (s, 3H).

^{13}C NMR:(100 MHz, $CDCl_3$) δ 142.9, 141.4, 140.2, 138.6, 138.1, 134.3, 133.9, 132.4, 129.8, 129.7, 129.6, 129.3, 128.8, 128.2, 126.8, 125.5, 21.6, 21.6.

LRMS(ESI): calculated $C_{26}H_{24}N_2NaO_3S_3(M+Na)^+$:531.1 ; found:531.2.

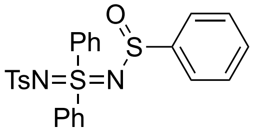

SiNP-1c

***N*-(Diphenyl((phenylsulfinyl)imino)-λ6-sulfaneylidene)-4-methylbenzenesulfonamide (SiNP-1c):** Synthesized according to GP3. The compound **SiNP-1c** was obtained as a white solid. (melting point: 148-149 °C; 340 mg, 84% yield, R_f = 0.25 in 40% EtOAc/Hexane).

^{1}H NMR: (400 MHz, $CDCl_3$) δ 8.19 – 8.13 (m, 2H), 7.97 – 7.93 (m, 2H), 7.91 – 7.86 (m, 2H), 7.67 – 7.60 (m, 3H), 7.59 – 7.53 (m, 3H), 7.51 (dd, *J* = 5.3, 2.0 Hz, 3H), 7.47 – 7.42 (m, 2H), 7.08 (d, *J* = 8.2 Hz, 2H), 2.33 (s, 3H).

^{13}C NMR:(100 MHz, $CDCl_3$) δ 148.4, 143.0, 140.2, 138.6, 138.1, 134.4, 133.9, 131.0, 129.8, 129.7, 129.3, 128.9, 128.8, 128.2, 126.8, 125.5, 21.6.
LRMS(ESI): calculated $C_{25}H_{22}N_2NaO_3S_3(M+Na)^+$:517.1 ; found:517.2.

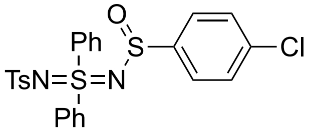

SiNP-1d

***N*-((((4-Chlorophenyl)sulfinyl)imino)diphenyl-λ6-sulfaneylidene)-4-methylbenzene sulfon amide (SiNP-1d):** Synthesized according to GP3. The compound **SiNP-1d** was obtained as a white solid. (melting point: 140-142 °C; 160 mg, 75% yield, $R_f = 0.25$ in 40% EtOAc/Hexane).
^{1}H NMR: (400 MHz, $CDCl_3$) δ 8.14 (d, *J* = 7.8 Hz, 2H), 7.93 (d, *J* = 7.9 Hz, 2H), 7.84 (d, *J* = 8.6 Hz, 2H), 7.68 – 7.61 (m, 3H), 7.61 – 7.53 (m, 3H), 7.47 (ddd, *J* = 8.3, 5.1, 3.0 Hz, 4H), 7.10 (d, *J* = 8.2 Hz, 2H), 2.35 (s, 3H).
^{13}C NMR:(100 MHz, $CDCl_3$) δ 143.5, 141.9, 139.9, 137.0, 136.7, 134.9, 130.2, 130.0, 129.6, 128.6, 128.5, 128.2, 127.8, 127.4, 126.9, 126.4, 21.7.
LRMS(ESI): calculated $C_{25}H_{21}ClN_2NaO_3S_3(M+Na)^+$:551.1 ; found:551.2.

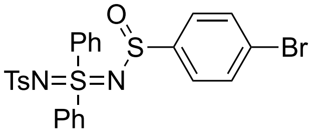

SiNP-1e

***N*-((((4-Bromophenyl)sulfinyl)imino)diphenyl-λ6-sulfaneylidene)-4-methylbenzenesulfon amide (SiNP-1e):** Synthesized according to GP3. The compound **SiNP-1e** was obtained as a white solid. (melting point: 142-143 °C; 1.8g, 85% yield, $R_f = 0.25$ in 40% EtOAc/Hexane).
^{1}H NMR: (400 MHz, $CDCl_3$) δ 8.13 (dd, *J* = 7.8, 1.9 Hz, 2H), 7.92 (d, *J* = 7.7 Hz, 2H), 7.80 – 7.73 (m, 2H), 7.65 – 7.62 (m, 5H), 7.56 (t, *J* = 7.7 Hz, 3H), 7.45 (t, *J* = 8.0 Hz, 2H), 7.10 (d, *J* = 8.3 Hz, 2H), 2.35 (s, 3H).
^{13}C NMR:(100 MHz, $CDCl_3$) δ 147.5, 143.2, 140.0, 138.4, 137.7, 134.5, 134.0, 132.1, 129.9, 129.7, 129.3, 128.7, 128.2, 127.3, 126.7, 125.7, 21.7.
LRMS(ESI): calculated $C_{25}H_{21}BrN_2NaO_3S_3(M+Na)^+$:595.0 ; found:595.3.

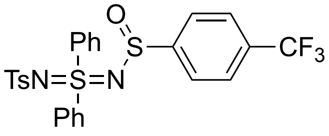

SiNP-1f

***N*-(Diphenyl(((4-(trifluoromethyl)phenyl)sulfinyl)imino)-λ6-sulfaneylidene)-4-methyl benzenesulfonamide (SiNP-1f):** Synthesized according to GP3. The compound **SiNP-1f** was

obtained as a white solid. (melting point: 138-140 °C; 160 mg, 75% yield, R_f = 0.2 in 40% EtOAc/Hexane).

^{1}H NMR: (400 MHz, $CDCl_3$) δ 8.17 – 8.10 (m, 2H), 8.05 (d, J = 7.9 Hz, 2H), 7.93 – 7.87 (m, 2H), 7.77 (d, J = 8.3 Hz, 2H), 7.67 – 7.51 (m, 6H), 7.44 (t, J = 7.7 Hz, 2H), 7.10 (d, J = 8.2 Hz, 2H), 2.33 (s, 3H).

^{13}C NMR:(100 MHz, $CDCl_3$) δ 152.4, 143.2, 140.0, 138.4, 137.6, 134.6 (d, J= 42.8 Hz)), 133.0, 132.7, 130.0, 129.5, 129.3, 128.7, 128.2, 126.2 (q, J= 3.7 Hz), 125.2, 122.5, 21.56.

^{19}F NMR: (376 MHz, $CDCl_3$) δ -62.5.

LRMS(ESI): calculated $C_{26}H_{21}F_3N_2NaO_3S_3(M+Na)^+$:585.1 ; found:585.3.

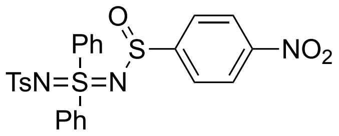

SiNP-1g

4-Methyl-*N*-((((4-nitrophenyl)sulfinyl)imino)diphenyl-λ6-sulfaneylidene)benzenesulfon amide (SiNP-1g): Synthesized according to GP3. The compound **SiNP-1g** was obtained as a white solid. (melting point: 141-142 °C; 420 mg, 96% yield, R_f = 0.1 in 40% EtOAc/Hexane).

^{1}H NMR: (400 MHz, $CDCl_3$) δ 8.39 – 8.32 (m, 2H), 8.17 – 8.11 (m, 4H), 7.90 – 7.85 (m, 2H), 7.70 – 7.55 (m, 6H), 7.45 (td, J = 8.0, 1.6 Hz, 2H), 7.12 (d, J = 8.0 Hz, 2H), 2.35 (s, 3H).

^{13}C NMR:(100 MHz, $CDCl_3$) δ 155.2, 149.5, 143.4, 139.9, 138.3, 137.3, 134.7, 134.2, 130.0, 129.8, 129.4, 128.6, 128.1, 127.0, 126.7, 124.1, 21.7.

LRMS(ESI): calculated $C_{25}H_{21}N_2NaO_5S_3(M+Na)^+$:562.0 ; found:562.4.

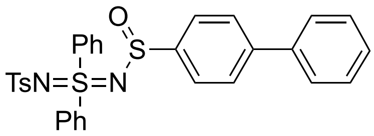

SiNP-1h

***N*-((([1,1'-Biphenyl]-4-ylsulfinyl)imino)diphenyl-λ6-sulfaneylidene)-4-methyl benzenesulfon amide (SiNP-1h):** Synthesized according to GP3. The compound **SiNP-1h** was obtained as a white solid. (melting point: 140-142 °C; 180 mg, 78% yield, R_f = 0.2 in 40% EtOAc/Hexane).

^{1}H NMR: (400 MHz, $CDCl_3$) δ 8.17 (dd, J = 7.8, 1.8 Hz, 2H), 7.97 (ddd, J = 6.7, 3.2, 2.0 Hz, 4H), 7.73 (dd, J = 8.4, 1.8 Hz, 2H), 7.70 – 7.60 (m, 5H), 7.60 – 7.52 (m, 3H), 7.51 – 7.38 (m, 5H), 7.10 (d, J = 8.3 Hz, 2H), 2.32 (s, 3H).

^{13}C NMR:(100 MHz, $CDCl_3$) δ 147.2, 143.9, 143.0, 140.3, 140.1, 138.5, 137.9, 134.4, 133.9, 129.8, 129.7, 129.3, 129.0, 128.7, 128.2, 128.0, 127.6, 127.4, 126.7, 126.0, 21.6.

LRMS(ESI): calculated $C_{31}H_{26}N_2NaO_3S_3(M+Na)^+$:593.1 ; found:593.0.

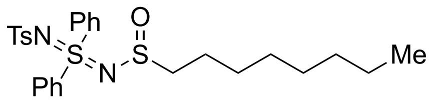

SiNP-1i

4-Methyl-*N*-(((octylsulfinyl)imino)diphenyl-λ6-sulfaneylidene)benzenesulfonamide (SiNP-1i): Synthesized according to GP3. The compound **SiNP-1i** was obtained as a clear oil. (232 mg, 88% yield, R_f = 0.2 in 40% EtOAc/Hexane).
^{1}H NMR: (400 MHz, $CDCl_3$) δ 8.13 – 8.06 (m, 2H), 8.02 – 7.95 (m, 2H), 7.73 – 7.67 (m, 2H), 7.64 – 7.57 (m, 1H), 7.57 – 7.50 (m, 3H), 7.49 – 7.41 (m, 2H), 7.15 (d, *J* = 8.3 Hz, 2H), 2.90 (ddd, *J* = 12.9, 9.5, 5.7 Hz, 1H), 2.76 (ddd, *J* = 12.9, 9.5, 6.6 Hz, 1H), 2.36 (s, 3H), 1.82 – 1.67 (m, 2H), 1.42 – 1.24 (m, 10H), 0.94 – 0.83 (m, 3H).
^{13}C NMR:(100 MHz, $CDCl_3$) δ 143.0, 140.4, 138.1, 137.8, 134.4, 133.8, 129.8, 129.4, 129.2, 128.9, 128.3, 126.8, 59.3, 31.9, 29.5, 29.3, 28.9, 22.8, 22.0, 21.6, 14.3.
LRMS(ESI): calculated $C_{27}H_{34}N_2NaO_3S_3(M+Na)^+$:533.2 ; found:533.6.

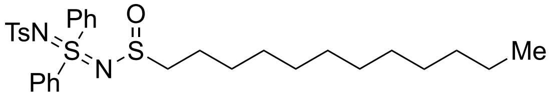

SiNP-1j

***N*-(((Dodecylsulfinyl)imino)diphenyl-λ6-sulfaneylidene)-4-methylbenzenesulfonamide (SiNP-1j):** Synthesized according to GP3. The compound **SiNP-1j** was obtained as a clear oil. (180 mg, 85% yield, R_f = 0.2 in 40% EtOAc/Hexane).
^{1}H NMR: (400 MHz, $CDCl_3$) δ 8.12 – 8.07 (m, 2H), 8.02 – 7.95 (m, 2H), 7.73 – 7.67 (m, 2H), 7.64 – 7.58 (m, 1H), 7.58 – 7.50 (m, 3H), 7.45 (td, *J* = 7.1, 1.9 Hz, 2H), 7.15 (d, *J* = 8.2 Hz, 2H), 2.90 (ddd, *J* = 13.0, 9.5, 5.7 Hz, 1H), 2.76 (ddd, *J* = 12.9, 9.5, 6.6 Hz, 1H), 2.36 (s, 3H), 1.79 – 1.64 (m, 3H), 1.42 – 1.25 (m, 17H), 0.92 – 0.84 (m, 3H).
^{13}C NMR:(100 MHz, $CDCl_3$) δ 143.0, 140.4, 138.1, 137.8, 134.4, 133.8, 129.9, 129.4, 129.2, 128.9, 128.3, 126.8, 59.2, 32.1, 29.8, 29.8, 29.6, 29.5, 29.5, 28.9, 22.8, 22.0, 21.7, 21.7, 14.3.
LRMS(ESI): calculated $C_{31}H_{42}N_2NaO_3S_3(M+Na)^+$:609.2 ; found:609.2.

2.3 Synthesis of SiNP-2a

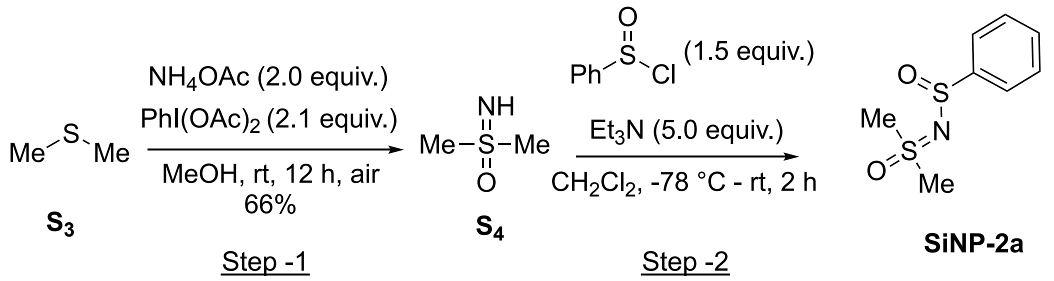

<u>Step-1:</u> To a solution of dimethyl sulfide **S_3** 300 mg (4.8 mmol, 1.0 equiv) and NH_4OAc (9.6 mmol) in MeOH (2 mL/mmol with reference to the sulfide) at rt under air, solid $PhI(OAc)_2$ (10 mmol) was added portion-wise. The resulting mixture was stirred at rt for 6 h open to the air. After the completion of reaction, the reaction mixture was concentrated under reduced pressure. The

crude mixture was diluted with CH_2Cl_2, solid $NaHCO_3$ was added, stirred 15 min followed by the addition of Na_2SO_4 and stirred another 15 min. The solid was removed by filtration and the filtrate was concentrated under reduced pressure. Purification by silica-gel chromatography afforded sulfoximine **S4** as a pale-yellow oil (295 mg, 66% yield, $R_f = 0.1$ in 40% Acetone/EtOAc). The spectral data of product **S4** matched with previously reported literature.[4]

Step-2: To a solution of **S4** (125 mg, 1.34 mmol) in CH_2Cl_2 (0.1 M), Et_3N (5.0 equiv.) was added at -78 °C followed by the addition of benzenesulfinyl chloride (323 mg) as a CH_2Cl_2 solution (0.5 M). The resulting solution was slowly warmed to room temperature and stirred at room temperature for 1 h until the complete consumption of compound **S4**. The reaction mixture was diluted with dichloromethane and washed with aq. saturated $NaHCO_3$ (3 times), water (2 times), brine, and dried over Na_2SO_4 and purified by silica-gel column chromatography to obtain **SiNP-2** as a white solid. (200 mg, 70% yield, $R_f = 0.2$ in 5% MeOH/CH_2Cl_2).

^{1}H NMR: (400 MHz, $CDCl_3$) δ 7.75 (dd, J = 7.7, 1.8 Hz, 2H), 7.54 – 7.45 (m, 3H), 3.42 (s, 3H), 3.24 (s, 3H).

^{13}C NMR:(100 MHz, $CDCl_3$) δ 148.2, 131.0, 129.1, 124.9, 45.1, 44.3.

LRMS(ESI): calculated $C_8H_{11}NNaO_2S_2(M+Na)^+$:240.0 ; found:240.1.

2.4 Synthesis of SiNP-2b

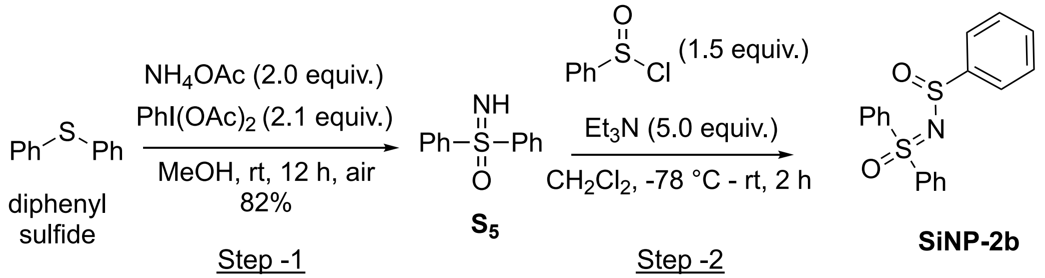

Step-1: **S6** was synthesized as previously reported.[5] To a solution of diphenyl sulfide 500 mg (2.6 mmol, 1.0 equiv) and NH_4OAc (5.3 mmol) in MeOH (2 mL/mmol with reference to sulfide) at rt under air, solid $PhI(OAc)_2$ (5.6 mmol) was added portion-wise. The resulting mixture was stirred at rt for 12 h under air. After the completion of the reaction, the reaction mixture was concentrated under reduced pressure. The crude mixture was diluted with CH_2Cl_2, solid $NaHCO_3$ was added, and the mixture was stirred for 15 min, then Na_2SO_4 was added and stirred for another 15 min. The solid was removed by filtration, and the filtrate was concentrated under reduced pressure. Purification by silica-gel chromatography afforded sulfoximine **S5** as a white solid (480 mg, 82% yield, $R_f = 0.2$ in 40% EtOAc/Hexane). The spectral data of product **S5** matched with previously reported literature.[5]

Step-2: To a solution of **S5** (100 mg, 0.46 mmol) in CH_2Cl_2 (0.1 M), Et_3N (5.0 equiv.) was added at -78 °C, followed by the addition of benzenesulfinyl chloride (111 mg, 0.69 mmol) as a CH_2Cl_2 solution (0.5M). The resulting solution was slowly warmed to room temperature and stirred for 1 h at room temperature until complete consumption of compound **S5**. The reaction mixture was diluted with dichloromethane and washed with aq. saturated $NaHCO_3$ (3 times), water (2 times), brine, and dried over Na_2SO_4 and purified by silica-gel column chromatography to obtain **SiNP-2** as a white solid. (140 mg, 89% yield, $R_f = 0.2$ in 40% EtOAc/Hexane).

^{1}H NMR: (400 MHz, $CDCl_3$) δ 8.10 (dd, J = 13.4, 7.4 Hz, 4H), 7.81 (d, J = 6.7 Hz, 2H), 7.66 – 7.55 (m, 4H), 7.55 – 7.42 (m, 5H).

^{13}C NMR:(100 MHz, $CDCl_3$) δ 148.7, 139.8, 139.4, 134.0, 133.8, 130.8, 129.9, 129.6, 129.0, 129.0, 128.2, 125.2.
LRMS(ESI): calculated $C_{18}H_{15}NNaO_2S_2$(M+Na)$^+$:364.1 ; found:364.3.

2.5 Synthesis of SiNP-3

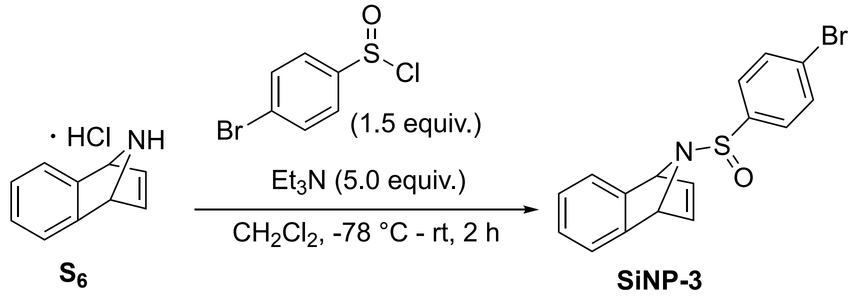

To a solution of **S_6** (100 mg, 0.5 mmol) in CH_2Cl_2 (0.1 M), Et_3N (5.0 equiv.) was added at -78 °C, followed by the addition of benzenesulfinyl chloride (200 mg, 0.8 mmol) as a CH_2Cl_2 solution (0.5M). The resulting solution was slowly warmed to room temperature and stirred for 1 h at room temperature until complete consumption of compound **S_6**. The reaction mixture was diluted with dichloromethane and washed with aq. saturated $NaHCO_3$ (3 times), water (2 times), brine, and dried over Na_2SO_4 and purified by silica-gel column chromatography to obtain **SiNP-3** as a brown solid. (145 mg, 79% yield, R_f = 0.2 in 40% EtOAc/Hexane).
^{1}H NMR: (400 MHz, $CDCl_3$) δ 7.62 (d, J = 8.5 Hz, 2H), 7.52 (d, J = 8.5 Hz, 2H), 7.27 (d, J = 4.7 Hz, 1H), 7.15 (d, J = 6.5 Hz, 1H), 7.04 (dd, J = 5.5, 2.2 Hz, 1H), 6.96 (t, J = 6.2 Hz, 3H), 5.34 (s, 1H), 5.02 (s, 1H).
^{13}C NMR:(100 MHz, $CDCl_3$) δ 149.4, 147.8, 144.0, 143.3, 141.6, 132.1, 128.0, 125.8, 125.2, 125.1, 120.5, 120.3, 68.4, 66.8.
LRMS(ESI): calculated $C_{16}H_{12}BrNNaS$(M+Na)$^+$:351.9 ; found:351.7.

2.6 Differential Scanning Calorimetry (DSC) of Sulfinylnitrene Precursors (SiNPs)

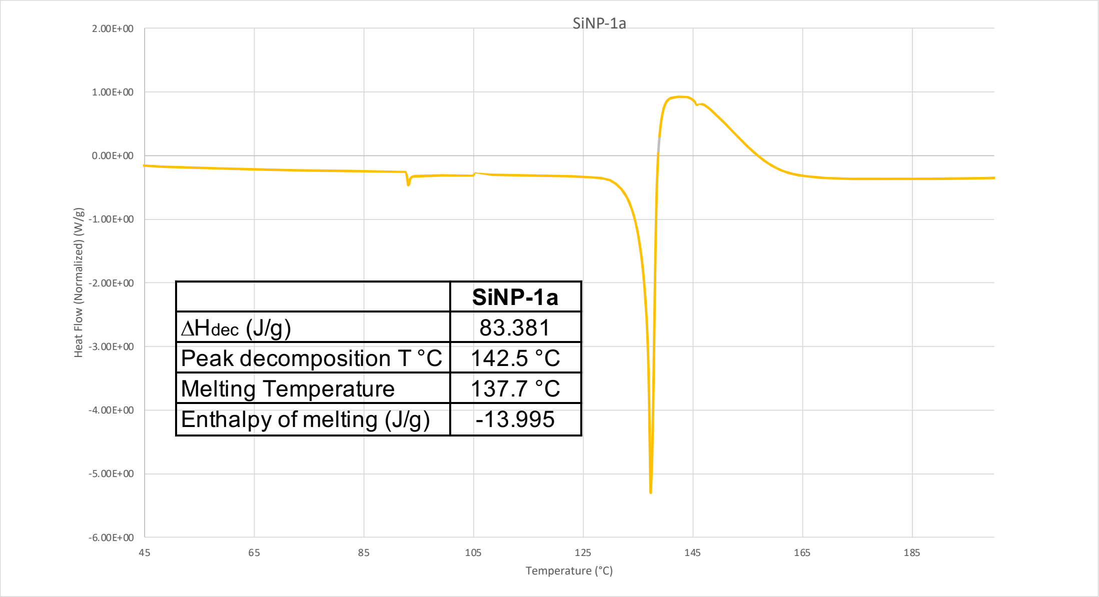

	SiNP-1a
ΔH_{dec} (J/g)	83.381
Peak decomposition T °C	142.5 °C
Melting Temperature	137.7 °C
Enthalpy of melting (J/g)	-13.995

Figure S1: DSC of SiNP-1a

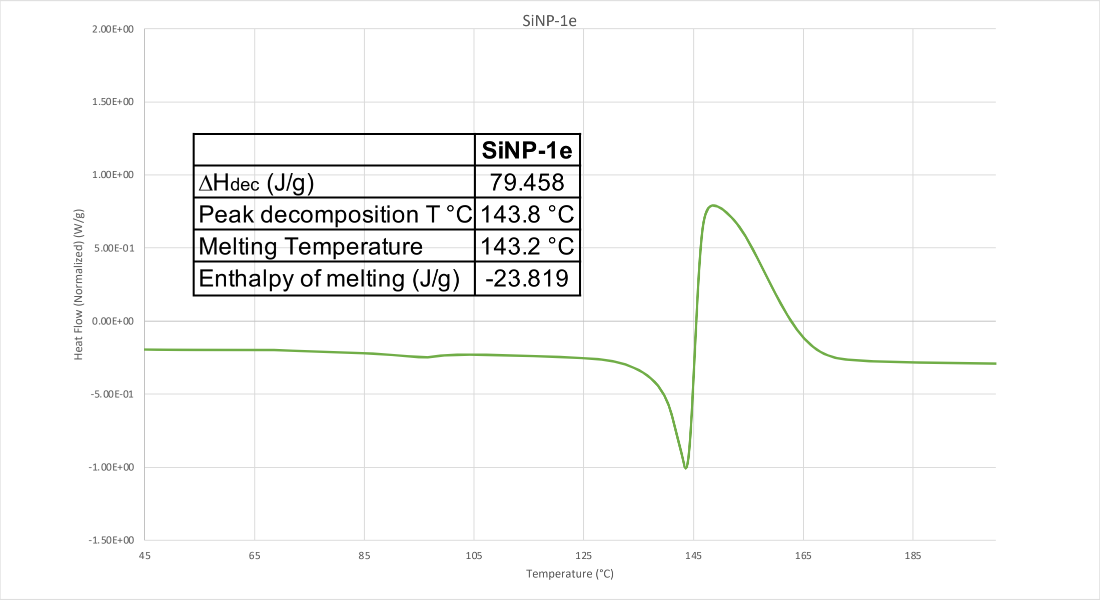

	SiNP-1e
ΔH_{dec} (J/g)	79.458
Peak decomposition T °C	143.8 °C
Melting Temperature	143.2 °C
Enthalpy of melting (J/g)	-23.819

Figure S2: DSC of SiNP-1e

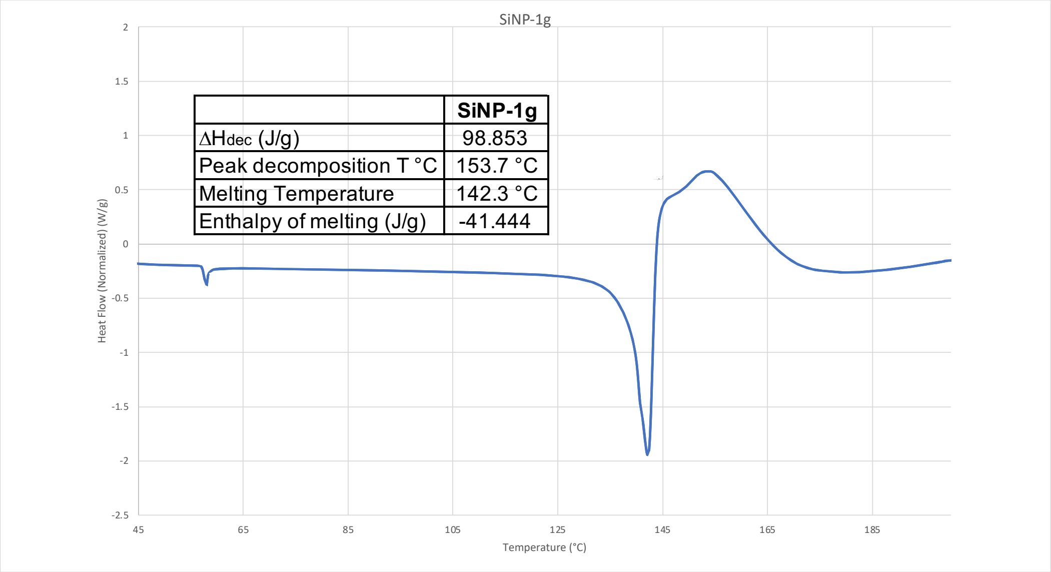

Figure S3: DSC of SiNP-1g

2.6.1 Comparison of other precursors vs SiNPs:

Literature reported common precursors for sulfinylnitrene includes BiPhONSO and sulfinylazide. As reported in literature,[6] sulfinylazide are unstable above -20 °C and explosive (neat samples) at 0 °C.

As reported in literature,[7] BiPhONSO is stored in the freezer at -20 °C and can be taken out and used regularly without loss of performance for at least three months. However, prolonged storage at room temperature should be avoided. Hydrolysis of BiPhONSO results in the formation of toxic sulfur dioxide gas, so contact with water is not advised. The literature reports that heating BiPhONSO should be avoided, as it has an enthalpy of decomposition of 1016 J/g.

In comparison, SiNPs are benchstable with no decomposition observed when stored at room temperature under air for more than 8 months. These precursors are obtained after standard aqueous workup, which further indicates that they are not sensitive to moisture or water at room temperature. The DSC data shown below shows their detailed enthalpy of decomposition and corresponding decomposition temperature.

	SiNP-1a	SiNP-1e	SiNP-1g
ΔH_{dec} (J/g)	83.381	79.458	98.853
Peak decomposition T °C	142.5 °C	143.8 °C	153.7 °C
Melting Temperature	137.7 °C	143.2 °C	142.3 °C
Enthalpy of melting (J/g)	-13.995	-23.819	-41.444

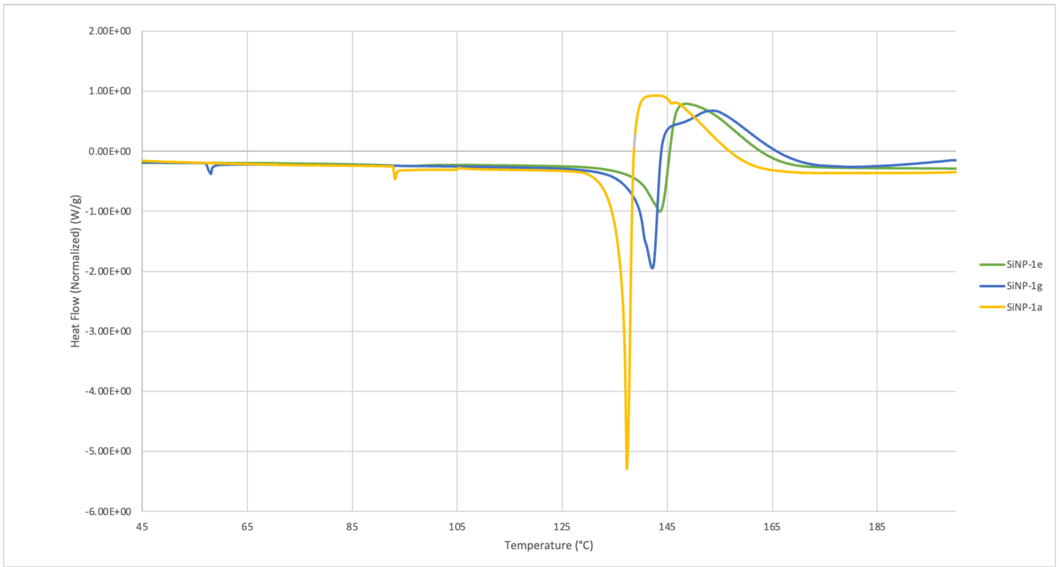

Figure S4: Overlay of DSC graphs of representative SiNPs.

3. General Procedure for the Amine Insertion into Sulfinylnitrenes

3.1 General procedure for thermal activation of sulfinylnitrene precursors

General Procedure-4 (GP4)

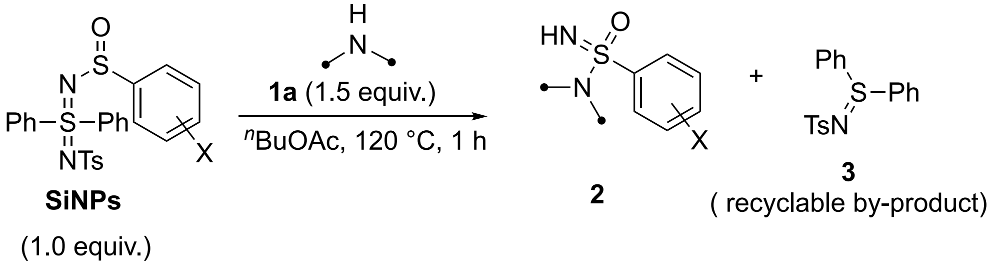

To a solution of **SiNPs** (1 equiv.) in 0.1 M *n*-butyl acetate in a capped vial, 1.5 equiv. of amine (**1**) was added, placed in a pre-heated heating mantle, and stirred for 1 hour. After completion of the reaction, all volatiles were removed under reduced pressure, and the crude mixture was purified by silica-gel chromatography.

General Procedure-5 (GP5)

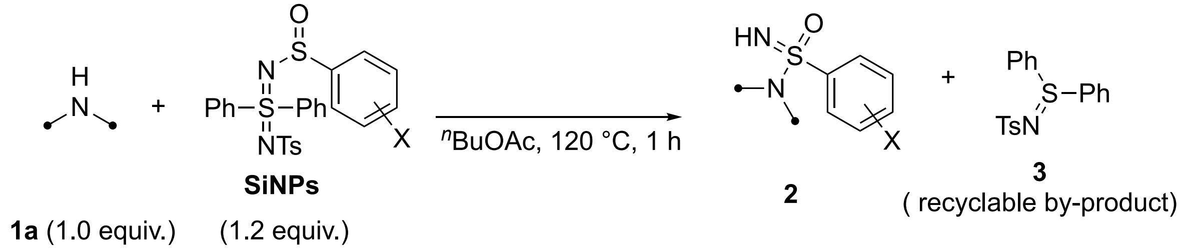

To a solution of SiNPs (1.2 equiv.) in 0.1 M *n*-butyl acetate in a capped vial, 1.0 equiv. of amine (**1a**) was added and placed in a pre-heated heating mantle for 1 hour. After completion of the reaction, all volatiles were removed under reduced pressure, and the crude mixture was purified by sílica gel chromatography.

3.2 General procedure for photolytic activation of sulfinylnitrene precursors

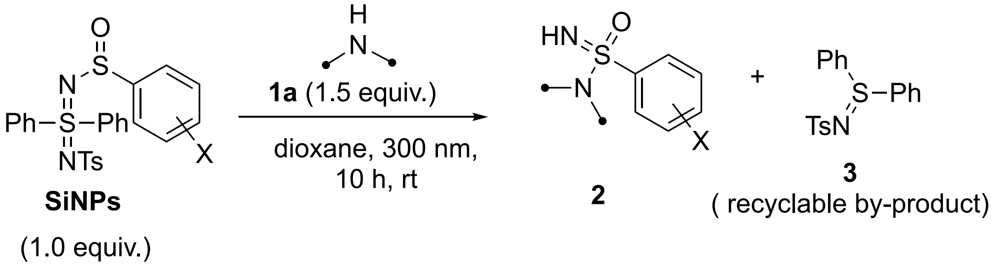

To a solution of SiNPs (1 equiv.) in 0.1 M 1,4-dioxane in a capped vial, 1.5 equiv. of amine (**1a**) was added and placed in a UV irradiation (300 nm) for 10 hour at room temperature until the

reaction was complete. After the reaction, all the volatiles were removed under reduced pressure, and the crude mixture was purified by silica-gel chromatography.

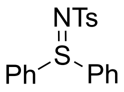

3

***N*-(Diphenyl-λ4-sulfaneylidene)-4-methylbenzenesulfonamide (3):** The spectral data of by-product matched with previously reported literature.[1]
^{1}H NMR: (400 MHz, $CDCl_3$) δ 7.74 (d, J = 7.8 Hz, 2H), 7.62 (d, J = 7.7 Hz, 4H), 7.47 (dt, J = 14.9, 7.2 Hz, 6H), 7.14 (d, J = 7.9 Hz, 2H), 2.34 (s, 3H).
^{13}C NMR: (100 MHz, $CDCl_3$) δ 141.8, 141.4, 136.6, 132.4, 130.0, 129.3, 127.4, 126.4, 21.5.

3.3 Characterization of Sulfonimidamides

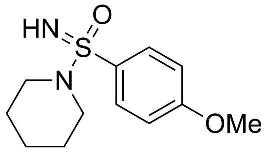

90%, **2aa**

1-(4-Methoxyphenylsulfonimidoyl)piperidine (2aa): Synthesized according to **GP4** using 20 mg (0.038 mmol, 1.0 equiv.) of **SiNP-1a** and 1.5 equiv. of piperidine. The product was obtained as off-white solid (8.7 mg, 90% yield), R_f = 0.35 in 30% ethyl acetate/hexane. The spectral data of the product matched with previously reported literature.[8]
^{1}H NMR: (400 MHz, $CDCl_3$) δ 7.80 (d, J = 8.9 Hz, 2H), 6.96 (d, J = 8.9 Hz, 2H), 3.86 (d, J = 1.5 Hz, 3H), 3.00 – 2.92 (m, 4H), 2.22 (s, 1H), 1.60 (p, J = 5.9 Hz, 4H), 1.42 – 1.32 (m, 2H).
^{13}C NMR: (100 MHz, $CDCl_3$) δ 162.7, 130.2, 127.8, 113.9, 55.7, 48.1, 25.7, 23.8.

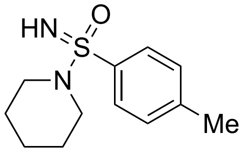

95%, **2ab**

1-(4-Methylphenylsulfonimidoyl)piperidine (2ab): Synthesized according to **GP4** using 20 mg (0.039 mmol, 1.0 equiv.) of **SiNP-1b** and 1.5 equiv. of piperidine. The product was obtained as off-white solid (8.9 mg, 95% yield), R_f = 0.35 in 30% ethyl acetate/hexane. The spectral data of the product matched with previously reported literature.[9]
^{1}H NMR: (400 MHz, $CDCl_3$) δ 7.81 – 7.76 (m, 2H), 7.31 (d, J = 8.1 Hz, 2H), 3.00 (t, J = 5.6 Hz, 4H), 2.43 (s, 3H), 2.30 (s, 1H), 1.62 (p, J = 5.9 Hz, 4H), 1.44 – 1.34 (m, 2H).
^{13}C NMR: (100 MHz, $CDCl_3$) δ 143.4, 132.8, 129.6, 128.3, 48.0, 25.7, 23.7, 21.6.

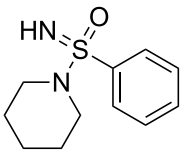

99%, **2ac**

1-(Phenylsulfonimidoyl)piperidine (2ac): Synthesized according to **GP4** using 20 mg (0.040 mmol, 1.0 equiv.) of **SiNP-1c** and 1.5 equiv. of piperidine. The product was obtained as a white solid (9.0 mg, 99% yield), $R_f = 0.35$ in 30% ethyl acetate/hexane. The spectral data of the product matched with previously reported literature. [8]

^{1}H NMR: (400 MHz, $CDCl_3$) δ 7.92 – 7.85 (m, 2H), 7.61 – 7.47 (m, 3H), 2.99 (t, J = 5.4 Hz, 4H), 1.85 (s, 1H), 1.62 (dq, J = 11.2, 5.2 Hz, 4H), 1.38 (t, J = 6.0 Hz, 2H).

^{13}C NMR: (100 MHz, $CDCl_3$) δ 136.2, 132.4, 128.8, 128.1, 48.1, 25.8, 23.7.

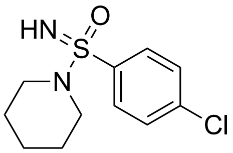

92%, **2ad**

1-(4-Chlorophenylsulfonimidoyl)piperidine (2ad): Synthesized according to **GP4** using 18 mg (0.034 mmol, 1.0 equiv.) of **SiNP-1d** and 1.5 equiv. of piperidine. The product was obtained as a white solid (8.1 mg, 92% yield), $R_f = 0.40$ in 30% ethyl acetate/hexane. The spectral data of the product matched with previously reported literature.[8]

^{1}H NMR: (400 MHz, $CDCl_3$) δ 7.82 (d, J = 8.7 Hz, 2H), 7.48 (d, J = 8.7 Hz, 2H), 2.99 (t, J = 5.6 Hz, 4H), 1.71 (s, 1H), 1.65 – 1.60 (m, 4H), 1.40 (p, J = 5.9 Hz, 2H).

^{13}C NMR: (100 MHz, $CDCl_3$) δ 142.1, 137.2, 129.2, 127.9, 47.2, 26.3, 24.0.

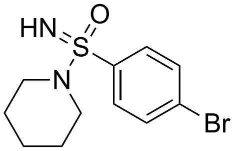

99%, **2ae**

1-(4-Bromophenylsulfonimidoyl)piperidine (2ae): Synthesized according to **GP4** using 20 mg (0.034 mmol, 1.0 equiv.) of **SiNP-1e** and 1.5 equiv. of piperidine. The product was obtained as a white solid (10.5 mg, 99% yield), $R_f = 0.30$ in 25% ethyl acetate/hexane. The spectral data of the product matched with previously reported literature.[10]

^{1}H NMR: (400 MHz, $CDCl_3$) δ 7.75 (d, J = 8.8 Hz, 2H), 7.64 (d, J = 8.7 Hz, 2H), 2.99 (t, J = 5.6 Hz, 4H), 1.88 (s, 1H), 1.62 (dq, J = 11.1, 5.3 Hz, 4H), 1.40 (td, J = 7.3, 4.3 Hz, 2H).

^{13}C NMR: (100 MHz, $CDCl_3$) δ 135.3, 132.1, 129.7, 127.4, 48.1, 25.7, 23.7.

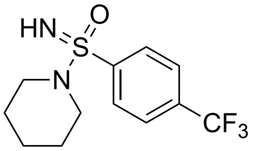

86%, **2af**

1-(4-Trifluoromethylphenylsulfonimidoyl)piperidine (2af): Synthesized according to **GP4** using 20 mg (0.035 mmol, 1.0 equiv.) of **SiNP-1f** and 1.5 equiv. of piperidine. The product was obtained as clear oil (8.9 mg, 86% yield), $R_f = 0.30$ in 30% ethyl acetate/hexane. The spectral data of the product matched with previously reported literature. [8]

^{1}H NMR: (400 MHz, $CDCl_3$) δ 8.02 (d, J = 8.3 Hz, 2H), 7.77 (d, J = 8.3 Hz, 2H), 3.05 – 2.97 (m, 4H), 1.79 (s, 1H), 1.67 – 1.60 (m, 4H), 1.40 (t, J = 5.9 Hz, 2H).

^{13}C NMR: (100 MHz, $CDCl_3$) δ 139.9, 134.3, 133.9, 128.5, 126.0 (q, J = 3.9 Hz), 48.0, 25.7, 23.6.

^{19}F NMR: (376 MHz, $CDCl_3$) δ -62.87.

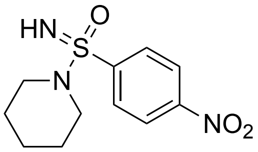

94%, **2ag**

1-(4-Nitromethylphenylsulfonimidoyl)piperidine (2ag): Synthesized according to **GP4** using 20 mg (0.037 mmol, 1.0 equiv.) of **SiNP-1g** and 1.5 equiv. of piperidine. The product was obtained as white solid (9.3 mg, 94% yield), $R_f = 0.25$ in 30% ethyl acetate/hexane. The spectral data of the product matched with previously reported literature.[11]

^{1}H NMR: (400 MHz, $CDCl_3$) δ 8.35 (d, J = 9.0 Hz, 2H), 8.07 (d, J = 9.7 Hz, 2H), 3.03 (t, J = 5.5 Hz, 4H), 1.85 (s, 1H), 1.63 (q, J = 5.8 Hz, 4H), 1.46 – 1.36 (m, 2H).

^{13}C NMR: (100 MHz, $CDCl_3$) δ 150.1, 142.7, 129.2, 124.1, 48.1, 25.7, 23.7.

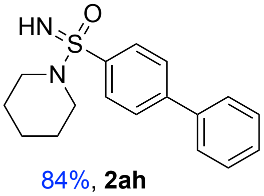

84%, **2ah**

1-([1,1'-Biphenyl]-4-sulfonimidoyl)piperidine (2ah): Synthesized according to **GP4** using 20 mg (0.035 mmol, 1.0 equiv.) of **SiNP-1h** and 1.5 equiv. of piperidine. The product was obtained as a white solid (8.9 mg, 84% yield), $R_f = 0.35$ in 30% ethyl acetate/hexane. The spectral data of the product matched with previously reported literature. [8]

^{1}H NMR: (400 MHz, $CDCl_3$) δ 7.94 (d, J = 8.6 Hz, 2H), 7.71 (d, J = 8.7 Hz, 2H), 7.63 – 7.56 (m, 2H), 7.47 (ddd, J = 7.5, 6.1, 1.3 Hz, 2H), 7.44 – 7.37 (m, 1H), 3.04 (t, J = 5.5 Hz, 4H), 1.84 (s, 1H), 1.64 (dq, J = 11.2, 5.2 Hz, 4H), 1.40 (p, J = 5.9 Hz, 2H).

^{13}C NMR: (100 MHz, $CDCl_3$) δ 145.3, 139.5, 134.6, 129.2, 128.7, 128.5, 127.5, 127.5, 48.1, 25.8, 23.7.

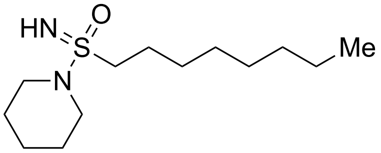

72%, **2ai**

1-(Octylsulfonimidoyl)piperidine (2ai): Synthesized according to **GP4** using 30 mg (0.056mmol, 1.0 equiv.) of **SiNP-1i** and 1.5 equiv. of piperidine. The product was obtained as a viscous liquid (10.6 mg, 72% yield), $R_f = 0.35$ in 30% ethyl acetate/hexane; iodine chamber as a stain.

^{1}H NMR: (400 MHz, $CDCl_3$) δ 3.27 (q, *J* = 5.8 Hz, 4H), 3.05 – 2.95 (m, 1H), 2.84 (ddd, *J* = 13.4, 10.4, 5.7 Hz, 1H), 1.92 – 1.79 (m, 2H), 1.67 – 1.59 (m, 4H), 1.55 (q, *J* = 5.8 Hz, 2H), 1.39 (q, *J* = 7.6 Hz, 2H), 1.36 – 1.23 (m, 8H), 0.91 – 0.84 (m, 3H).
^{13}C NMR: (100 MHz, $CDCl_3$) δ 49.2, 47.7, 31.9, 29.28, 29.1, 28.7, 26.2, 24.1, 23.6, 22.8, 14.2.
LRMS(ESI) calculated $C_{13}H_{29}N_2OS(M+H)^+$:261.2 ; found:261.5.

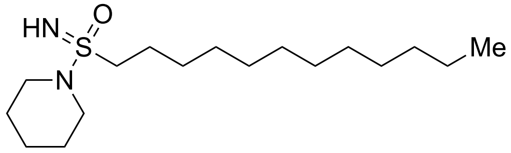

76%, **2aj**

1-(Dodecylsulfonimidoyl)piperidine (2aj): Synthesized according to **GP4** using 20 mg (0.034mmol, 1.0 equiv.) of **SiNP-1j** and 1.5 equiv. of piperidine. The product was obtained as a viscous liquid (8.2 mg, 76% yield), R_f = 0.35 in 30% ethyl acetate/hexane; iodine chamber as stain.
^{1}H NMR: (400 MHz, $CDCl_3$) δ 3.31 – 3.21 (m, 4H), 2.98 (ddd, *J* = 13.2, 10.6, 5.8 Hz, 1H), 2.82 (ddd, *J* = 13.4, 10.3, 5.9 Hz, 1H), 1.93 – 1.78 (m, 2H), 1.63 (m, 4H), 1.54 (p, *J* = 5.5 Hz, 2H), 1.38 (q, *J* = 7.5 Hz, 2H), 1.27 (d, *J* = 15.4 Hz, 16H), 0.88 (t, *J* = 7.0 Hz, 3H).
^{13}C NMR: (100 MHz, $CDCl_3$) δ 49.2, 47.7, 32.1, 29.8, 29.7, 29.5, 29.4, 29.3, 29.00, 28.7, 26.3, 24.1, 23.6, 22.8, 14.3.
LRMS(ESI) calculated $C_{17}H_{37}N_2OS(M+H)^+$:317.3 ; found:317.7.

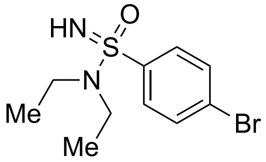

92%, **2b**

4-Bromo-*N*,*N*-diethylbenzenesulfonimidamide (2b): Synthesized according to **GP4** using 15 mg (0.026mmol, 1.0 equiv.) of **SiNP-1e** and 1.5 equiv. of diethylamine. The product was obtained as a viscous liquid (7.0 mg, 92% yield), R_f = 0.40 in 30% ethyl acetate/hexane.
^{1}H NMR: (400 MHz, $CDCl_3$) δ 7.80 (d, *J* = 8.6 Hz, 2H), 7.61 (d, *J* = 8.6 Hz, 2H), 3.29 (dq, *J* = 14.3, 7.2 Hz, 2H), 3.20 (dq, *J* = 14.2, 7.1 Hz, 2H), 2.31 (s, 1H), 1.10 (t, *J* = 7.2 Hz, 6H).
^{13}C NMR: (100 MHz, $CDCl_3$) δ 139.9, 132.2, 128.9, 126.9, 43.0, 14.6.
LRMS(ESI) calculated $C_{10}H_{16}BrN_2OS$ $(M+H)^+$: 291.0; found:291.2.

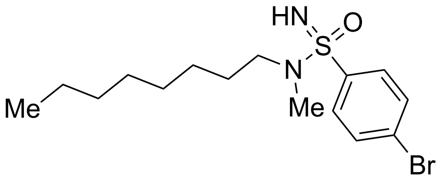

78%, **2c**

4-Bromo-*N*-methyl-*N*-octylbenzenesulfonimidamide (2c): Synthesized according to **GP5** using 24 mg (0.041mmol, 1.2 equiv.) of **SiNP-1e** and 5 mg (0.035 mmol, 1.0 equiv.) of corresponding amine. The product was obtained as a viscous liquid (9.1 mg, 78% yield), R_f = 0.40 in 30% ethyl acetate/hexane.

^{1}H NMR: (400 MHz, $CDCl_3$) δ 7.77 (d, *J* = 8.7 Hz, 2H), 7.64 (d, *J* = 8.7 Hz, 2H), 3.07 – 3.00 (m, 1H), 2.98 – 2.90 (m, 1H), 2.71 (s, 3H), 2.52 (s, 1H), 1.52 – 1.44 (m, 2H), 1.28 (t, *J* = 10.6 Hz, 10H), 0.88 (t, *J* = 7.1 Hz, 3H).
^{13}C NMR: (100 MHz, $CDCl_3$) δ 136.7, 132.2, 129.4, 127.2, 51.1, 35.7, 31.9, 29.4, 29.4, 28.1, 26.8, 22.8, 14.3.
LRMS(ESI) calculated $C_{15}H_{26}BrN_2OS$ $(M+H)^+$: 361.1; found:361.4.

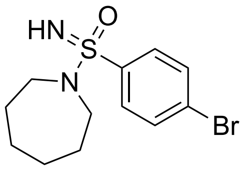

88%, **2d**

1-(4-Bromophenylsulfonimidoyl)azepane (2d): Synthesized according to **GP4** using 15 mg (0.026 mmol, 1.0 equiv.) of **SiNP-1e** and 1.5 equiv. of the corresponding amine. The product was obtained as a viscous liquid (7.3 mg, 88% yield), R_f = 0.30 in 30% ethyl acetate/hexane.
1H NMR: (400 MHz, $CDCl_3$) δ 7.78 (d, *J* = 8.7 Hz, 2H), 7.62 (d, *J* = 8.7 Hz, 2H), 3.13 (d, *J* = 4.9 Hz, 4H), 2.47 (s, 1H), 1.78 – 1.70 (m, 2H), 1.70 – 1.58 (m, 6H).
^{13}C NMR: (100 MHz, $CDCl_3$) δ 137.8, 132.1, 129.1, 126.9, 49.7, 28.3, 27.0, 25.2.
LRMS(ESI) calculated $C_{12}H_{18}BrN_2OS$ $(M+H)^+$: 317.0; found:317.2.

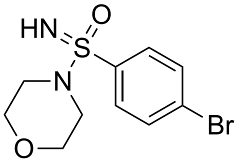

94%, **2e**

4-(4-Bromophenylsulfonimidoyl)morpholine (2e): Synthesized according to **GP4** using 20 mg (0.034mmol, 1.0 equiv) of **SiNP-1e** and 1.5 equiv. of the corresponding amine. The product was obtained as a viscous liquid (10.0 mg, 94% yield), R_f = 0.30 in 30% ethyl acetate/hexane.
^{1}H NMR: (400 MHz, $CDCl_3$) δ 7.74 (d, *J* = 8.6 Hz, 2H), 7.67 (d, *J* = 8.6 Hz, 2H), 3.74 – 3.70 (m, 4H), 3.02 – 2.95 (m, 4H).
^{13}C NMR: (100 MHz, $CDCl_3$) δ 134.1, 132.3, 129.8, 128.0, 66.5, 47.2.
LRMS(ESI) calculated $C_{10}H_{14}BrN_2O_2S$ $(M+H)^+$: 305.0; found:305.1.

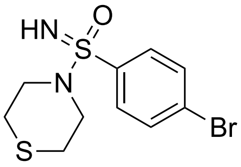

92%, **2f**

4-(4-Bromophenylsulfonimidoyl)thiomorpholine (2f): Synthesized according to **GP4** using 20 mg (0.034mmol, 1.0 equiv.) of **SiNP-1e** and 1.5 equiv. of the corresponding amine. The product was obtained as a viscous liquid (10.3 mg, 92% yield), R_f = 0.35 in 30% ethyl acetate/hexane.

^{1}**H NMR:** (400 MHz, $CDCl_3$) δ 7.72 (d, *J* = 8.6 Hz, 2H), 7.66 (d, *J* = 8.7 Hz, 2H), 3.34 (s, 4H), 2.75 – 2.65 (m, 4H), 2.03 (broad s, 1H).
^{13}**C NMR:** (100 MHz, $CDCl_3$) δ 135.7, 132.38, 129.32, 127.78, 48.95, 27.95.
LRMS(ESI) calculated $C_{10}H_{14}BrN_2OS_2$ $(M+H)^+$: 321.0; found:321.4.

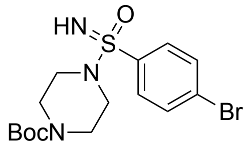

85%, **2g**

***tert*-Butyl 4-(4-bromophenylsulfonimidoyl)piperazine-1-carboxylate (2g):** Synthesized according to **GP5** using 24 mg (0.032mmol, 1.2 equiv.) of **SiNP-1e** and 5 mg **(**0.027 mmol, 1.0 equiv.) of corresponding amine. The product was obtained as a white solid (9.6 mg, 85% yield), R_f = 0.25 in 30% ethyl acetate/hexane.
^{1}**H NMR:** (400 MHz, $CDCl_3$) δ 7.73 (d, *J* = 8.5 Hz, 2H), 7.66 (d, *J* = 8.5 Hz, 2H), 3.48 (s, 4H), 2.96 (s, 4H), 1.84 (s, 1H), 1.41 (s, 9H).
^{13}**C NMR:** (100 MHz, $CDCl_3$) δ 154.28, 134.5, 132.4, 129.7, 80.5, 47.0, 28.4.
LRMS(ESI) calculated $C_{15}H_{23}BrN_3O_3S$ $(M+H)^+$: 404.1; found:404.0.

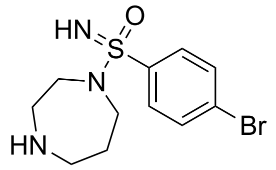

78%, **2h**

1-(4-Bromophenylsulfonimidoyl)-1,4-diazepane (2h): Synthesized according to **GP4** using 20 mg (0.034mmol, 1.0 equiv.) of **SiNP-1e** and 1.5 equiv. of the corresponding amine. The product was obtained as a viscous liquid (8.7 mg, 78% yield), R_f = 0.35 in 30% MeOH/CH_2Cl_2.
^{1}**H NMR:** (400 MHz, $CDCl_3$) δ ^{1}H NMR (500 MHz, $cdcl_3$) δ 7.78 (d, *J* = 8.7 Hz, 2H), 7.63 (d, *J* = 8.7 Hz, 2H), 3.37 (q, *J* = 5.4 Hz, 4H), 2.96 (dt, *J* = 13.8, 5.4 Hz, 4H), 1.82 (m, 3H).
^{13}**C NMR:** (100 MHz, $CDCl_3$) δ 138.7, 132.3, 128.8, 127.2, 51.7, 48.5, 47.5, 30.7, 29.8.
LRMS(ESI) calculated $C_{11}H_{17}BrN_3OS$ $(M+H)^+$: 318.1; found:318.5.

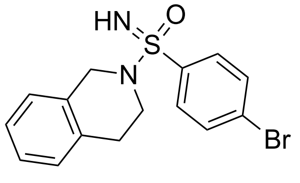

94%, **2i**

2-(4-Bromophenylsulfonimidoyl)-1,2,3,4-tetrahydroisoquinoline (2i): Synthesized according to **GP5** using 25 mg (0.045mmol, 1.2 equiv.) of **SiNP-1e** and 5 mg (0.037 mmol, 1.0 equiv.) of corresponding amine. The product was obtained as a clear oil (11.5 mg, 94% yield), R_f = 0.30 in 30% ethyl acetate/hexane.

¹H NMR: (400 MHz, $CDCl_3$) δ 7.83 (d, *J* = 8.4 Hz, 2H), 7.64 (d, *J* = 8.4 Hz, 2H), 7.16 – 7.13 (m, 2H), 7.08 (d, *J* = 4.7 Hz, 1H), 7.04 – 7.00 (m, 1H), 4.25 (s, 2H), 3.37 (t, *J* = 5.8 Hz, 2H), 2.92 (t, *J* = 5.8 Hz, 2H).
¹³C NMR: (100 MHz, $CDCl_3$) δ 135.3, 133.2, 132.2, 132.1, 129.6, 128.9, 127.6, 126.9, 126.5, 126.4, 48.9, 44.8, 29.3.
LRMS(ESI) calculated $C_{15}H_{16}BrN_2OS$ $(M+H)^+$: 351.0; found:351.4.

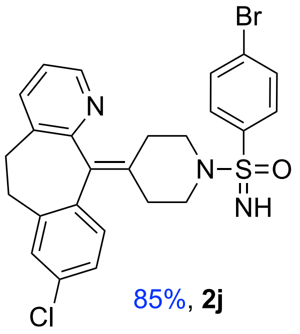

85%, **2j**

11-(1-(4-Bromophenylsulfonimidoyl)piperidin-4-ylidene)-8-chloro-6,11-dihydro-5*H*-benzo[5,6]cyclohepta[1,2-*b*]pyridine (2j): Synthesized according to **GP5** using 22 mg (0.038mmol, 1.2 equiv.) of **SiNP-1e** and 10 mg (0.032 mmol, 1.0 equiv.) of corresponding amine. The product was obtained as a clear oil (14.5 mg, 85% yield), R_f = 0.20 in 80% ethyl acetate/hexane.
¹H NMR: (400 MHz, $CDCl_3$) δ 8.36 (d, *J* = 4.6 Hz, 1H), 7.73 (d, *J* = 8.5 Hz, 2H), 7.63 (d, *J* = 8.6 Hz, 2H), 7.40 (d, *J* = 7.8 Hz, 1H), 7.15 – 7.05 (m, 3H), 7.02 (d, *J* = 8.1 Hz, 1H), 3.41 – 3.19 (m, 4H), 2.77 (ddt, *J* = 28.0, 13.7, 6.4 Hz, 4H), 2.58 (d, *J* = 5.7 Hz, 1H), 2.45 (d, *J* = 8.9 Hz, 1H), 2.40 – 2.30 (m, 2H).
¹³C NMR: (100 MHz, $CDCl_3$) δ 156.6, 146.6, 139.6, 137.8, 137.4, 136.1, 135.5, 134.6, 133.5, 133.2, 132.2, 130.5, 129.6, 129.1, 127.5, 126.3, 122.5, 48.4, 31.7, 31.5, 30.7, 30.6, 30.4, 30.4.
LRMS(ESI) calculated $C_{25}H_{24}BrClN_3OS$ $(M+H)^+$: 528.0; found:528.0.

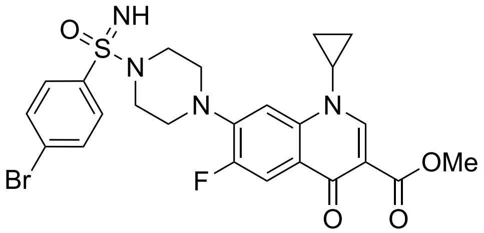

80%, **2k**

Methyl 7-(4-(4-bromophenylsulfonimidoyl)piperazin-1-yl)-1-cyclopropyl-6-fluoro-4-oxo-1,4-dihydroquinoline-3-carboxylate (2k): Synthesized according to **GP5** using 20 mg (0.034mmol, 1.2 equiv.) of **SiNP-1e** and 10 mg (0.028 mmol, 1.0 equiv.) of corresponding amine. The product was obtained as a white solid (13 mg, 80% yield), R_f = 0.20 in 20% MeOH/CH_2Cl_2.
¹H NMR: (400 MHz, $CDCl_3$) δ 8.54 (s, 1H), 8.00 (d, *J* = 13.0 Hz, 1H), 7.78 (d, *J* = 8.5 Hz, 2H), 7.69 (d, *J* = 8.5 Hz, 2H), 7.24 (d, *J* = 7.1 Hz, 1H), 3.90 (s, 3H), 3.42 (dt, *J* = 6.6, 3.1 Hz, 1H), 3.36 – 3.17 (m, 8H), 2.63 (s, 1H), 1.32 (q, *J* = 6.4 Hz, 2H), 1.13 (t, *J* = 4.6 Hz, 2H).
¹³C NMR: (100 MHz, $CDCl_3$) δ 173.1, 166.5, 154.6, 152.2, 148.7, 143.9 (d, *J* = 10.8 Hz), 138.0, 134.4, 132.4, 129.7, 128.1, 123.9 (d, *J* = 6.9 Hz), 113.7 (d, *J* = 23.0 Hz), 110.3, 105.4 (d, *J* = 2.8 Hz), 52.3, 49.9, 49.8, 47.0, 34.6, 8.3.

^{19}F NMR: (100 MHz, $CDCl_3$) -123.92.
LRMS(ESI) calculated $C_{24}H_{25}BrFN_4O_4S$ $(M+H)^+$: 563.1; found:563.3.

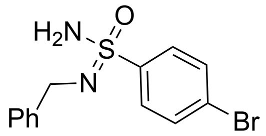

72%, **2l**

***N*'-Benzyl-4-bromobenzenesulfonimidamide (2l):** Synthesized according to **GP5** using 32 mg (0.056mmol, 1.2 equiv.) of **SiNP-1e** and 5 mg (0.046 mmol, 1.0 equiv.) of corresponding amine. The product was obtained as a clear oil (11 mg, 72% yield), R_f = 0.25 in 30% ethyl acetate/hexane.
^{1}H NMR: (400 MHz, $CDCl_3$) δ 7.84 (d, *J* = 8.6 Hz, 2H), 7.62 (d, *J* = 8.7 Hz, 2H), 7.28 (d, *J* = 7.4 Hz, 3H), 7.20 – 7.15 (m, 2H), 4.15 (d, *J* = 13.8 Hz, 1H), 4.07 (d, *J* = 14.0 Hz, 1H).
^{13}C NMR: (100 MHz, $CDCl_3$) δ 140.0, 136.6, 132.4, 129.1, 128.9, 128.1, 128.0, 127.4, 48.1.
LRMS(ESI) calculated $C_{13}H_{14}BrN_2OS$ $(M+H)^+$: 325.0; found:325.2.

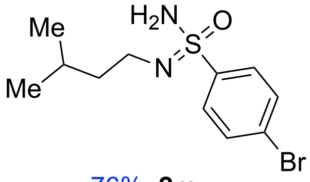

76%, **2m**

4-Bromo-*N*'-isopentylbenzenesulfonimidamide (2m): Synthesized according to **GP5** using 40 mg (0.048mmol, 1.2 equiv.) of **SiNP-1e** and 5 mg (0.057 mmol, 1.0 equiv.) of corresponding amine. The product was obtained as a clear oil (13.3 mg, 76% yield), R_f = 0.30 in 30% ethyl acetate/hexane.
^{1}H NMR: (400 MHz, $CDCl_3$) δ 7.85 (d, *J* = 8.7 Hz, 2H), 7.64 (d, *J* = 8.7 Hz, 2H), 3.13 (s, 2H), 2.97 – 2.88 (m, 2H), 1.56 (dq, *J* = 13.4, 6.7 Hz, 1H), 1.31 (q, *J* = 7.1 Hz, 2H), 0.84 (dd, *J* = 6.5, 4.8 Hz, 6H).
^{13}C NMR: (100 MHz, $CDCl_3$) δ 140.1, 132.3, 129.1, 127.3, 42.2, 38.6, 25.6, 22.4, 22.4.
LRMS(ESI) calculated $C_{11}H_{18}BrN_2OS$ $(M+H)^+$: 305.0; found:305.1.

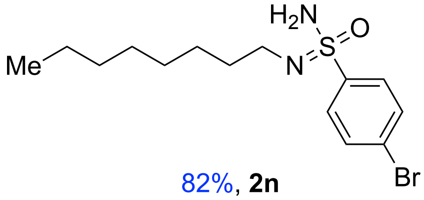

82%, **2n**

4-Bromo-*N*'-octylbenzenesulfonimidamide (2n): Synthesized according to **GP5** using 26 mg (0.048mmol, 1.2 equiv.) of **SiNP-1e** and 5 mg (0.038 mmol, 1.0 equiv.) of corresponding amine. The product was obtained as a clear oil (11 mg, 82% yield), R_f = 0.35 in 30% ethyl acetate/hexane.
^{1}H NMR: (400 MHz, $CDCl_3$) δ 7.84 (d, *J* = 8.5 Hz, 2H), 7.63 (d, *J* = 8.5 Hz, 2H), 3.10 (s, 2H), 2.94 – 2.87 (m, 2H), 1.41 (q, *J* = 6.9 Hz, 2H), 1.30 – 1.20 (m, 10H), 0.87 (t, *J* = 7.0 Hz, 3H).
^{13}C NMR: (100 MHz, $CDCl_3$) δ 140.2, 132.3, 129.1, 127.3, 43.9, 31.9, 29.7, 29.3, 29.2, 26.7, 22.76, 14.23.
LRMS(ESI) calculated $C_{14}H_{24}BrN_2OS$ $(M+H)^+$: 347.1; found:347.3.

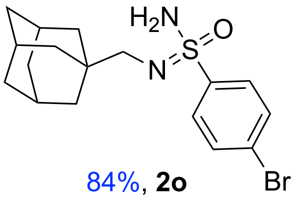

84%, **2o**

***N'*-(((3*r*,5*r*,7*r*)-Adamantan-1-yl)methyl)-4-bromobenzenesulfonimidamide (2o):** Synthesized according to **GP5** using 21 mg (0.036mmol, 1.2 equiv) of **SiNP-1e** and 5 mg (0.030 mmol, 1.0 equiv.) of corresponding amine. The product was obtained as a clear oil (9.7 mg, 84% yield), R_f = 0.30 in 30% ethyl acetate/hexane.

^{1}H NMR: (400 MHz, $CDCl_3$) δ 7.83 (d, *J* = 8.7 Hz, 2H), 7.63 (d, *J* = 8.7 Hz, 2H), 2.67 (s, 2H), 2.58 – 2.51 (m, 2H), 1.97 (s, 3H), 1.70 (d, *J* = 12.2 Hz, 3H), 1.59 (d, *J* = 11.4 Hz, 3H), 1.43 (s, 6H).

^{13}C NMR: (100 MHz, $CDCl_3$) δ 140.3, 132.3, 129.0, 128.2, 55.6, 40.2, 36.9, 33.2, 28.2.

LRMS(ESI) calculated $C_{17}H_{24}BrN_2OS$ $(M+H)^+$: 383.1; found:383.2.

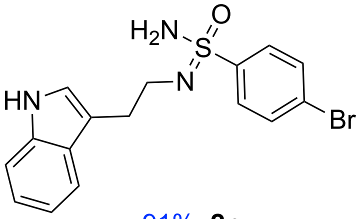

91%, **2p**

***N'*-(2-(1*H*-Indol-3-yl)ethyl)-4-bromobenzenesulfonimidamide (2p):** Synthesized according to **GP5** using 22 mg (0.039 mmol, 1.2 equiv) of **SiNP-1e** and 5 mg (0.031 mmol, 1.0 equiv.) of corresponding amine. The product was obtained as a viscous liquid (10.7 mg, 91% yield), R_f = 0.15 in 40% ethyl acetate/hexane.

^{1}H NMR: (400 MHz, $CDCl_3$) δ 8.03 (s, 1H), 7.67 (d, *J* = 8.6 Hz, 2H), 7.49 (d, *J* = 8.5 Hz, 2H), 7.37 (dd, *J* = 11.0, 8.1 Hz, 2H), 7.20 (t, *J* = 7.4 Hz, 1H), 7.07 (t, *J* = 7.5 Hz, 1H), 6.97 (s, 1H), 3.27 (dp, *J* = 19.0, 6.3 Hz, 2H), 3.24 (broad singlet, 2H), 2.92 (t, *J* = 6.5 Hz, 2H).

^{13}C NMR: (100 MHz, $CDCl_3$) δ 139.9, 136.5, 132.2, 128.9, 127.2, 126.9, 122.6, 119.8, 118.6, 111.8, 111.5, 43.7, 25.6.

LRMS(ESI) calculated $C_{16}H_{17}BrN_3OS$ $(M+H)^+$: 378.0; found:378.1.

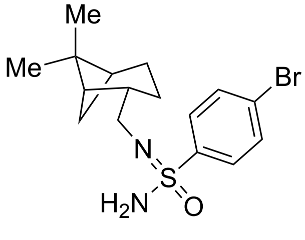

88%, **2q**

1:1 mixture of diastereomers

4-Bromo-*N'*-((6,6-dimethylbicyclo[3.1.1]heptan-2-yl)methyl)benzenesulfonimidamide (2q): Synthesized according to **GP5** using 22 mg (0.039 mmol, 1.2 equiv) of **SiNP-1e** and 5 mg (0.033 mmol, 1.0 equiv.) of corresponding amine. The product was obtained as a clear oil (10.6 mg, 88% yield), R_f = 0.35 in 30% ethyl acetate/hexane.

^{1}H NMR: (400 MHz, CDCl3) δ 7.86 (d, *J* = 8.6 Hz, 4H), 7.65 (d, *J* = 8.6 Hz, 4H), 3.65 (s, 4H), 2.97 – 2.84 (m, 4H), 2.38 – 2.30 (m, 2H), 2.16 – 2.05 (m, 2H), 1.95 – 1.77 (m, 10H), 1.45 – 1.29 (m, 2H), 1.15 (s, 3H), 1.12 (s, 3H), 0.89 (s, 3H), 0.88 (s, 3H), 0.85 (dd, *J* = 9.7, 4.9 Hz, 2H).
^{13}C NMR: (100 MHz, CDCl3) δ 139.8, 132.4, 129.2, 129.1, 127.6, 127.6, 49.3, 49.2, 43.5, 43.5, 41.3, 41.2, 38.7, 38.6, 33.2, 33.1, 27.9, 26.0, 25.9, 23.3, 23.2, 19.9, 19.8.
LRMS(ESI) calculated $C_{16}H_{24}BrN_2OS$ $(M+H)^+$: 371.1; found:371.4.

4. Scale-up Reaction ans Synthetic Applications

4.1 Reaction scale-up

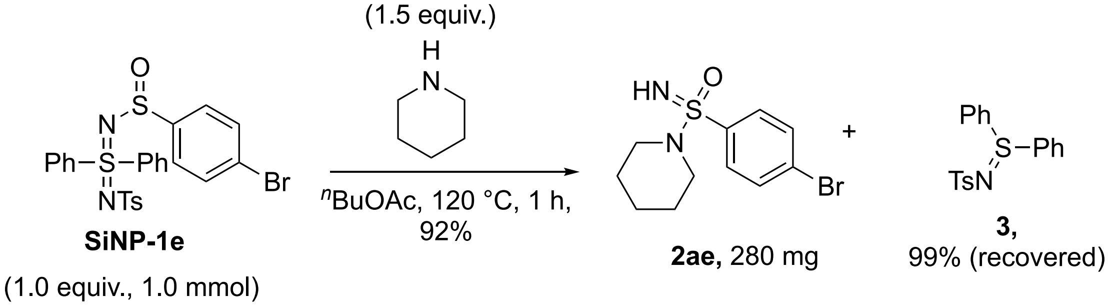

To a solution of (1.5 equiv. of piperidine) in 0.1 M *n*-butyl acetate in a two-neck round-bottom flask in a pre-heated heating mantle at 120 °C, **SiNP-1e** (1.0 equiv, 1.0 mmol, 0.572 g) was added portionwise manner (3 portions) and heated for 1 hour after the last portion until the reaction was complete. After the reaction, all volatiles were removed under reduced pressure, and the crude mixture was purified by silica gel column chromatography.
1-(4-Bromophenylsulfonimidoyl)piperidine (2ae):
1H NMR: (400 MHz, CDCl3) δ 7.75 (d, *J* = 8.8 Hz, 2H), 7.64 (d, *J* = 8.7 Hz, 2H), 2.99 (t, *J* = 5.6 Hz, 4H), 1.88 (s, 1H), 1.62 (dq, *J* = 11.1, 5.3 Hz, 4H), 1.40 (td, *J* = 7.3, 4.3 Hz, 2H).
13C NMR: (100 MHz, CDCl3) δ 135.33, 132.09, 129.68, 127.37, 48.07, 25.72, 23.69.

***N*-(Diphenyl-λ4-sulfaneylidene)-4-methylbenzenesulfonamide (3):** The compound was recovered with silica-gel column chromatography. The product was obtained as a white solid (355 mg, 99% yield), R_f = 0.2 in 40% ethyl acetate/hexane.
^{1}H NMR: (400 MHz, CDCl3) δ 7.74 (d, *J* = 7.8 Hz, 2H), 7.62 (d, *J* = 7.7 Hz, 4H), 7.47 (dt, *J* = 14.9, 7.2 Hz, 6H), 7.14 (d, *J* = 7.9 Hz, 2H), 2.34 (s, 3H).
^{13}C NMR: (100 MHz, CDCl3) δ 141.8, 141.4, 136.6, 132.4, 130.0, 129.3, 127.4, 126.4, 21.5.

4.2 Flowchart for the recycling of the by-product

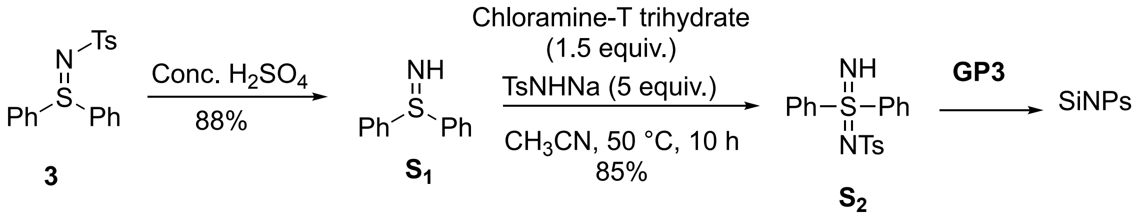

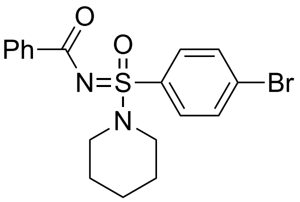

86%, **3a**

***N*-((4-Bromophenyl)(oxo)(piperidin-1-yl)-λ6-sulfaneylidene)benzamide (3a):** To a solution of **2af** (20 mg) in 0.1 M tetrahydrofuran at ice cold conditions, 1.5 equiv. NaH (60%) was added and stirred for the next 30 min. Next, 1.2 equiv. benzoyl chloride was added at room temperature and stirred overnight until the reaction was complete. The product was obtained as a white solid (23 mg, 86% yield), R_f = 0.40 in 20% ethyl acetate/hexane.
^{1}H NMR: (400 MHz, $CDCl_3$) δ 8.15 (d, *J* = 7.5 Hz, 2H), 7.82 (d, *J* = 8.4 Hz, 2H), 7.69 (d, *J* = 8.4 Hz, 2H), 7.50 (q, *J* = 7.7 Hz, 1H), 7.41 (t, *J* = 7.5 Hz, 2H), 3.23 (hept, *J* = 5.9 Hz, 4H), 1.74 – 1.64 (m, 4H), 1.57 – 1.49 (m, 2H).
^{13}C NMR: (100 MHz, $CDCl_3$) δ 172.8, 136.0, 135.9, 132.6, 132.3, 130.3, 129.6, 129.4, 128.2, 46.6, 25.4, 23.7.

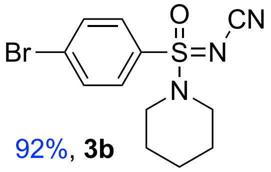

92%, **3b**

***N*-((4-Bromophenyl)(oxo)(piperidin-1-yl)-λ6-sulfaneylidene)cyanamide (3b):** To a solution of **2af** (20 mg) in 0.1 M tetrahydrofuran at ice cold conditions, 1.5 equiv. NaH (60%) was added and stirred for the next 30 min. Next, 1.2 equiv. cyanogen bromide was added at room temperature and stirred overnight until the reaction was complete. The product was obtained as a white solid (20.30 mg, 92% yield), R_f = 0.25 in 20% ethyl acetate/hexane.
^{1}H NMR: (400 MHz, $CDCl_3$) δ 7.76 – 7.69 (m, 4H), 3.15 (t, *J* = 5.5 Hz, 4H), 1.79 – 1.67 (m, 4H), 1.52 (q, *J* = 6.0 Hz, 2H).
^{13}C NMR: (100 MHz, $CDCl_3$) δ 133.1, 132.7, 129.9, 129.3, 110.8, 47.5, 25.1, 23.4.
LRMS(ESI) calculated $C_{12}H_{15}BrN_2OS$ $(M+H)^+$: 328.0; found:328.3.

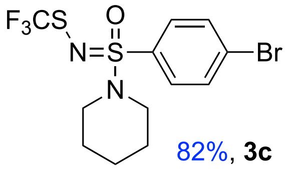

82%, **3c**

***N*-((4-Bromophenyl)(oxo)(piperidin-1-yl)-λ6-sulfaneylidene)-*S*-(trifluoromethyl) thiohydroxylamine (3c):** To a solution of **2af** (20 mg) in 0.1 M acetonitrile at room temperature, 1.05 equiv. of NBS was added and stirred for the next 30 min. Next, 1.2 equiv. $AgSCF_3$ was added at room temperature to the resulting mixture, and the mixture was stirred for another 30 min until the reaction was complete. All the volatiles were removed and purified by column chromatography. The product was obtained as a white solid (24.0 mg, 82% yield), R_f = 0.35 in 20% ethyl acetate/hexane.

^{1}H NMR: (400 MHz, $CDCl_3$) δ 7.79 – 7.63 (m, 4H), 3.02 (dq, J = 17.1, 5.8 Hz, 4H), 1.67 – 1.63 (m, 4H), 1.46 (p, J = 5.9 Hz, 2H).
^{13}C NMR: (100 MHz, $CDCl_3$) δ 134.3, 132.5, 129.5, 128.5, 47.6, 25.2, 23.6.
^{19}F NMR: (376 MHz, $CDCl_3$), δ -50.4.
LRMS(ESI) calculated $C_{12}H_{15}BrF_3N_2OS_2$ $(M+H)^+$: 403.0; found:403.1.

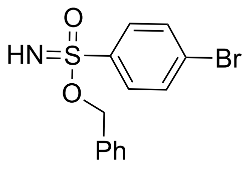

2r, 58%

Benzyl 4-Bromobenzenesulfonimidate (2r): Synthesized according to **GP4** using 15 mg (0.026 mmol, 1.0 equiv.) of **SiNP-1e** and 1.5 equiv. of the benzyl alcohol. The product can be tracked using crude 1H NMR but attempts at purification with silica gel column chromatography or neutral alumina were unsuccessful. The yield reported is the NMR yield.

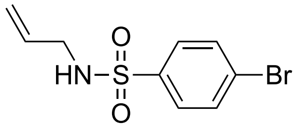

2s, 34%

***N*-Allyl-4-bromobenzenesulfonamide (2s):** Synthesized according to **GP4** using 30 mg (0.026 mmol, 1.0 equiv.) of **SiNP-1e** and 1.0 equiv. of the allyl alcohol. All the volatiles were removed under reduced pressure and purified by column chromatography. The spectral data matched the reported literature.[12]

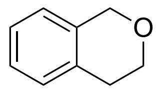

2t, 52%

Isochromane (2t): Synthesized according to **GP5** using 20 mg (0.026 mmol, 1.0 equiv.) of diol and 1.5 equiv. of **SiNP-1e**. All the volatiles were removed under reduced pressure and purified by column chromatography. The spectral data matched the reported literature.[13]

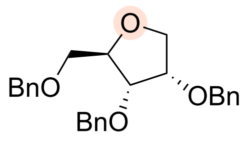

2u, 55%

(2*R*,3*S*,4*S*)-3,4-Bis(benzyloxy)-2-((benzyloxy)methyl)tetrahydrofuran (2u): Synthesized according to **GP5** using 13 mg (0.030 mmol, 1.0 equiv.) of diol and 1.5 equiv. of **SiNP-1e**. All the volatiles were removed under reduced pressure and purified by column chromatography. The product was obtained as a clear oil (6.8 mg, 55% yield), R_f = 0.35 in 15% ethyl acetate/hexane, and the spectral data matched the reported literature.[14]

¹H NMR: (400 MHz, $CDCl_3$) δ 7.32 (ddd, *J* = 17.2, 9.2, 4.8 Hz, 15H), 4.66 – 4.54 (m, 4H), 4.50 (dd, *J* = 12.0, 6.3 Hz, 2H), 4.16 (dt, *J* = 7.0, 4.0 Hz, 1H), 4.01 (ddd, *J* = 17.1, 9.2, 4.6 Hz, 2H), 3.97 – 3.91 (m, 2H), 3.62 (dd, *J* = 10.6, 3.2 Hz, 1H), 3.52 (dd, *J* = 10.6, 4.3 Hz, 1H).
¹³C NMR: (100 MHz, $CDCl_3$) δ 138.3, 138.1, 138.0, 128.6, 128.52, 128.48, 128.12, 128.05, 127.9, 127.9, 127.8, 127.7, 80.6, 78.4, 76.6, 73.6, 72.3, 71.9, 70.8, 70.1.

4.3 Mechanism for the deoxygenation by sulfinylnitrene

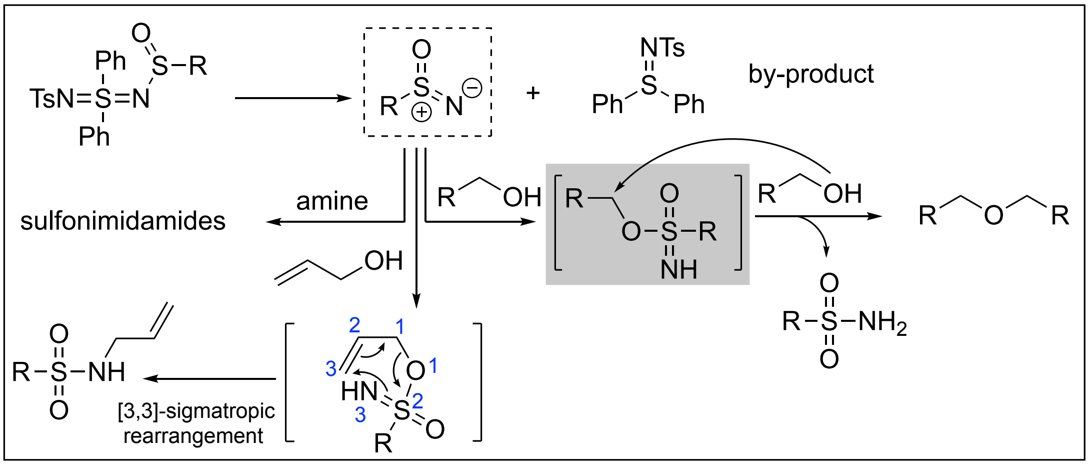

4.4 Limitations of the methodology

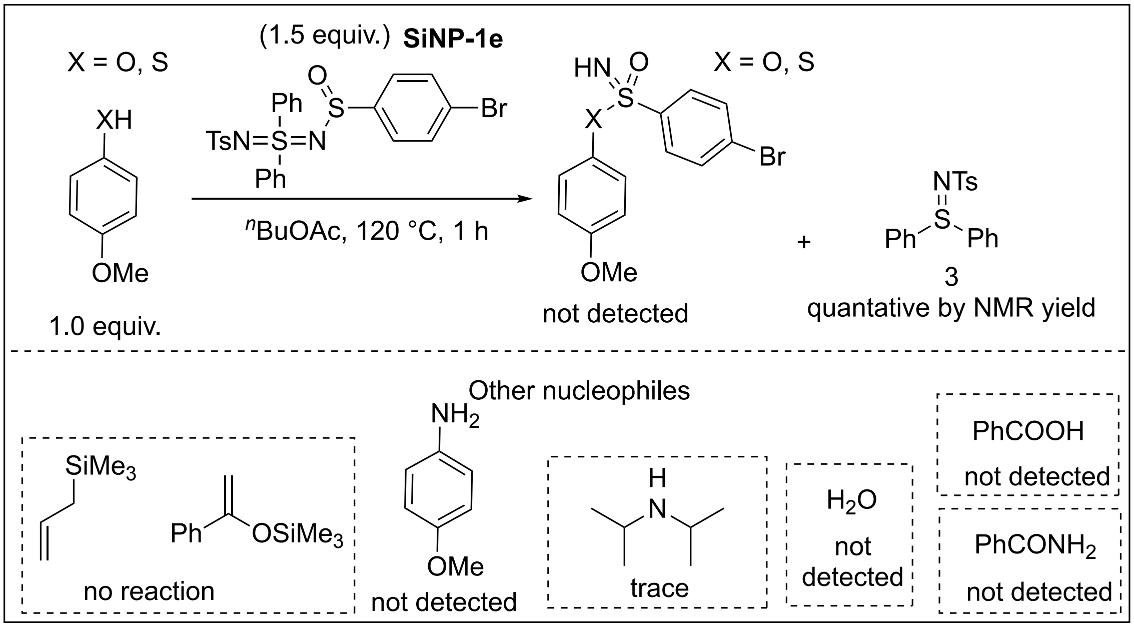

5. Data for Single Crystal X-ray Diffraction

5.1 Crystal data for compound SiNP-1c

The data collection was carried out using Mo Kα radiation (graphite monochromator) with a frame time of 5 and 40 seconds and a detector distance of 6.0 cm. A collection strategy was calculated and complete data to a resolution of 0.77 Å with a redundancy of 9.01 were collected. Seven major sections of frames were collected with 1º ϕ and ω scans. A total of 1390 frames were collected. The total exposure time was 10.64 hours. The frames were integrated with the SAINT V8.41 package using a narrow-frame algorithm. The integration of the data using a monoclinic unit cell yielded a total of 48303 reflections to a maximum θ angle of 27.47° (0.77 Å resolution), of which 5289 were independent (average redundancy 9.13, completeness = 100.0%, R_{int} = 4.91%, R_{sig} = 2.46%) and 4876 (92.2%) were greater than 2σ(*F*). The final cell constants of a = 8.2569(5) Å, b = 18.2568(11) Å, c = 15.3551(10) Å, β = 92.303(2)°, volume = 2312.8(2) Å, are based upon the refinement of the XYZ-centroids of 9749 reflections above 20 σ(*I*) with 2.60° < 2θ < 27.85°. Data were corrected for absorption effects using the Multi-Scan method in SADABS 2016/2. The calculated minimum and maximum transmission coefficients (based on crystal size) are 0.607 and 0.746.

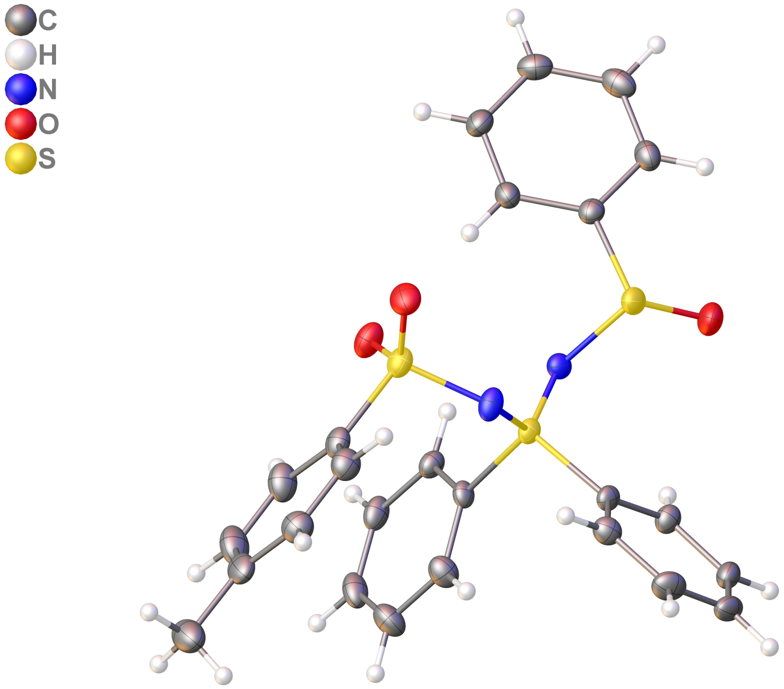

Formula unit.

Thermal ellipsoids were drawn at 50% probability level.

Empirical formula	C25 H22 N2 O3 S3	
Formula weight	494.62	
Crystal color, shape, size	colourless plate, 0.263 × 0.222 × 0.052 mm^3	
Temperature	100(2) K	
Wavelength	0.71073 Å	
Crystal system, space group	Monoclinic, $P2_1/n$	
Unit cell dimensions	a = 8.2569(5) Å	α = 90°
	b = 18.2568(11) Å	β = 92.303(2)°
	c = 15.3551(10) Å	γ = 90°
Volume	2312.8(2) $Å^3$	
Z	4	
Density (calculated)	1.421 Mg/m^3	
Absorption coefficient	0.352 mm^{-1}	
F(000)	1032	
Data collection		
Diffractometer	D8 QUEST, Bruker	
Source	IμS 2.0, Incoatec	
Detector	PHOTON II	
Theta range for data collection	2.231 to 27.472°	
Index ranges	-7<=h<=10, -23<=k<=23, -19<=l<=19	
Reflections collected	48303	
Independent reflections	5289 [R_{int} = 0.0491]	
Observed Reflections	4876	
Completeness to theta = 25.242°	100.0 %	
Solution and Refinement		
Absorption correction	Multi-Scan	
Max. and min. transmission	0.7456 and 0.6068	
Solution	Intrinsic methods	
Refinement method	Full-matrix least-squares on F^2	
Weighting scheme	$w = [\sigma^2Fo^2 + AP^2 + BP]^{-1}$, with	
	$P = (Fo^2 + 2 Fc^2)/3$, A = 0.0296, B = 3.2832	
Data / restraints / parameters	5289 / 0 / 299	
Goodness-of-fit on F^2	1.179	
Final R indices [$I>2\sigma(I)$]	R1 = 0.0473, wR2 = 0.1090	
R indices (all data)	R1 = 0.0516, wR2 = 0.1112	
Extinction coefficient	n/a	
Largest diff. peak and hole	0.790 and -0.448 e.$Å^{-3}$	

5.2 Crystal data for Compound 3c

The data collection was carried out using Mo Kα radiation (graphite monochromator) with a frame time of 2 and 15 seconds and a detector distance of 6.0 cm. A collection strategy was calculated and complete data to a resolution of 0.76 Å with a redundancy of 7.45 were collected. Six major sections of frames were collected with 1° ϕ and ω scans. A total of 1256 frames were collected. The total exposure time was 3.45 hours. The frames were integrated with the SAINT V8.41 package using a narrow-frame algorithm. The integration of the data using a monoclinic unit cell yielded a total of 14758 reflections to a maximum θ angle of 28.32° (0.75 Å resolution), of which 3732 were independent (average redundancy 3.95, completeness = 100.0%, R_{int} = 3.42%, R_{sig} = 3.19%) and 3547 (95.0%) were greater than 2σ(*F*). The final cell constants of a = 17.7414(6) Å, b = 10.0068(4) Å, c = 9.1503(4) Å, β = 103.9190(10)°, volume = 1576.79(11) $Å^3$, are based upon the refinement of the XYZ-centroids of 9974 reflections above 20 σ(*I*) with 2.35° < 2θ < 28.23°. Data were corrected for absorption effects using the Multi-Scan method in SADABS 2016/2. The calculated minimum and maximum transmission coefficients (based on crystal size) are 0.566 and 0.746.

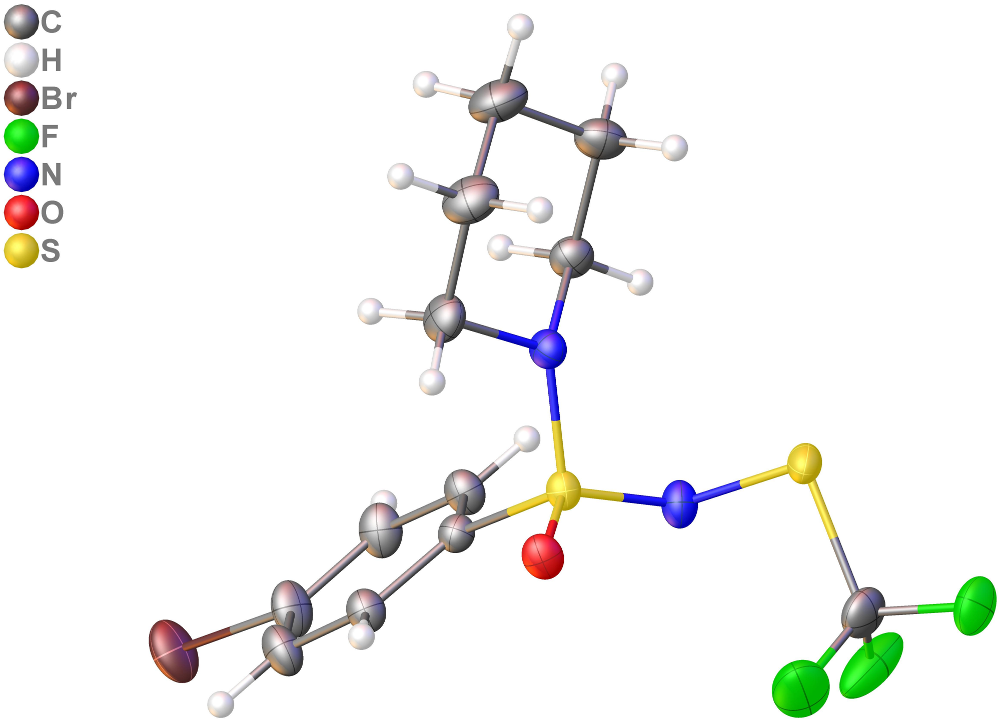

Formula unit.

Disorders were omitted for clarity.

Thermal ellipsoids were drawn at 50% probability level.

Empirical formula	C12 H14 Br F3 N2 O S2	
Formula weight	403.28	
Crystal color, shape, size	colourless plate, 0.191 × 0.174 × 0.052 mm^3	
Temperature	100(2) K	
Wavelength	0.71073 Å	
Crystal system, space group	Monoclinic, *C*2	
Unit cell dimensions	a = 17.7414(6) Å	α = 90°
	b = 10.0068(4) Å	β = 103.9190(10)°
	c = 9.1503(4) Å	γ = 90°
Volume	1576.79(11) $Å^3$	
Z	4	
Density (calculated)	1.699 Mg/m^3	
Absorption coefficient	2.901 mm^{-1}	
F(000)	808	
Data collection		
Diffractometer	D8 QUEST, Bruker	
Source	IμS 2.0, Incoatec	
Detector	PHOTON II	
Theta range for data collection	2.293 to 27.875°	
Index ranges	-21<=h<=23, -13<=k<=13, -12<=l<=12	
Reflections collected	14758	
Independent reflections	3732 [R_{int} = 0.0342]	
Observed Reflections	3547	
Completeness to theta = 25.242°	100.0 %	
Solution and Refinement		
Absorption correction	Multi-Scan	
Max. and min. transmission	0.7457 and 0.5663	
Solution	Intrinsic methods	
Refinement method	Full-matrix least-squares on F^2	
Weighting scheme	$w = [\sigma^2Fo^2+ AP^2+ BP]^{-1}$, with	
	$P = (Fo^2+ 2 Fc^2)/3$, A = 0.0290, B = 0.5462	
Data / restraints / parameters	3732 / 273 / 229	
Goodness-of-fit on F^2	1.044	
Final R indices [I>2σ(I)]	R1 = 0.0244, wR2 = 0.0594	
R indices (all data)	R1 = 0.0263, wR2 = 0.0603	
Absolute structure parameter	0.319(8)	
Extinction coefficient	n/a	
Largest diff. peak and hole	0.449 and -0.337 e.$Å^{-3}$	
Twin details		
Type	Inversion	
Domain ratio	68:32	

6. DFT Calculation on the Bond Length

Density functional theory (DFT) was employed to predict the structure of singlet *p*-toluyl sulfinylnitrene. Geometry optimization was performed using B3LYP-D3BJ/6-31G*//*vacuuo* as implemented in *QChem* 5.4.[15] The optimized structure was confirmed as a potential energy surface minimum using vibrational analysis at the same level of theory: the structure has no imaginary vibrational modes. Relevant bond lengths are in agreement with previously published results.[7] Other computational details follow.

Bond	Length
S-N	1.47 Å
S-C	1.80 Å
S=O	1.48 Å

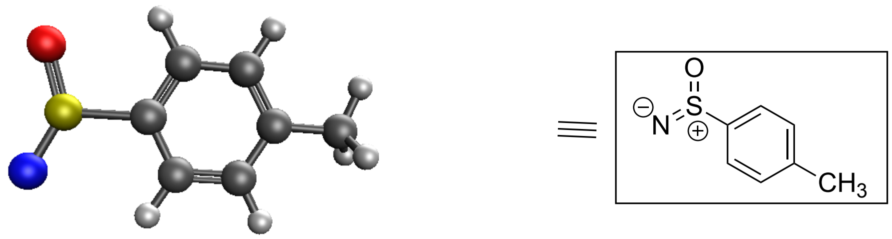

A. Absolute electronic energy of optimized geometry: -799.033991 Eh

B. Coordinates of optimized geometry

0 1

C	1.6756613	-1.2008195	0.0015608
C	2.3959388	0.0001363	-0.0000656
C	0.2823566	-1.2074908	0.0017500
C	-0.3871666	0.0151581	0.0004600
C	0.2978255	1.2324834	-0.0010935
C	1.6870638	1.2125589	-0.0014404
C	3.9035430	0.0006200	-0.0002209
S	-2.1830087	0.0547480	-0.0001031
O	-2.6404325	-1.3531614	-0.0034910
H	2.2125469	-2.1452935	0.0035426
H	-0.2802316	-2.1333658	0.0031330
H	-0.2592093	2.1630453	-0.0025924
H	2.2342502	2.1515639	-0.0038402
H	4.3038274	-1.0168231	-0.0001978
H	4.2974540	0.5196842	0.8816072
H	4.2970419	0.5199867	-0.8820484
N	-2.8407736	1.3678069	0.0034672

C. Hessian vibrational analysis

Frequency:	53.54	65.10	98.64
Frequency:	170.46	243.76	293.26
Frequency:	321.87	342.54	408.01
Frequency:	415.05	484.17	500.28
Frequency:	630.03	646.82	701.99
Frequency:	814.78	824.75	853.26
Frequency:	976.14	981.53	1015.72
Frequency:	1029.41	1061.09	1076.05

Frequency:	1099.41	1147.49	1215.13
Frequency:	1243.53	1337.67	1362.55
Frequency:	1406.90	1441.87	1449.17
Frequency:	1513.52	1521.68	1534.94
Frequency:	1632.14	1652.19	3052.25
Frequency:	3106.42	3140.53	3193.12
Frequency:	3195.87	3230.44	3244.83

This molecule has 0 imaginary frequencies

7. References

[1] P. Xie, Y. Zheng, Y. Luo, J. Luo, L. Wu, Z. Cai, L. He, *Org. Lett.* **2023**, *25*, 6133-6138.

[2] A. V. Mackenroth, P. W. Antoni, F. Rominger, M. Rudolph, A. S. K. Hashmi, *Org. Lett.* **2023**, *25*, 2907–2912.

[3] B. Ghosh, P. Kafle, R. Mukherjee, R. Welles, D. Herndon, K. M. Nicholas, Y. Shao, I. Sharma, *Science* **2025**, *387*, 102–107.

[4] Z. Zhong, T.-K. Ma, A. J. P. White, J. A. Bull, *Org. Lett.* **2024**, *26*, 1178–1183.

[5] X. Chen, C. Bour, E. Kolodziej, R. Guillot, N. S. George, A. Alix, V. Gandon, *Adv. Synth. Catal.* **2025**, *367*, e202401085.

[6] T. J. Maricich, C. N. Angeletakis, R. Mjanger, *J. Org. Chem.* **1984**, *49*, 1928-1931.

[7] T. Q. Davies, M. J. Tilby, J. Ren, N. A. Parker, D. Skolc, A. Hall, F. Duarte, M. C. Willis, *J. Am. Chem. Soc.* **2020**, *142*, 15445-15453.

[8] E. L. Briggs, A. Tota, M. Colella, L. Degennaro, R. Luisi, J. A. Bull, *Angew. Chem. Int. Edt.* **2019**, *58*, 14303-14310.

[9] D.-B. Zeng, B. Zhao, C.-X. Gong, Y. Lin, K.-Y. Ye, *Org. Lett.* **2025**, *27*, 9316-9321.

[10] P. Wu, J. Demaerel, D. Kong, D. Ma, C. Bolm, *Org. Lett.* **2022**, *24*, 6988-6992.

[11] F. Izzo, M. Schäfer, R. Stockman, U. Lücking, *Chem. Eur. J.* **2017**, *23*, 15189-15193.

[12] T. Go, A. Morimatsu, H. Wasada, G. Tanabe, O. Muraoka, Y. Sawada, M. Yoshimatsu, *Beilstein J. Org. Chem.* **2018**, *14*, 2722-2729.

[13] Z.-H. Zhang, R. Sun, Y.-Y. Jiang, J.-Q. Lin, Y.-Q. Zhang, B.-H. Xu, *J. Catal.* **2025**, *445*, 116034.

[14] N. Hossain, H. van Halbeek, E. De Clercq, P. Herdewijn, *Tetrahedron* **1998**, *54*, 2209-2226.

[15] Y. Shao, Z. Gan, E. Epifanovsky, A. T. Gilbert, M. Wormit, J. Kussmann, A. W. Lange, A. Behn, J. Deng, X. Feng, *Mol. Phys.* **2015**, *113*, 184-215.

8. References

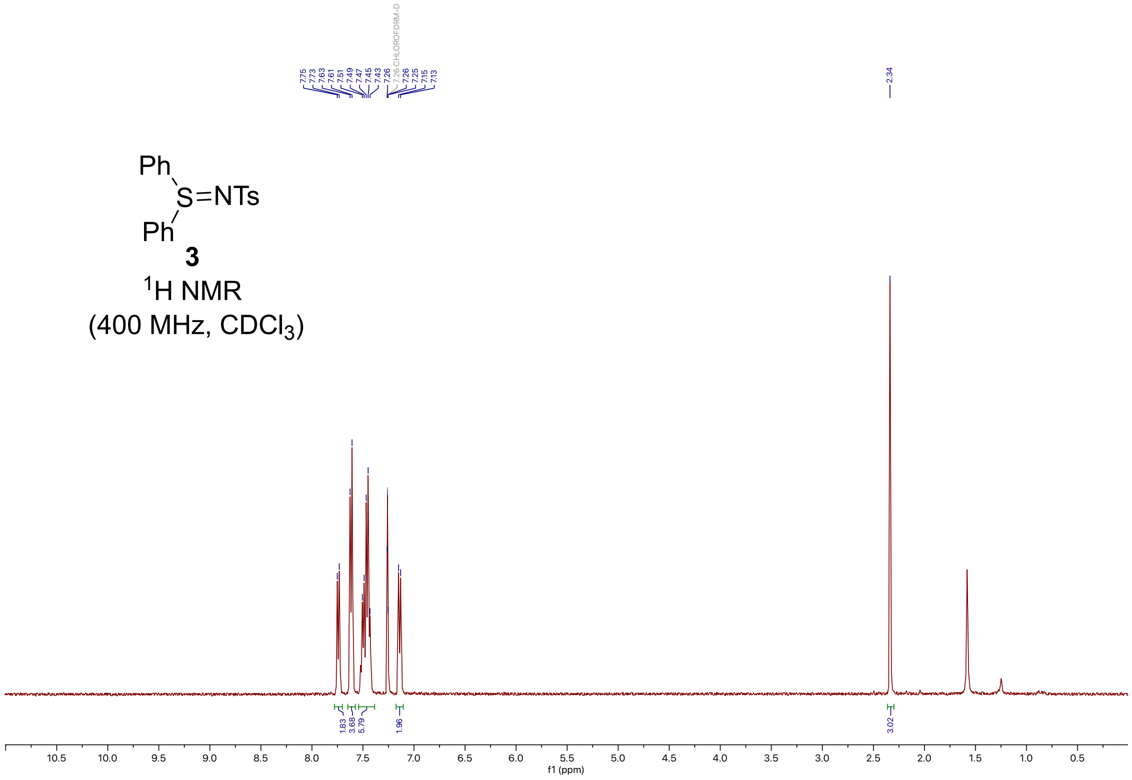

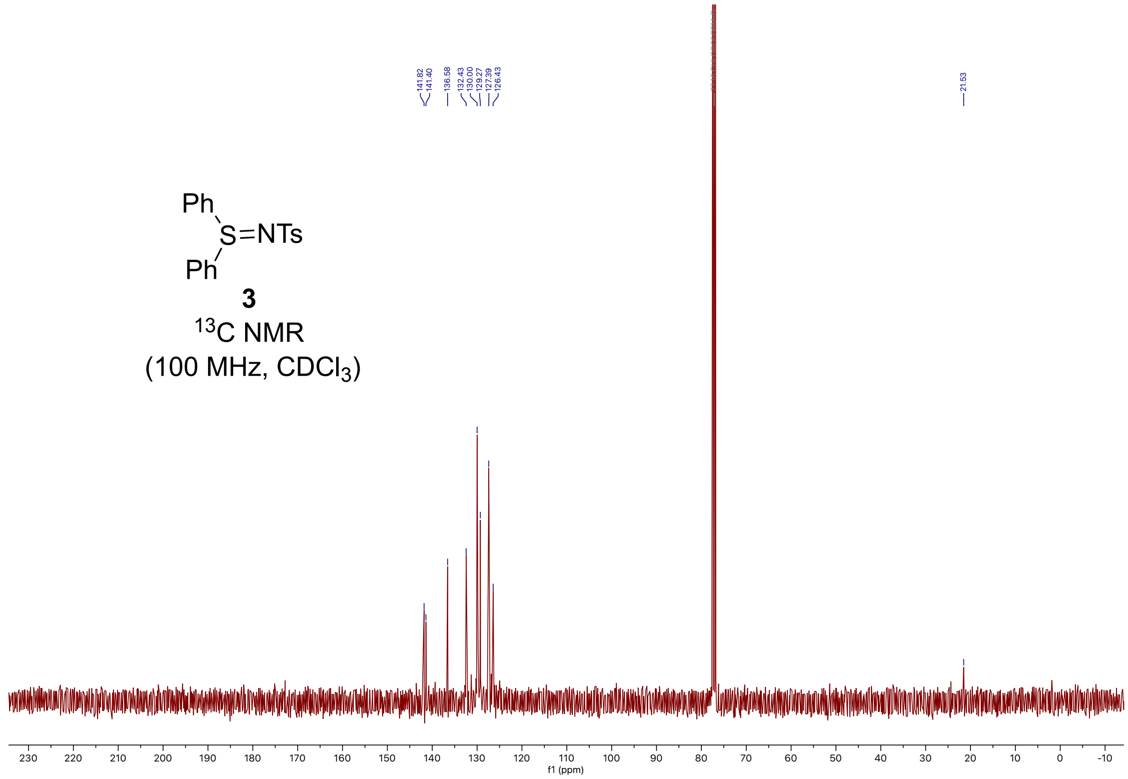

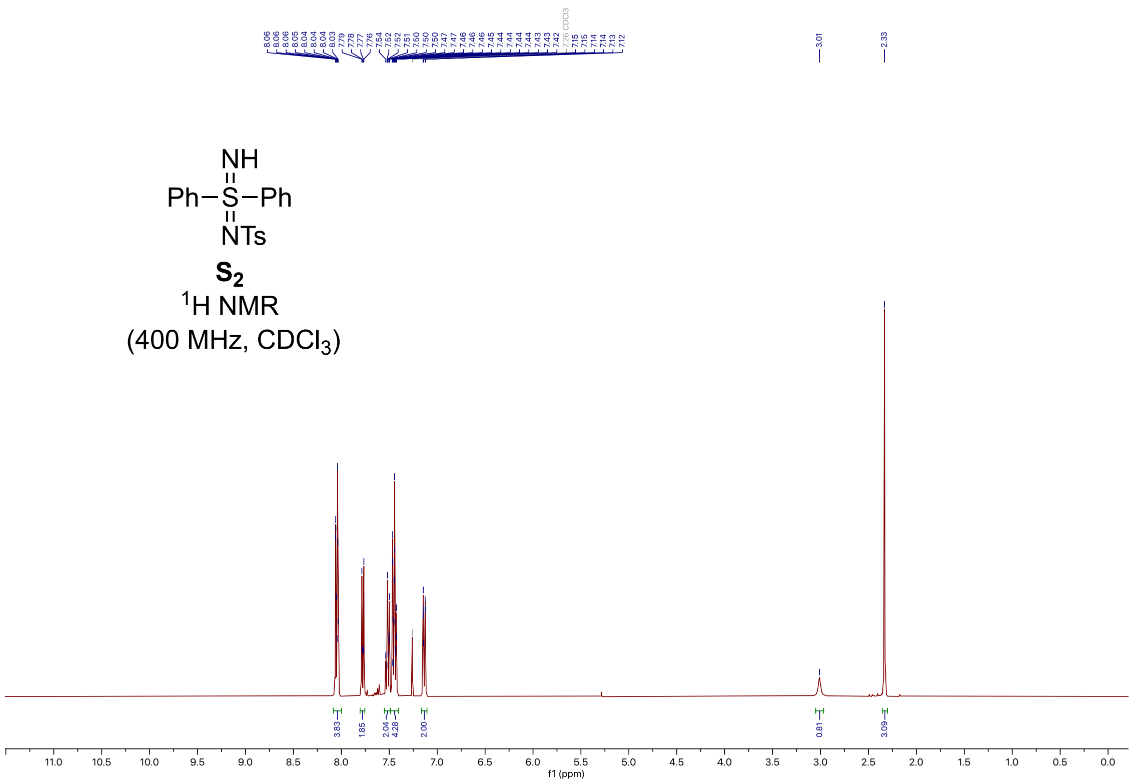
NH
Ph−S−Ph
NTs
S2
^{1}H NMR
(400 MHz, $CDCl_3$)
f1 (ppm)

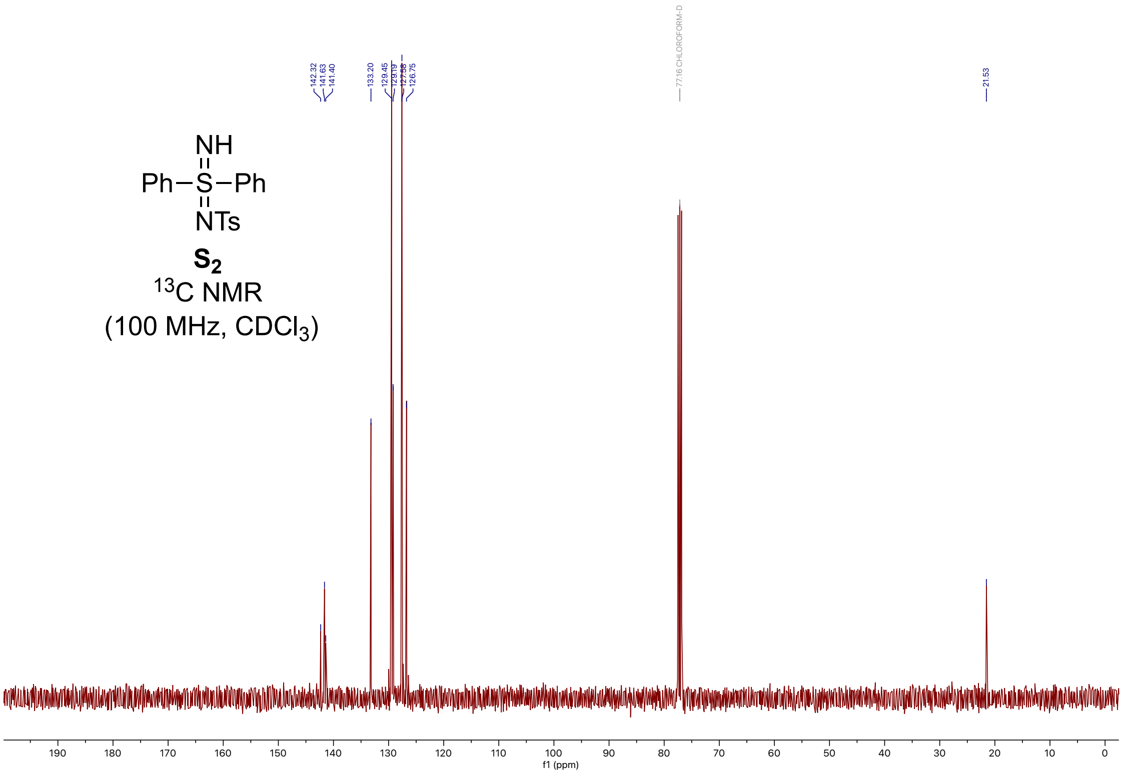
NH
Ph−S−Ph
NTs
S2
^{13}C NMR
(100 MHz, $CDCl_3$)
f1 (ppm)

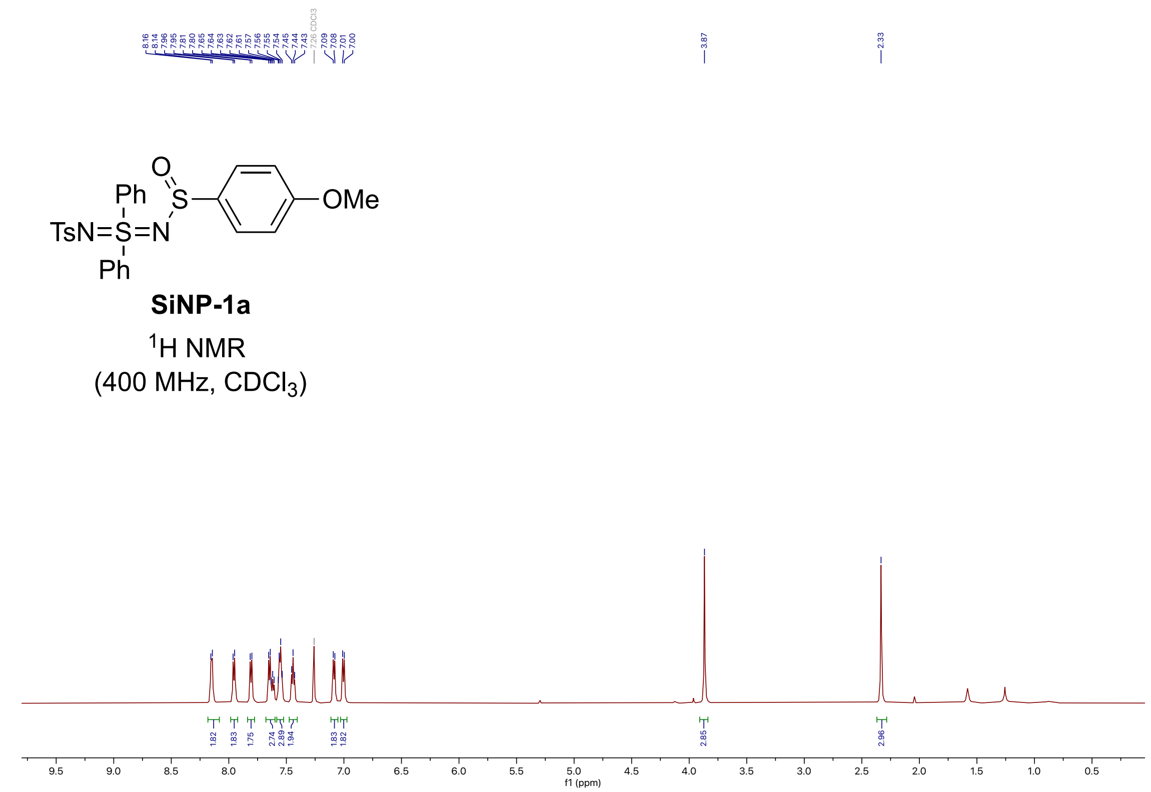
TsN=S=N
Ph
Ph
O
S
OMe
SiNP-1a
1H NMR
(400 MHz, CDCl3)
f1 (ppm)

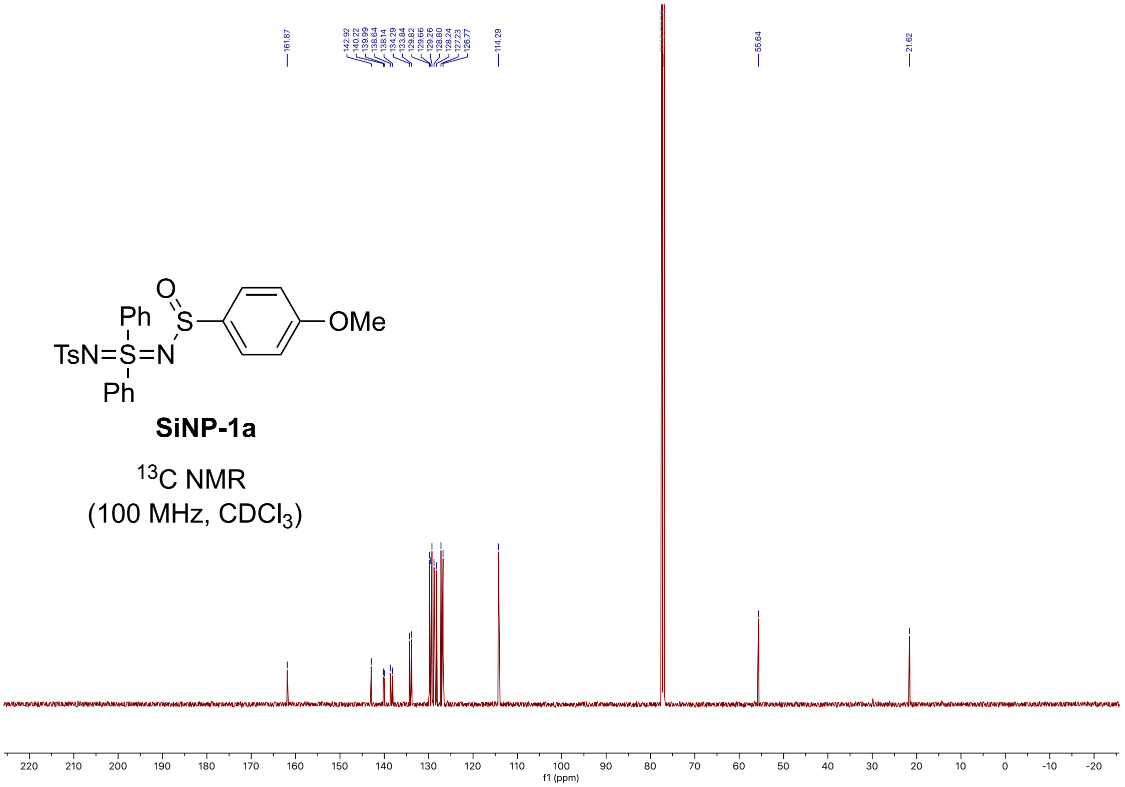
TsN=S=N
Ph
Ph
O
S
OMe
SiNP-1a
13C NMR
(100 MHz, CDCl3)
f1 (ppm)

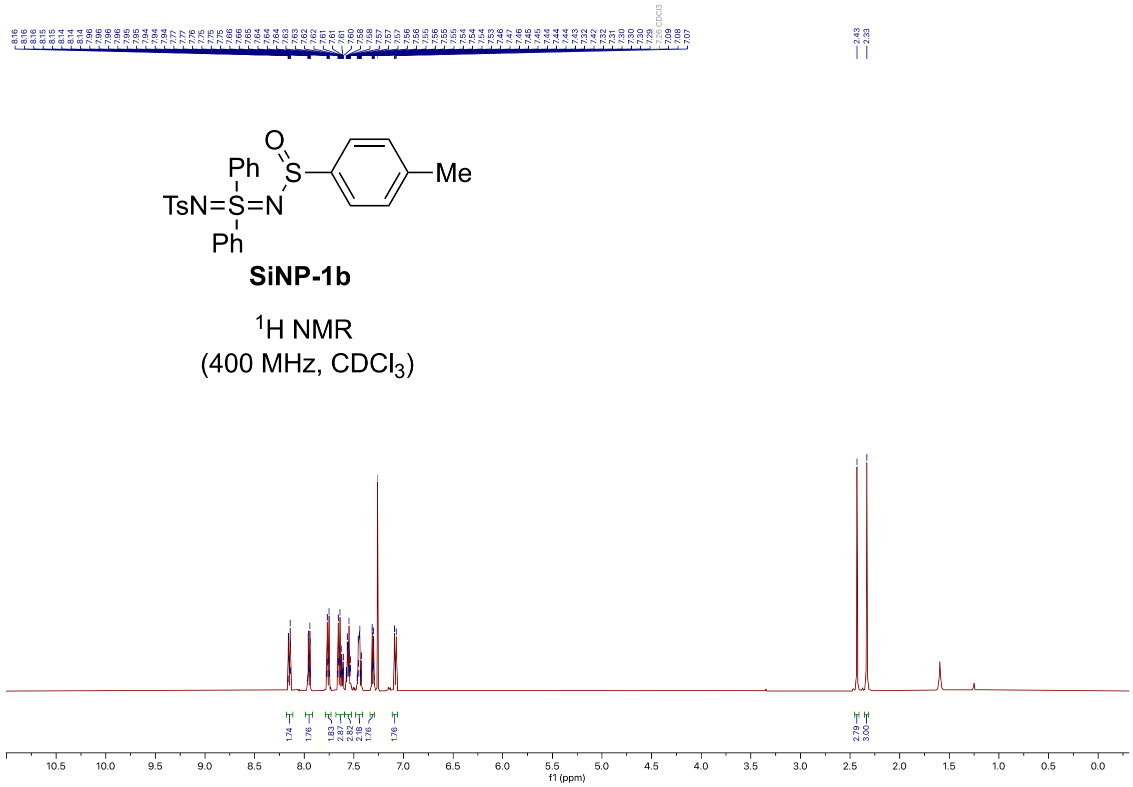

2.43
2.33
Ph
O
S
Me
TsN=S=N
Ph
SiNP-1b
^{1}H NMR
(400 MHz, $CDCl_3$)
1.74
1.76
1.83
2.87
2.82
2.18
1.76
1.76
2.79
3.00
f1 (ppm)

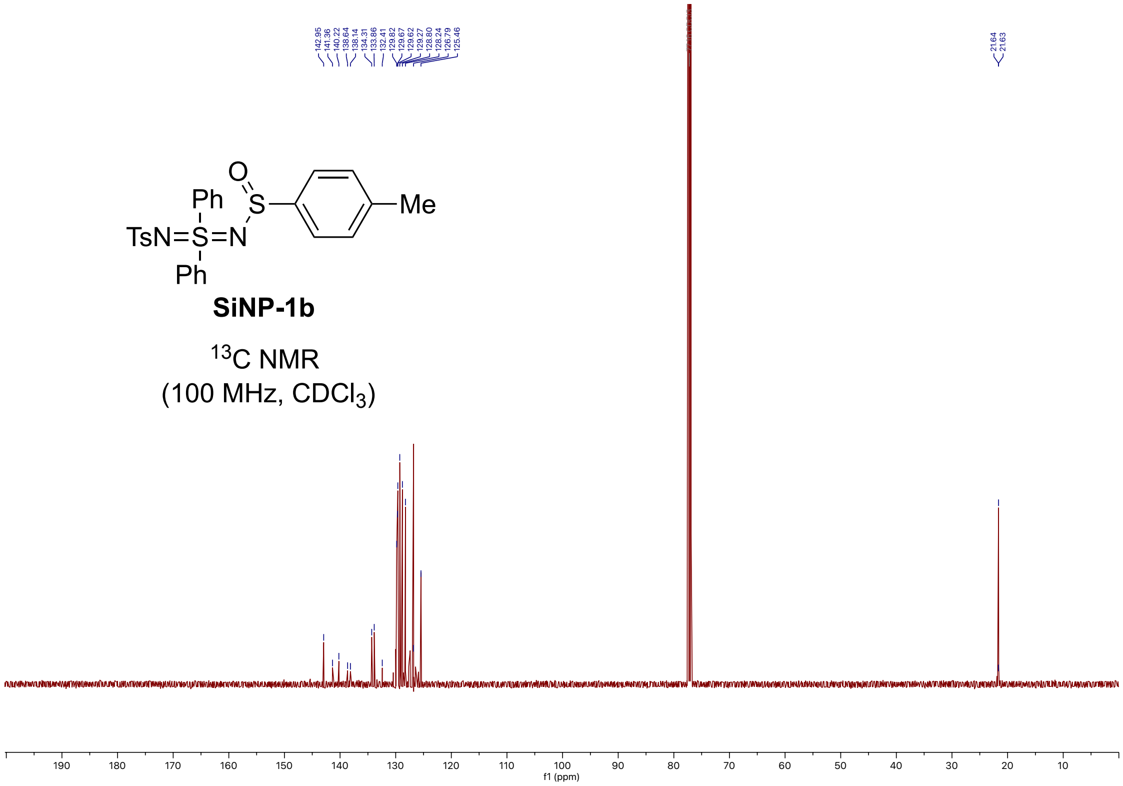

142.95
141.36
140.22
138.64
138.14
134.31
133.86
132.41
129.82
129.67
129.62
129.27
128.80
128.24
126.79
125.46
21.64
21.63
Ph
O
S
Me
TsN=S=N
Ph
SiNP-1b
^{13}C NMR
(100 MHz, $CDCl_3$)
f1 (ppm)

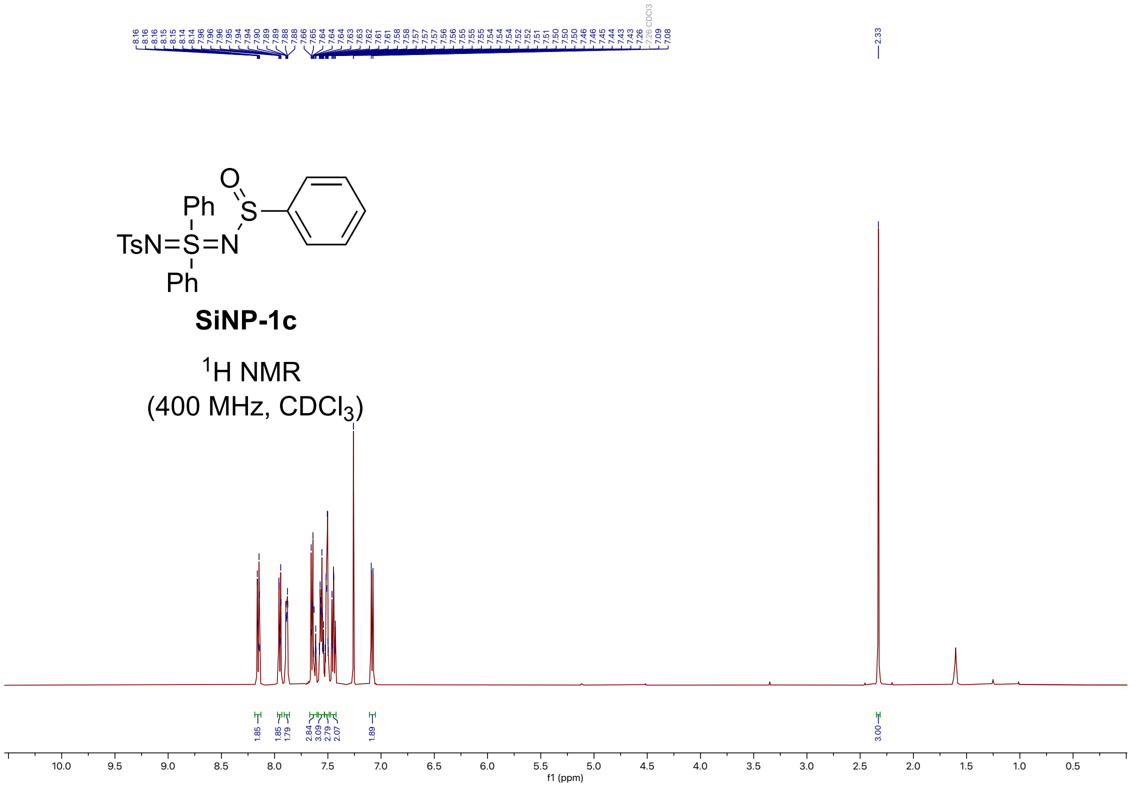
Ph
TsN=S=N
Ph
O
S
SiNP-1c
^{1}H NMR
(400 MHz, CDCl$_3$)
f1 (ppm)

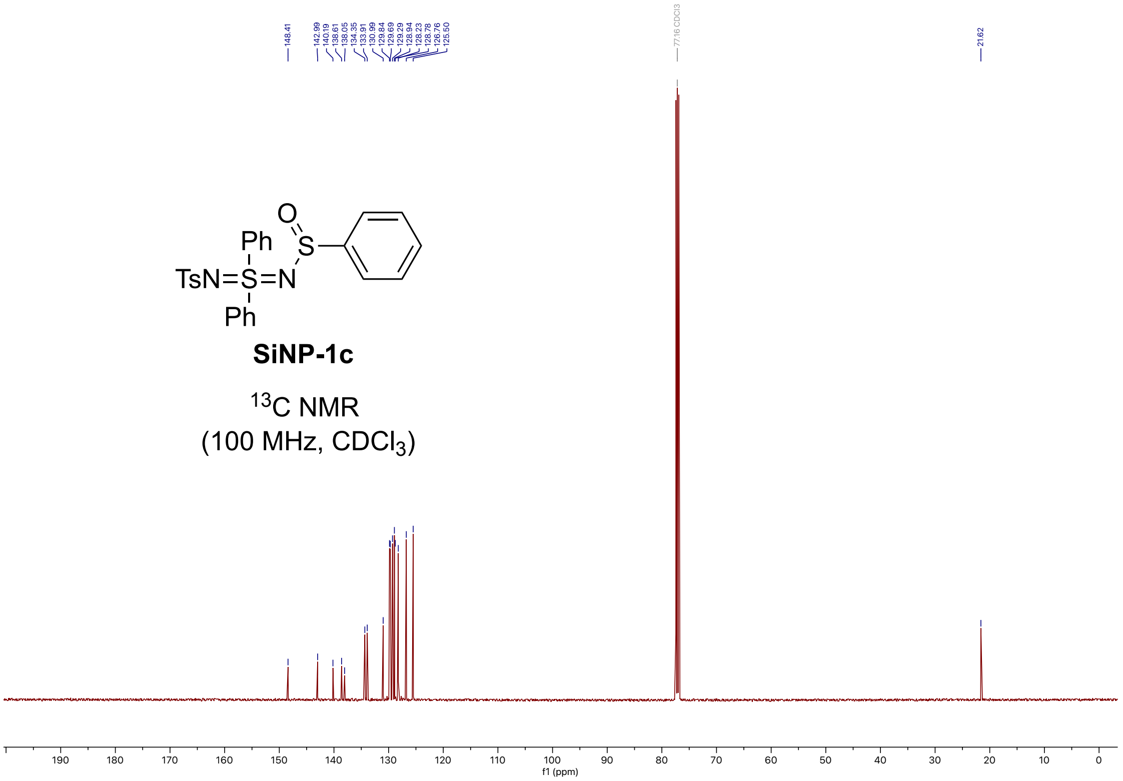
Ph
TsN=S=N
Ph
O
S
SiNP-1c
^{13}C NMR
(100 MHz, CDCl$_3$)
f1 (ppm)

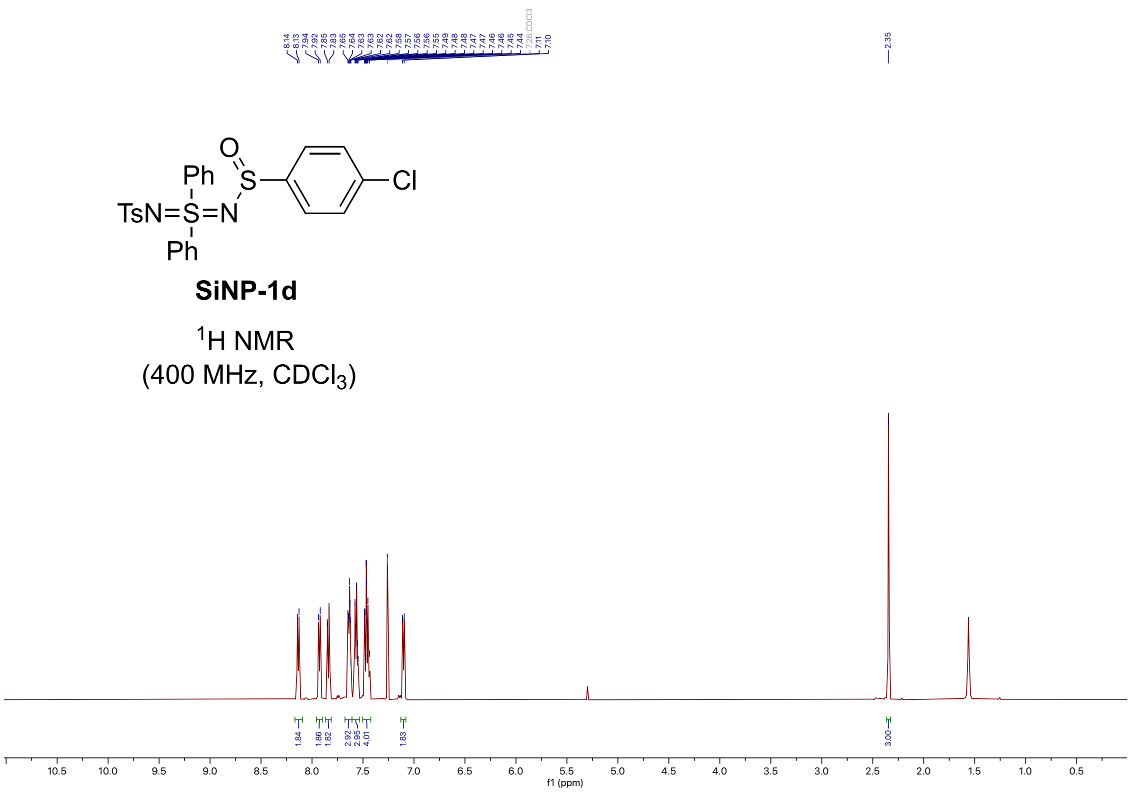
Ph
TsN=S=N
Ph
O
S
Cl
SiNP-1d
^{1}H NMR
(400 MHz, $CDCl_3$)
f1 (ppm)

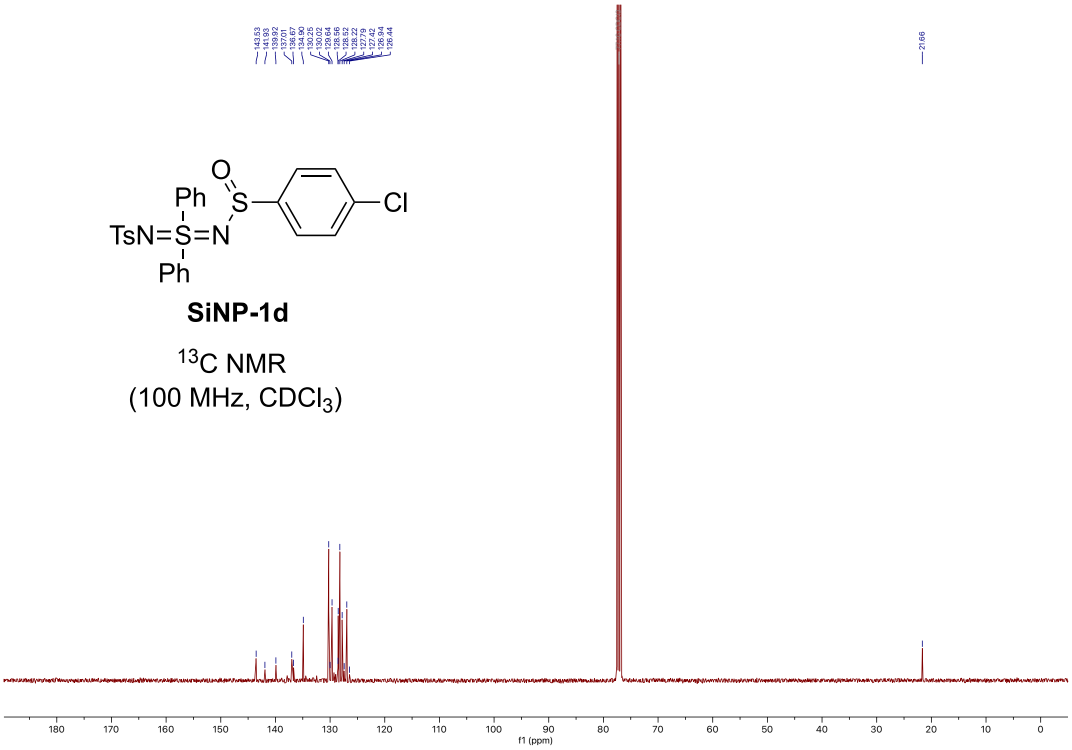
Ph
TsN=S=N
Ph
O
S
Cl
SiNP-1d
^{13}C NMR
(100 MHz, $CDCl_3$)
f1 (ppm)

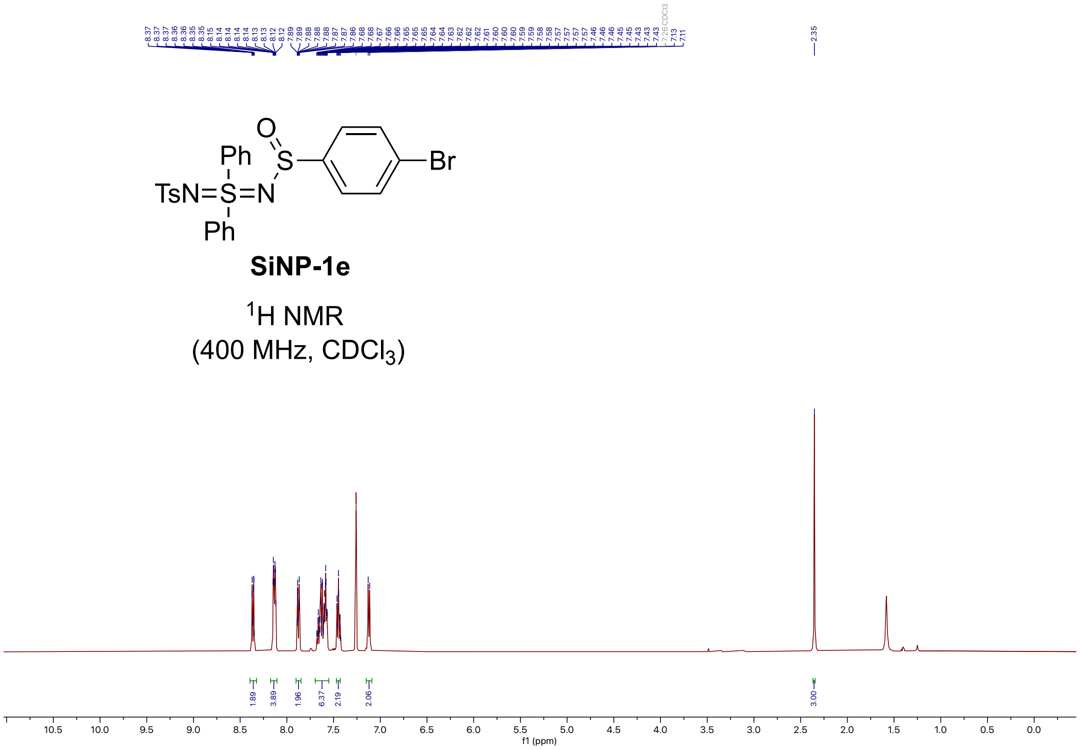
Ph
TsN=S=N
Ph
O
S
Br
SiNP-1e
1H NMR
(400 MHz, CDCl3)
f1 (ppm)

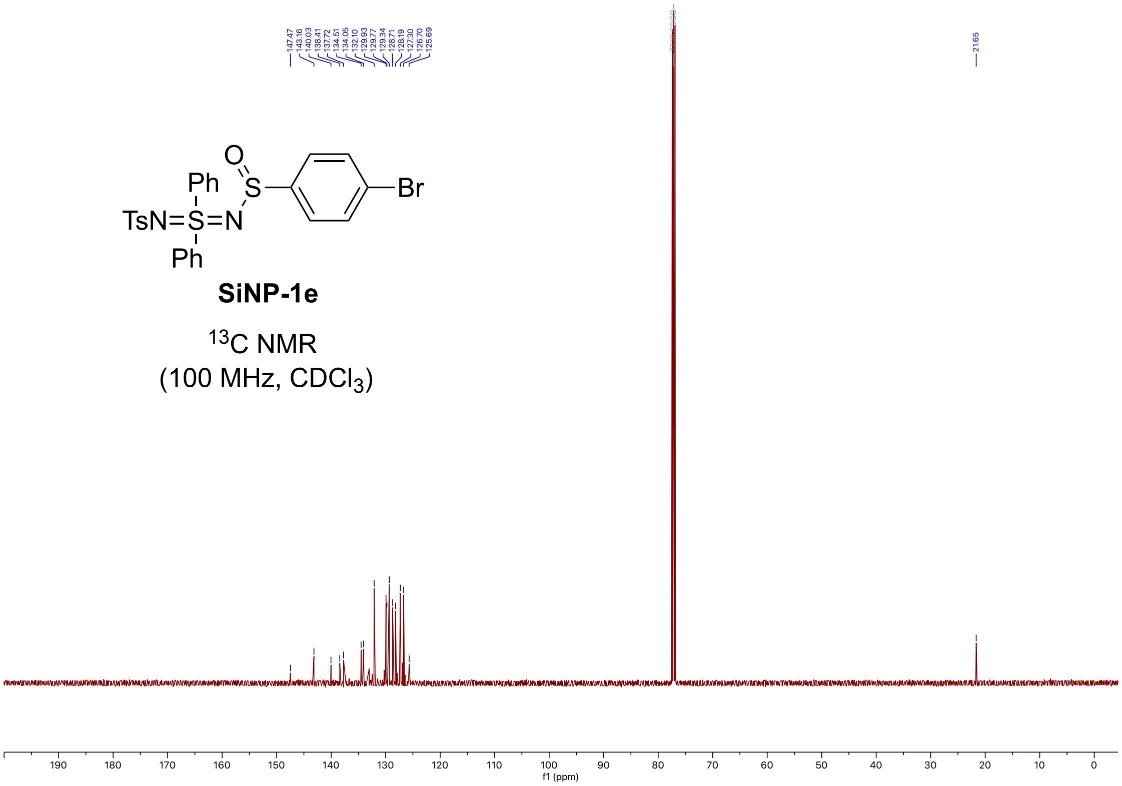
Ph
TsN=S=N
Ph
O
S
Br
SiNP-1e
13C NMR
(100 MHz, CDCl3)
f1 (ppm)

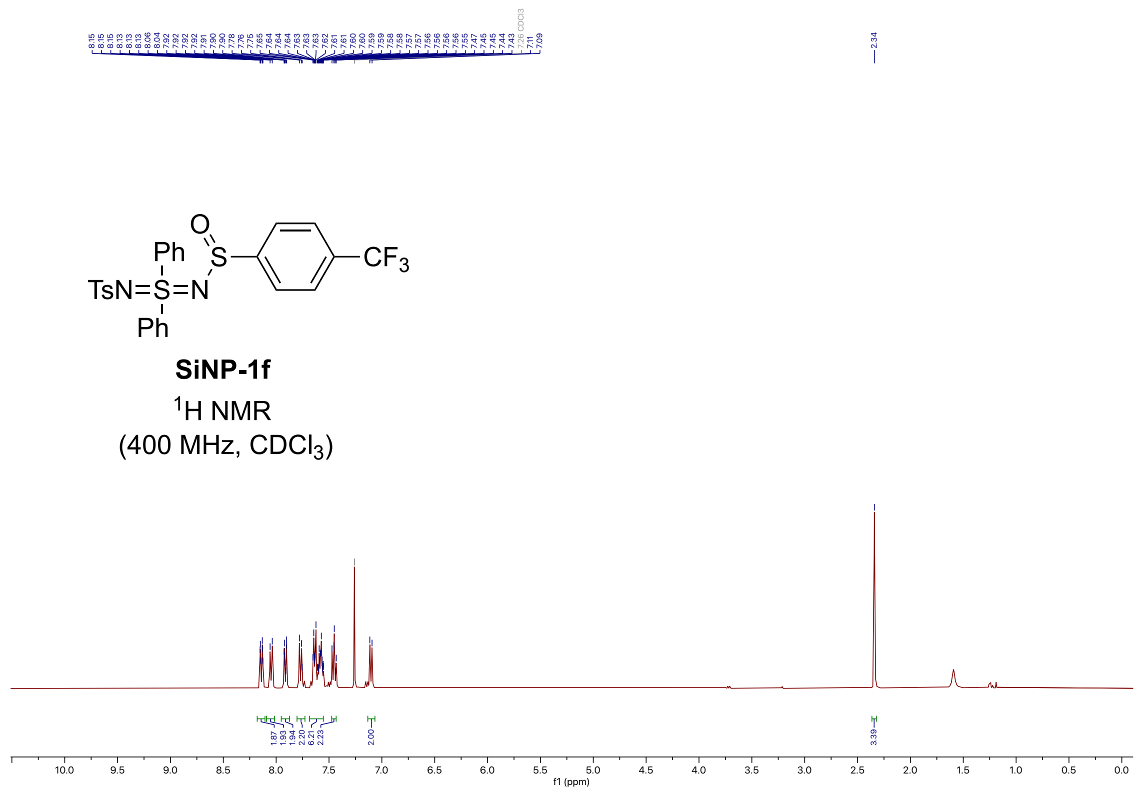
SiNP-1f
1H NMR
(400 MHz, CDCl3)
2.34
f1 (ppm)

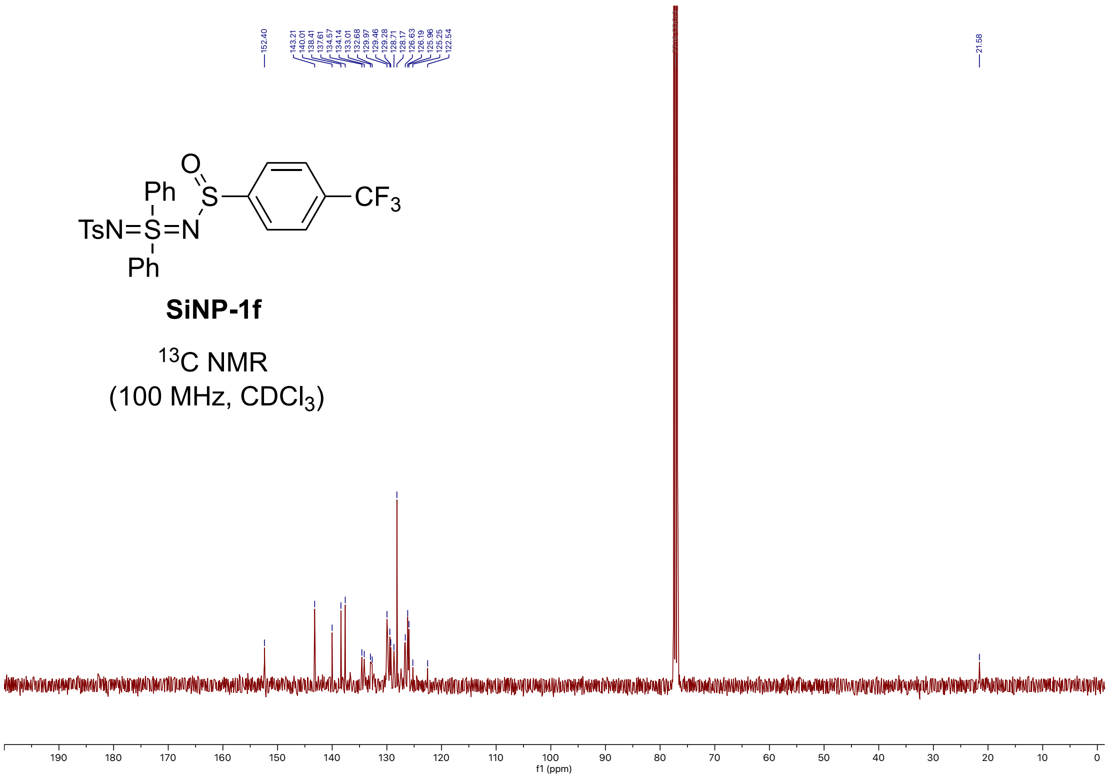
SiNP-1f
13C NMR
(100 MHz, CDCl3)
152.40
21.58
f1 (ppm)

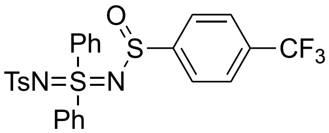

SiNP-1f (^{19}F NMR,376 MHz, $CDCl_3$)

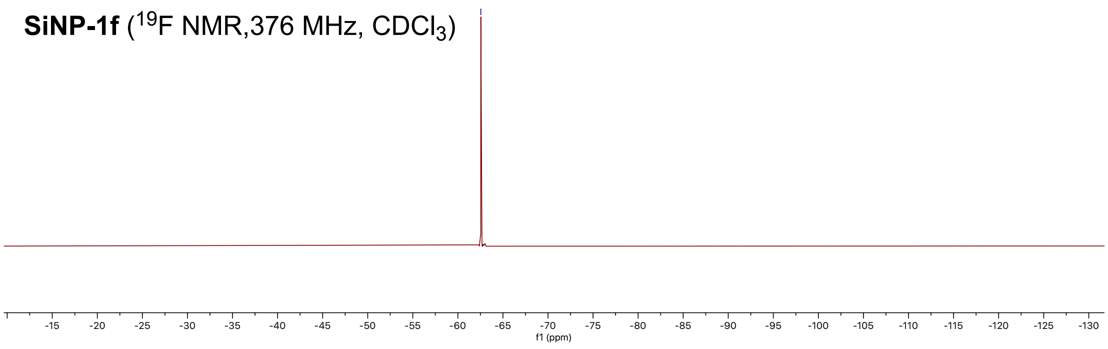

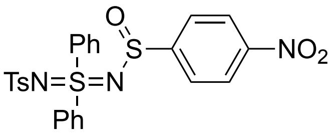

SiNP-1g

^1H NMR
(400 MHz, $CDCl_3$)

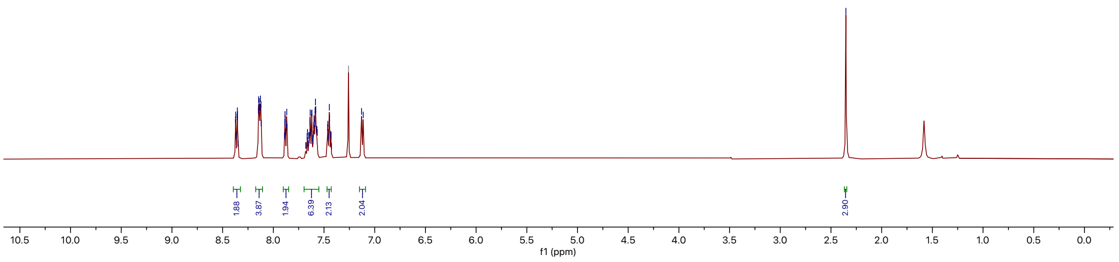

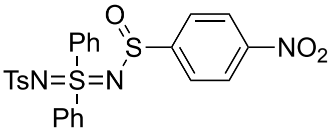

SiNP-1g

^{13}C NMR
(100 MHz, $CDCl_3$)

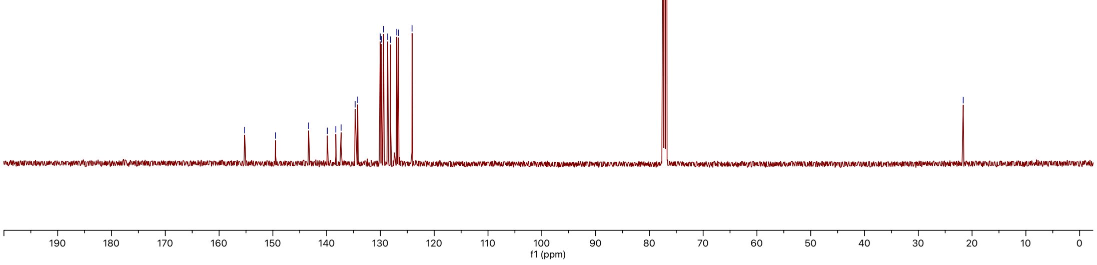

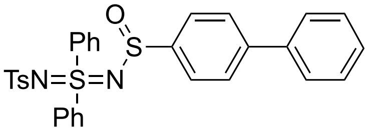

SiNP-1h

^{1}H NMR

(400 MHz, $CDCl_3$)

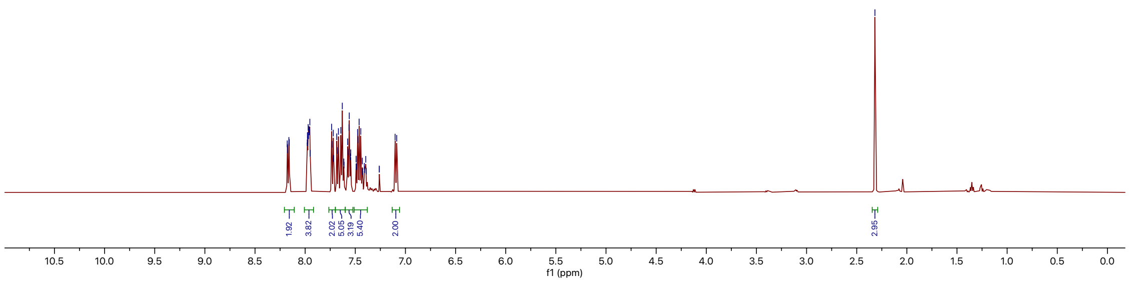

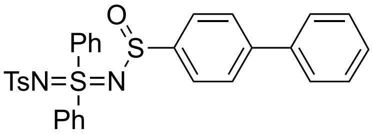

SiNP-1h

^{13}C NMR

(100 MHz, $CDCl_3$)

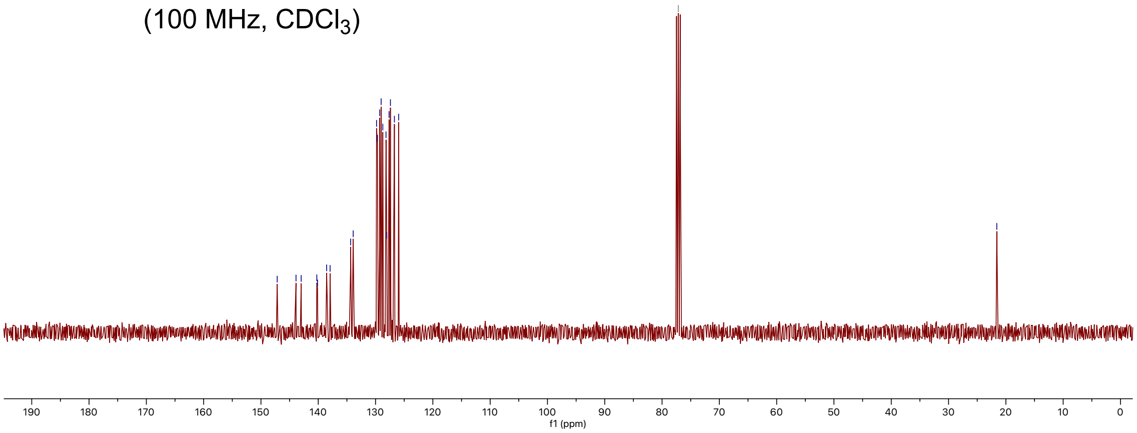

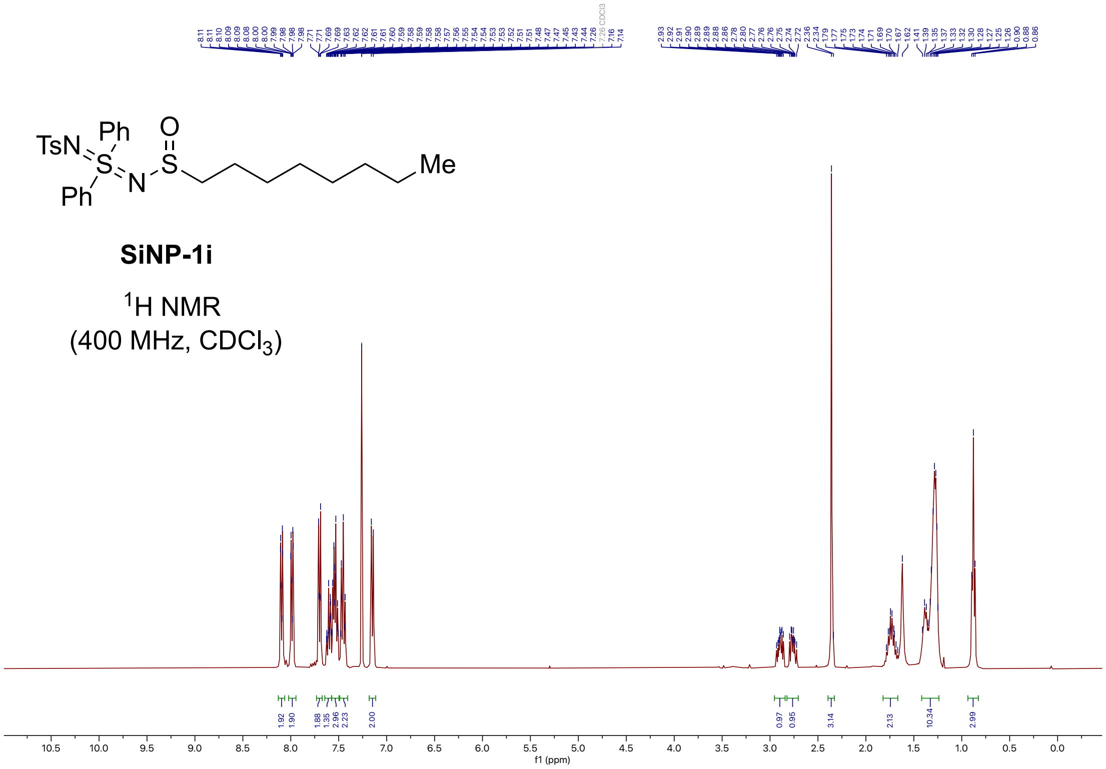
SiNP-1i
^{1}H NMR
(400 MHz, $CDCl_3$)

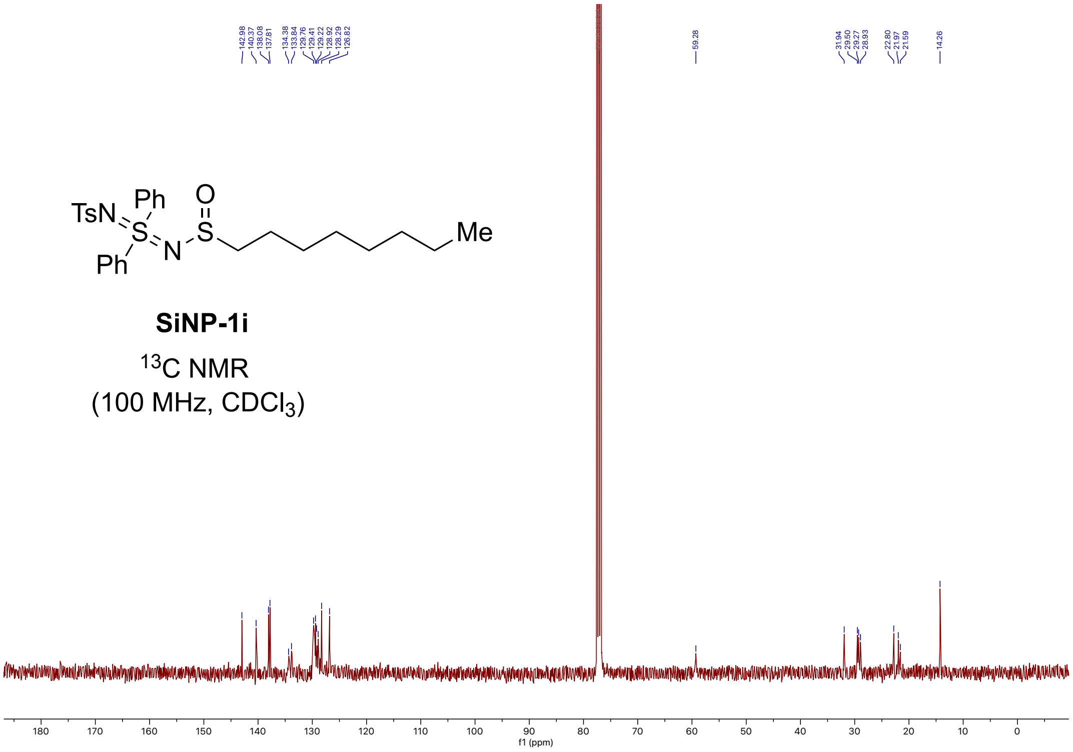
SiNP-1i
^{13}C NMR
(100 MHz, $CDCl_3$)

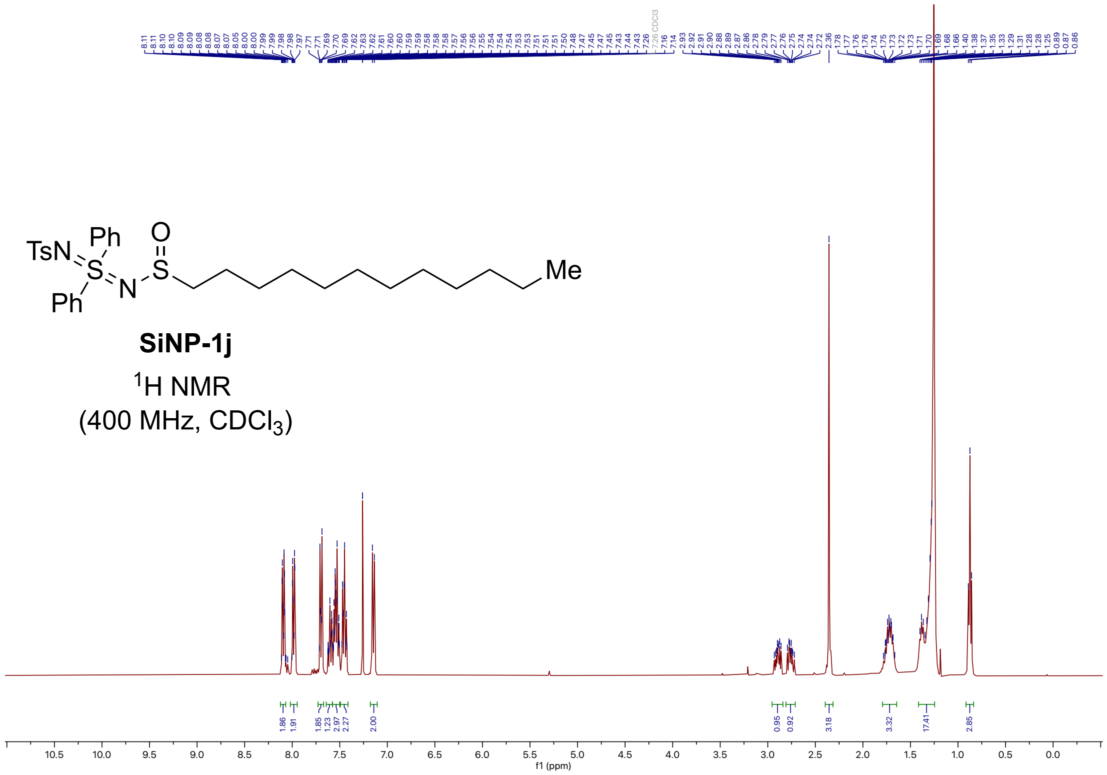
TsN
Ph
O
S
N
S
Me
Ph
SiNP-1j
^{1}H NMR
(400 MHz, CDCl$_3$)
f1 (ppm)

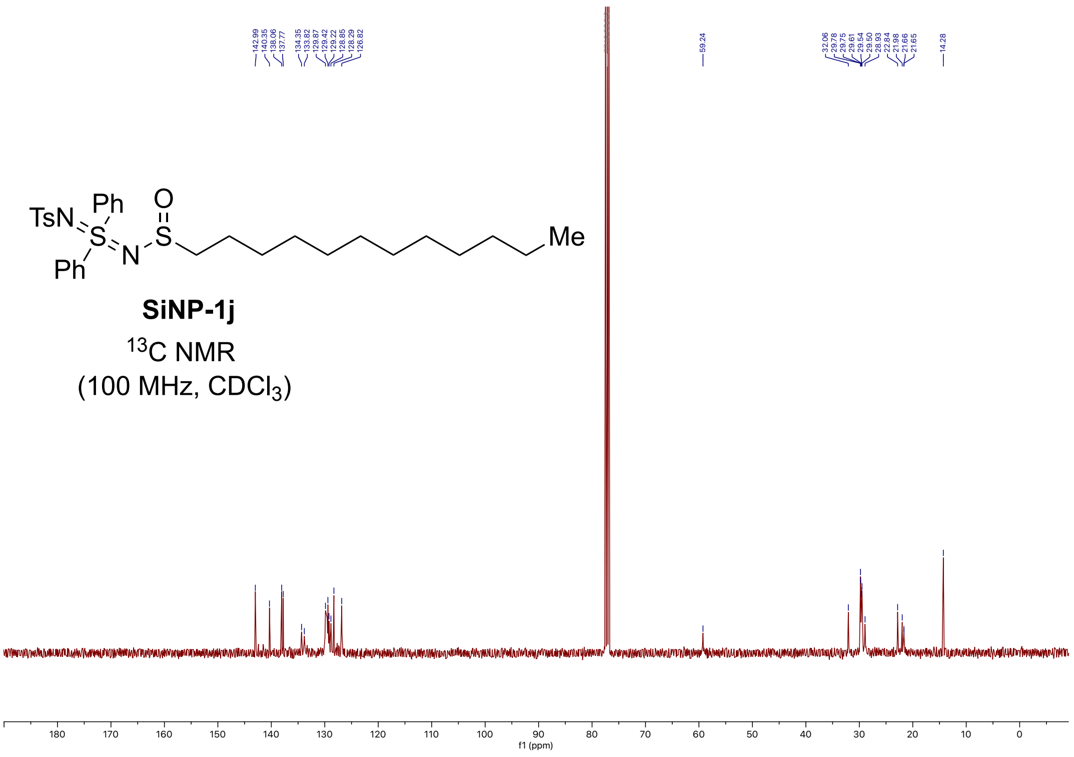
TsN
Ph
O
S
N
S
Me
Ph
SiNP-1j
^{13}C NMR
(100 MHz, CDCl$_3$)
f1 (ppm)

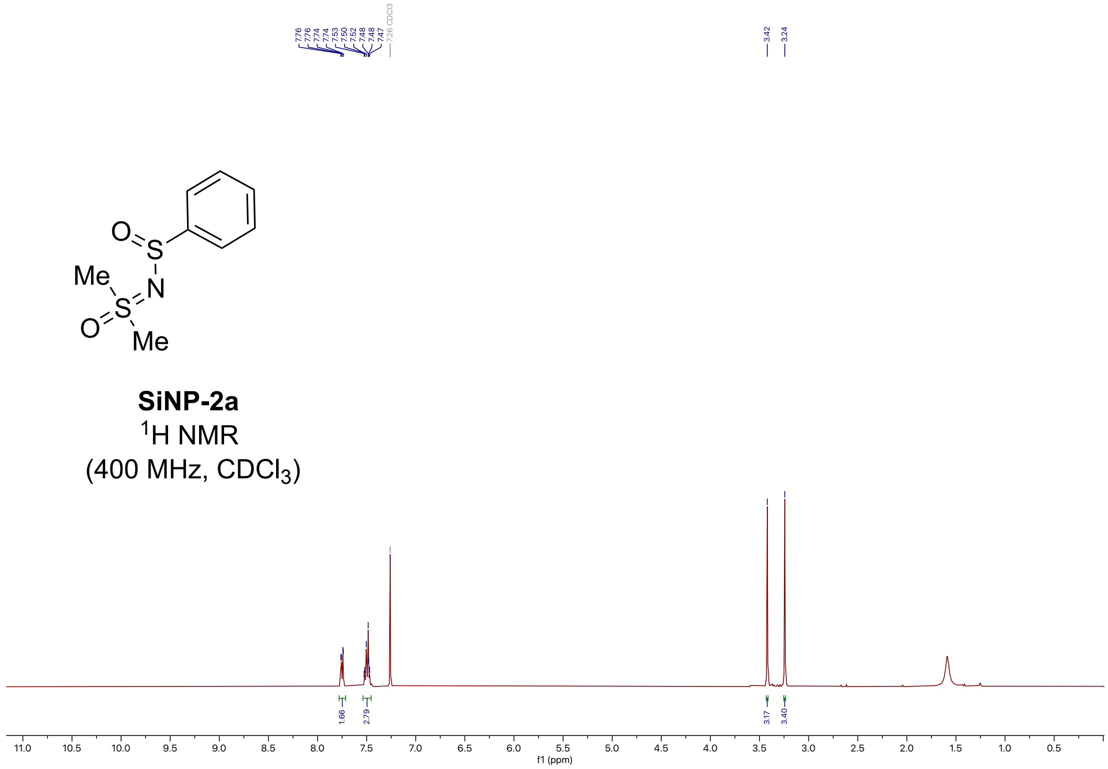

SiNP-2a
^{1}H NMR
(400 MHz, $CDCl_3$)

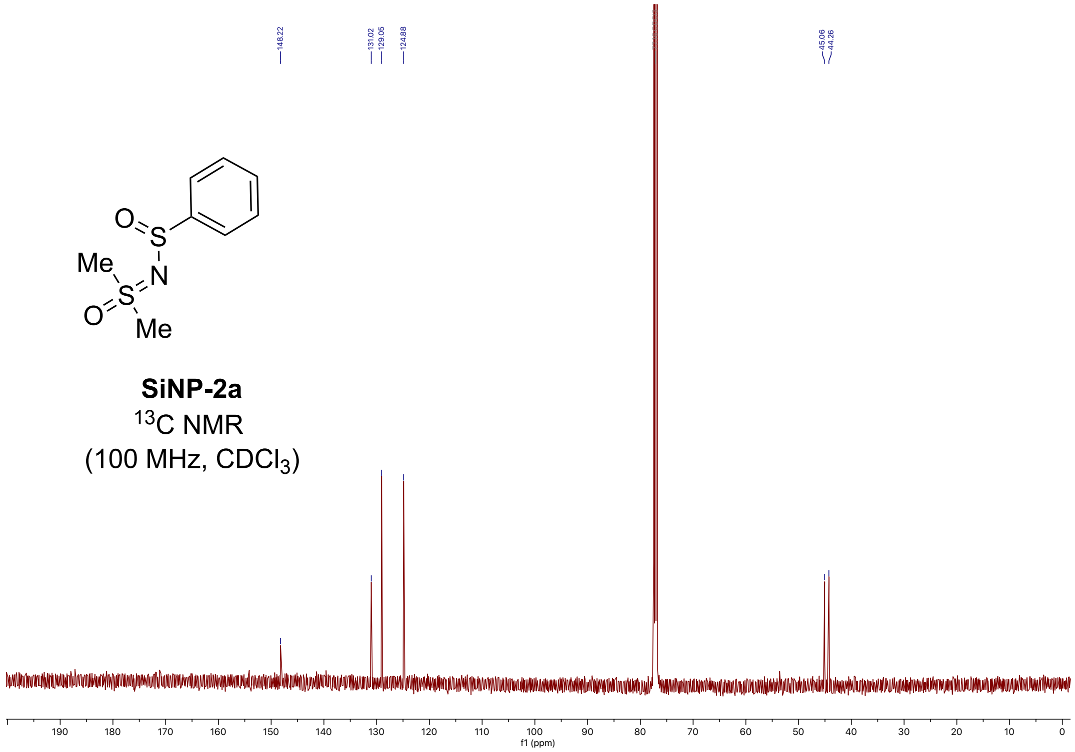

SiNP-2a
^{13}C NMR
(100 MHz, $CDCl_3$)

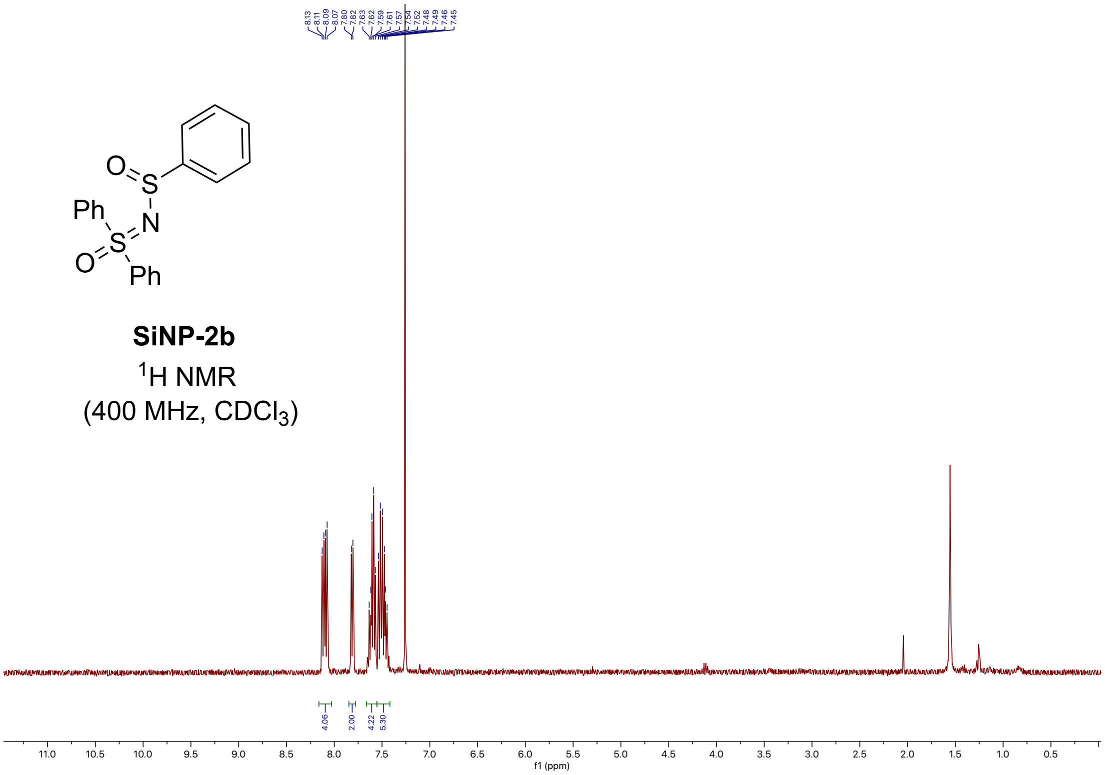

SiNP-2b
^{1}H NMR
(400 MHz, $CDCl_3$)

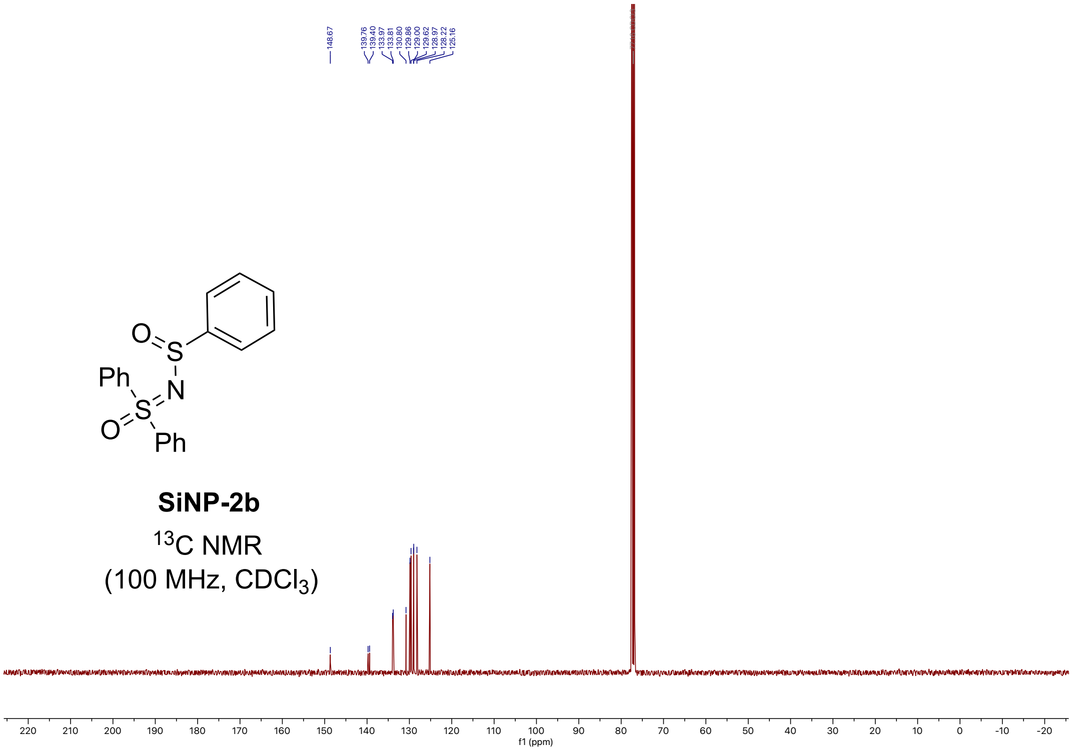

SiNP-2b
^{13}C NMR
(100 MHz, $CDCl_3$)

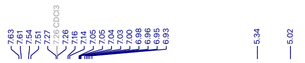

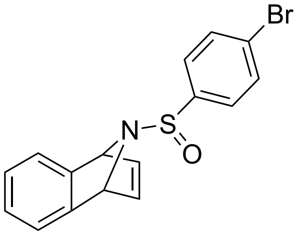

SiNP-3

^{1}H NMR
(400 MHz, $CDCl_3$)

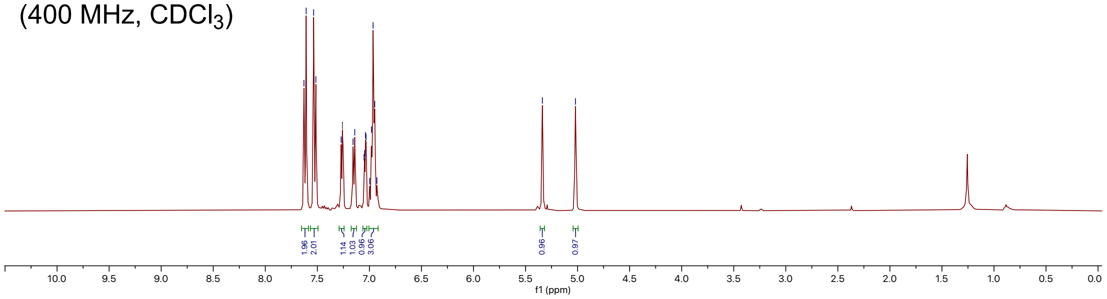

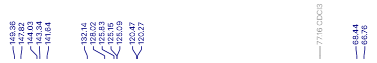

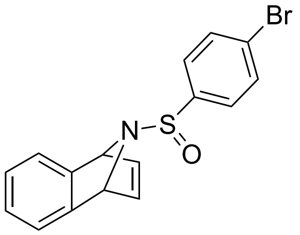

SiNP-3

^{13}C NMR
(100 MHz, $CDCl_3$)

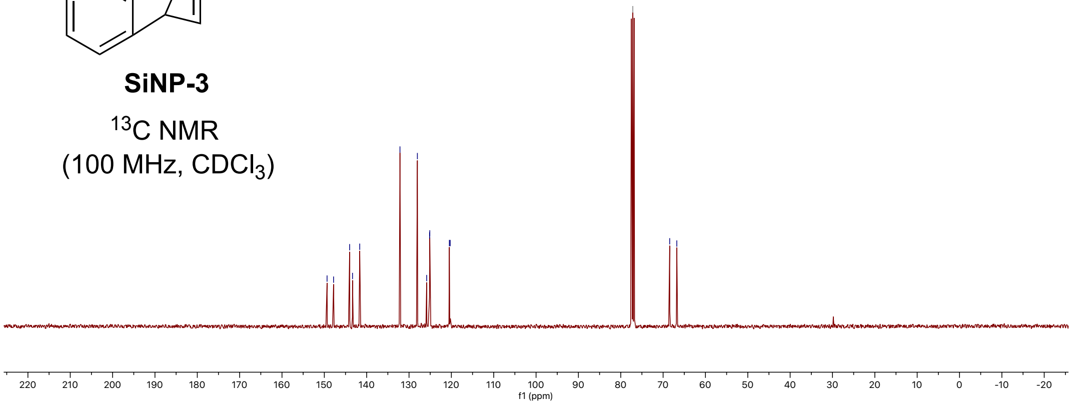

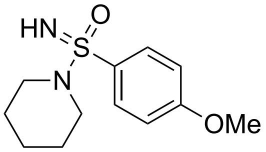

2aa
^{1}H NMR
(400 MHz, $CDCl_3$)

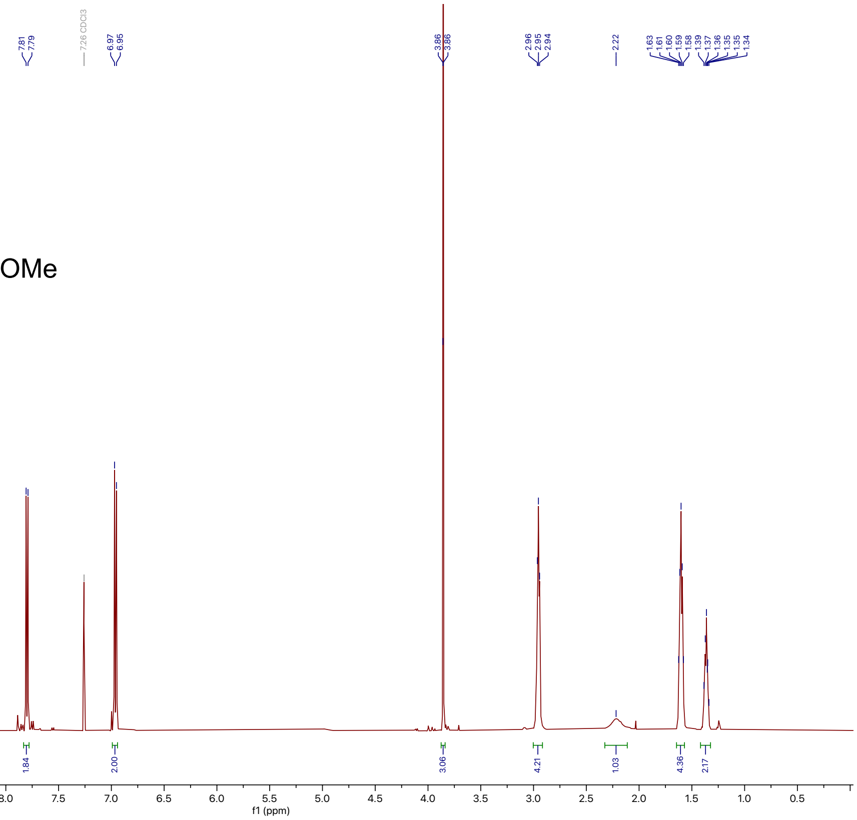

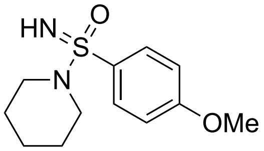

2aa
^{13}C NMR
(100 MHz, $CDCl_3$)

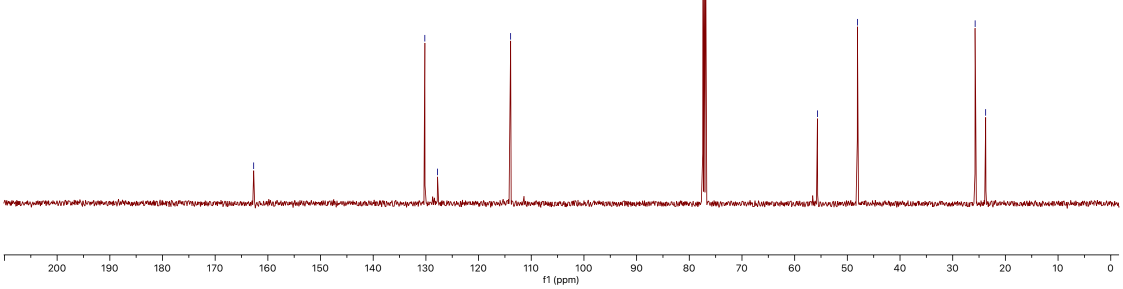

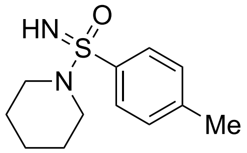

2ab
^{1}H NMR
(400 MHz, $CDCl_3$)

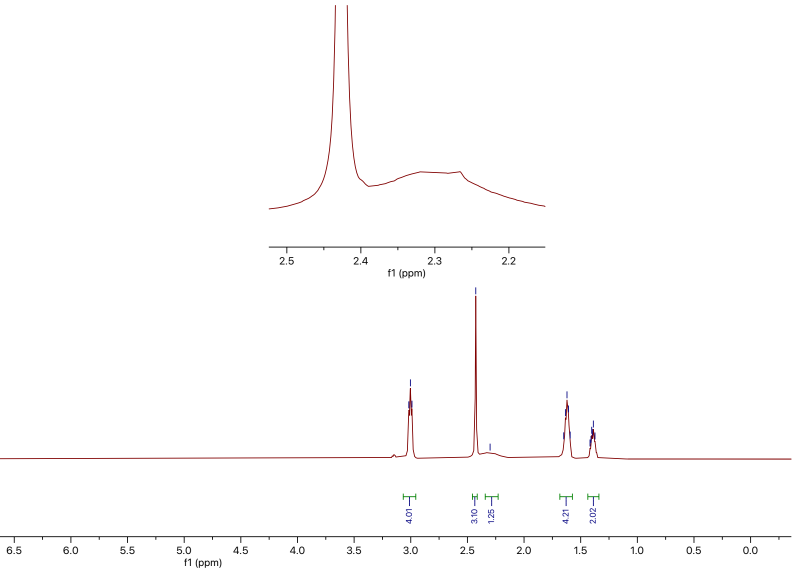

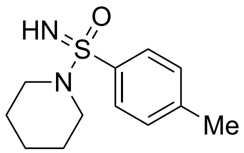

2ab
^{13}C NMR
(100 MHz, $CDCl_3$)

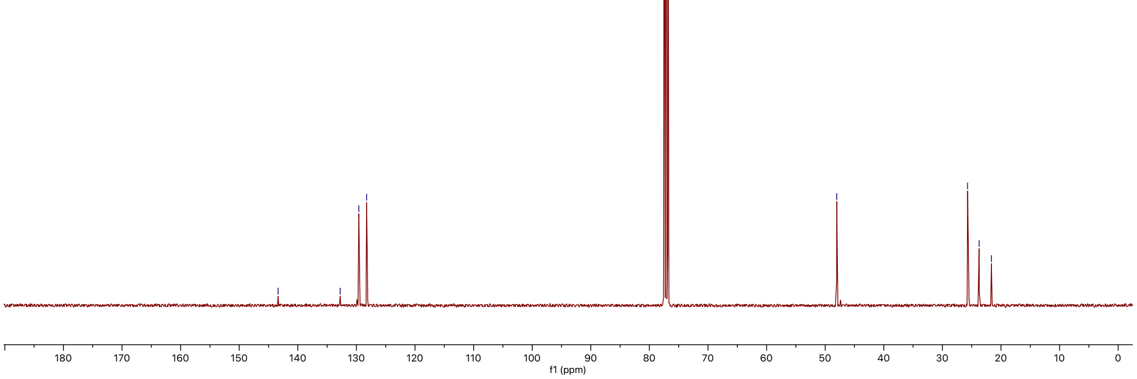

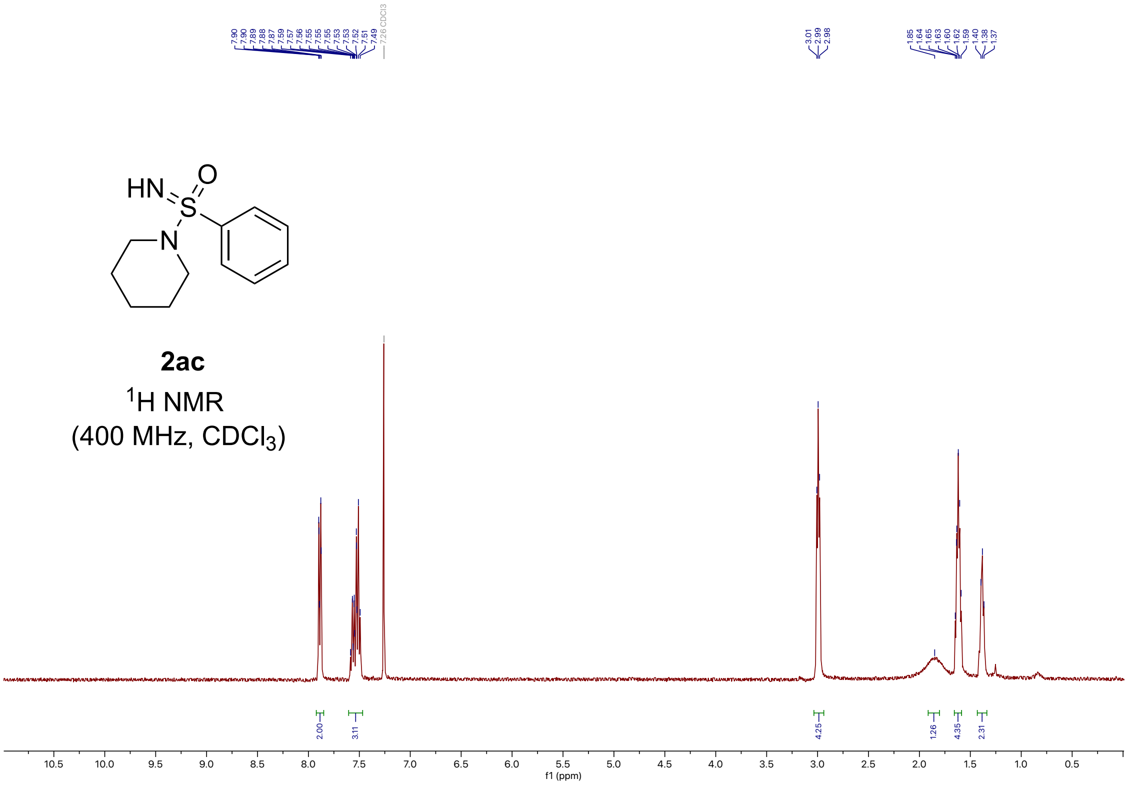
2ac
¹H NMR
(400 MHz, CDCl3)
f1 (ppm)

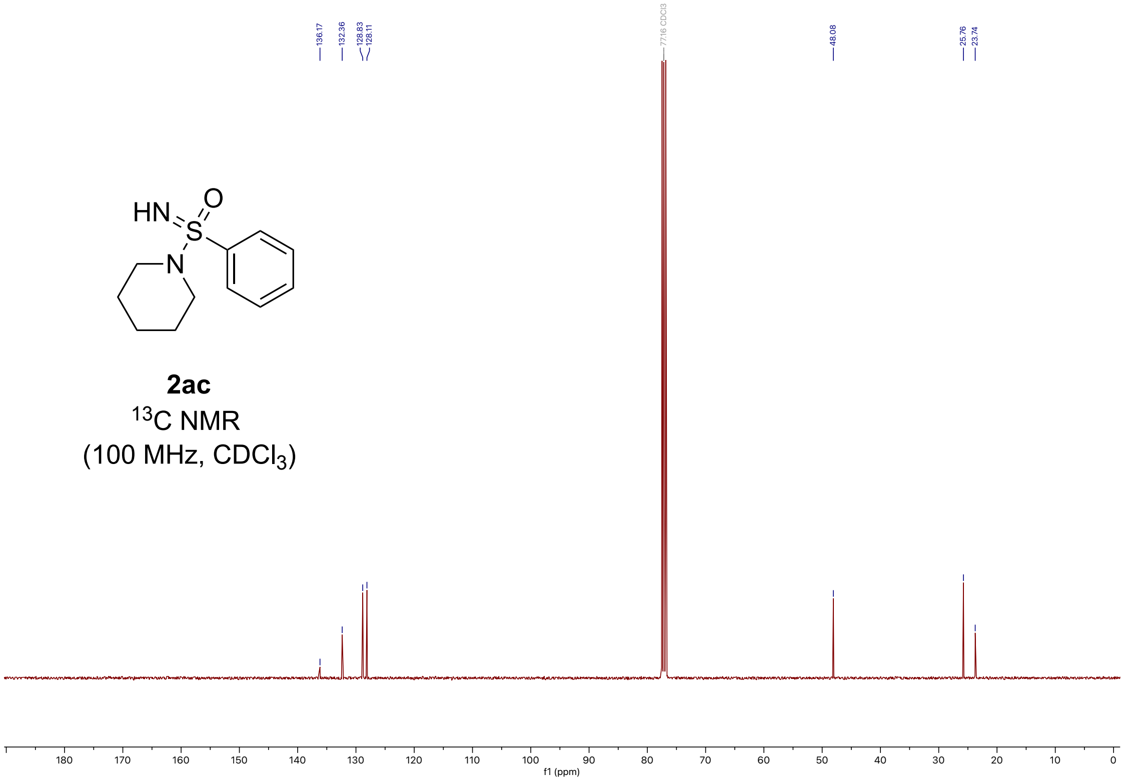
2ac
¹³C NMR
(100 MHz, CDCl3)
f1 (ppm)

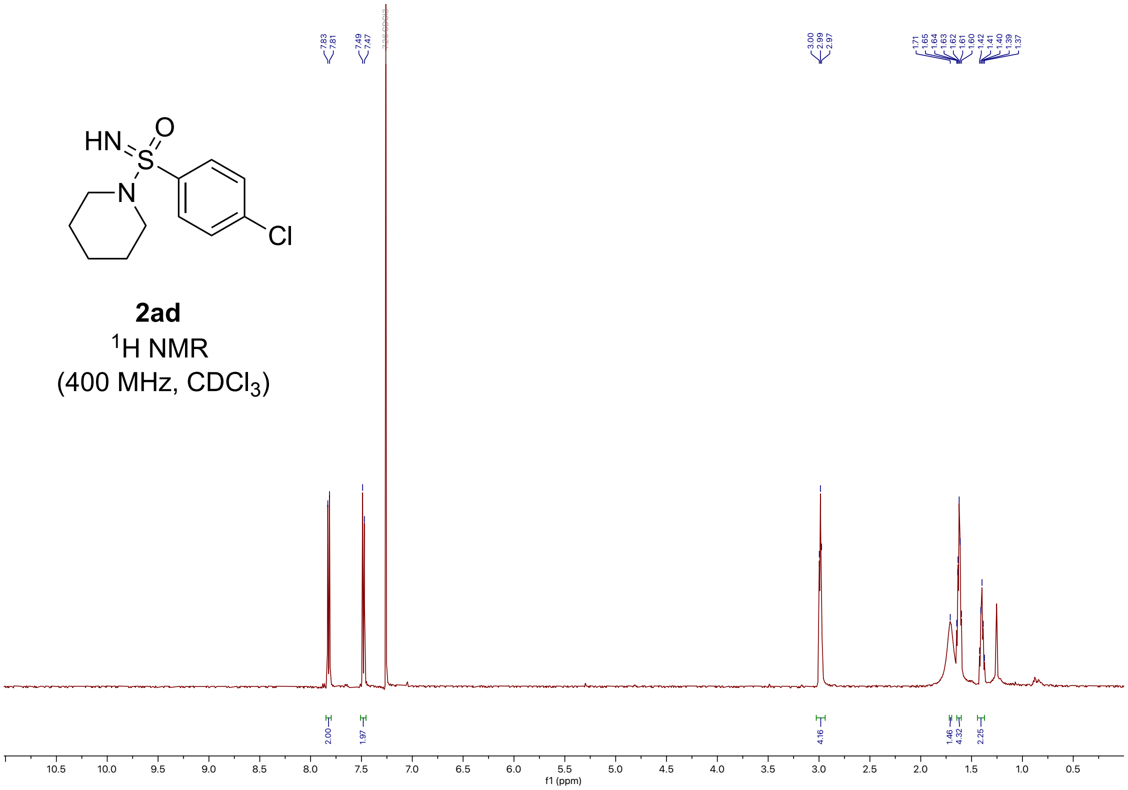
7.83
7.81
7.49
7.47
3.00
2.99
2.97
1.71
1.65
1.64
1.63
1.62
1.61
1.60
1.42
1.41
1.40
1.39
1.37
HN
O
S
N
Cl
2ad
1H NMR
(400 MHz, CDCl3)
2.00
1.97
4.16
1.46
4.32
2.25
10.5
10.0
9.5
9.0
8.5
8.0
7.5
7.0
6.5
6.0
5.5
5.0
4.5
4.0
3.5
3.0
2.5
2.0
1.5
1.0
0.5
f1 (ppm)

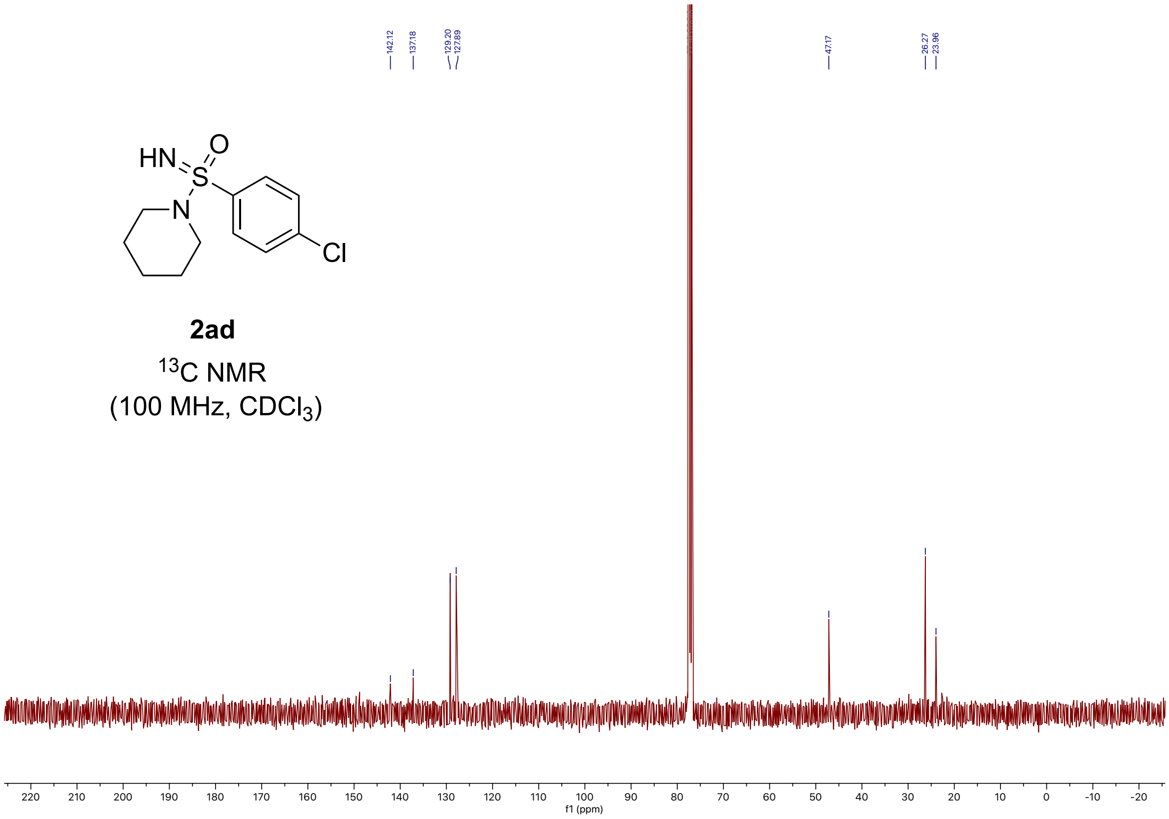
142.12
137.18
129.20
127.89
47.17
26.27
23.96
HN
O
S
N
Cl
2ad
13C NMR
(100 MHz, CDCl3)
220
210
200
190
180
170
160
150
140
130
120
110
100
90
80
70
60
50
40
30
20
10
0
-10
-20
f1 (ppm)

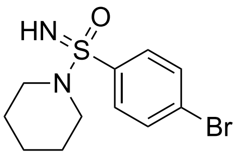

2ae

^{1}H NMR

(400 MHz, $CDCl_3$)

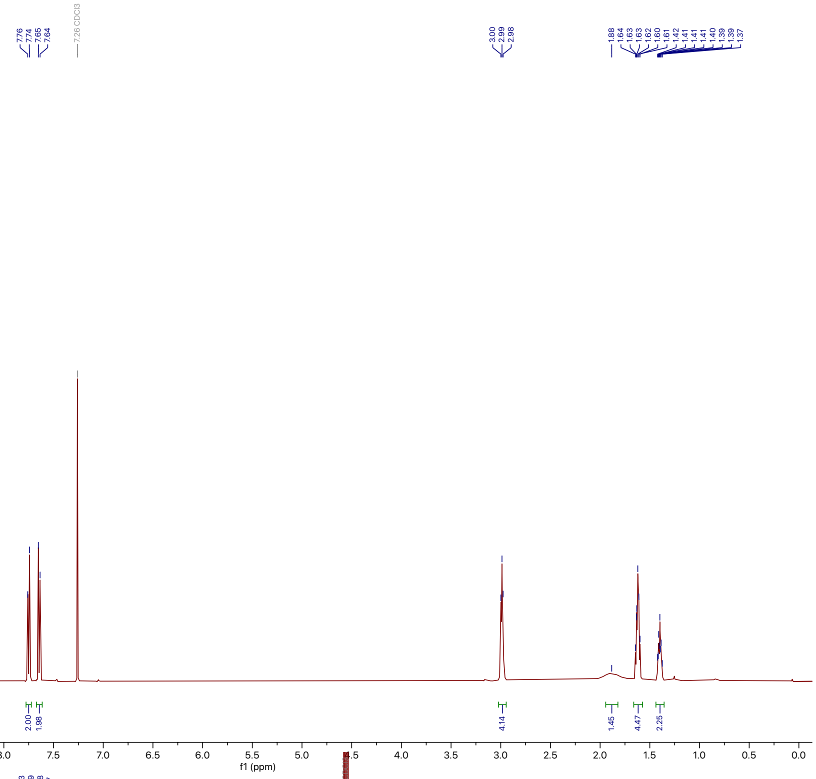

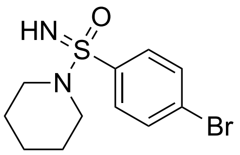

2ae

^{13}C NMR

(100 MHz, $CDCl_3$)

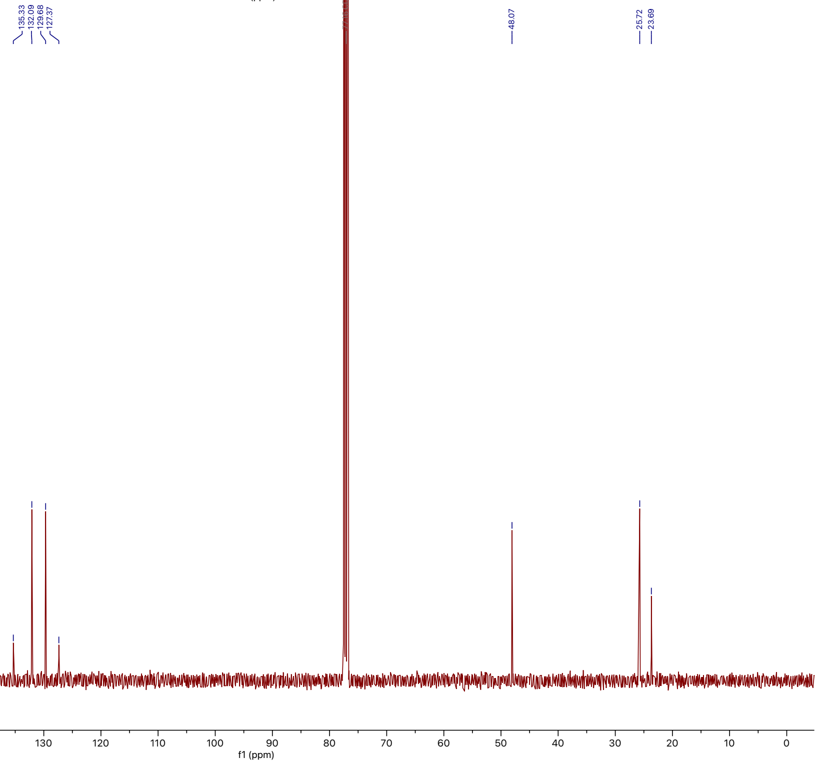

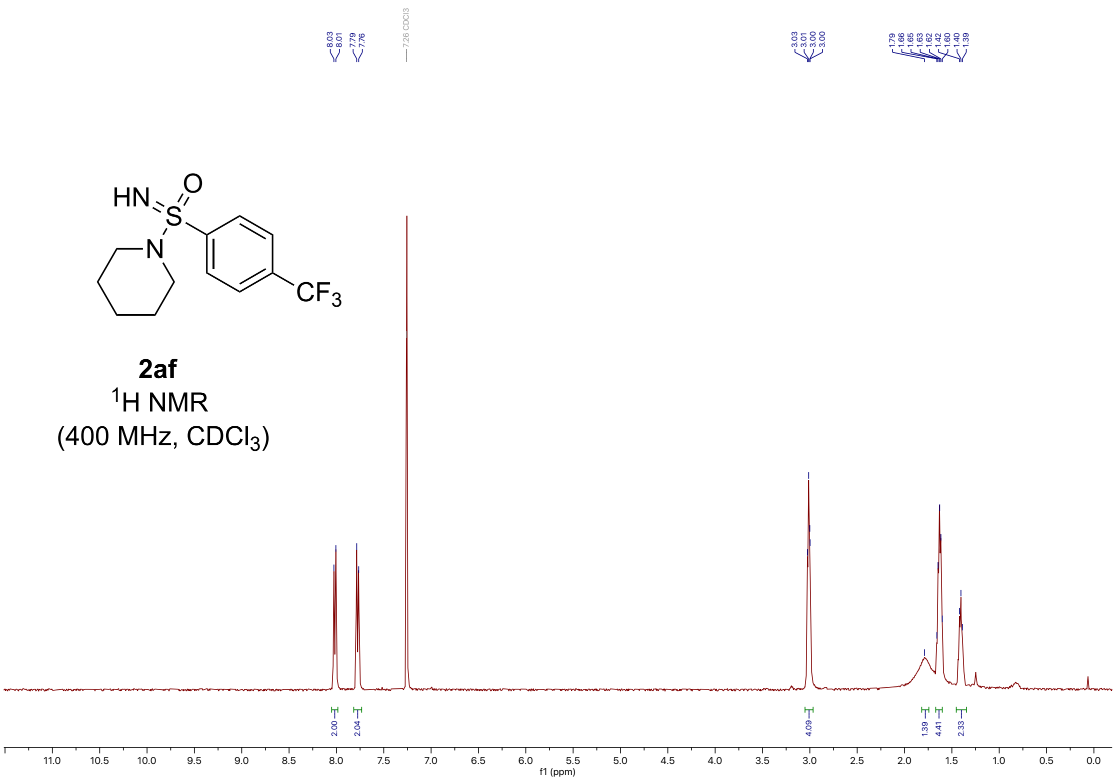
8.03
8.01
7.79
7.76
7.26 CDCl3
3.03
3.01
3.00
3.00
HN
S
O
N
CF3
2af
1H NMR
(400 MHz, CDCl3)
2.00
2.04
4.09
1.39
4.41
2.33
f1 (ppm)

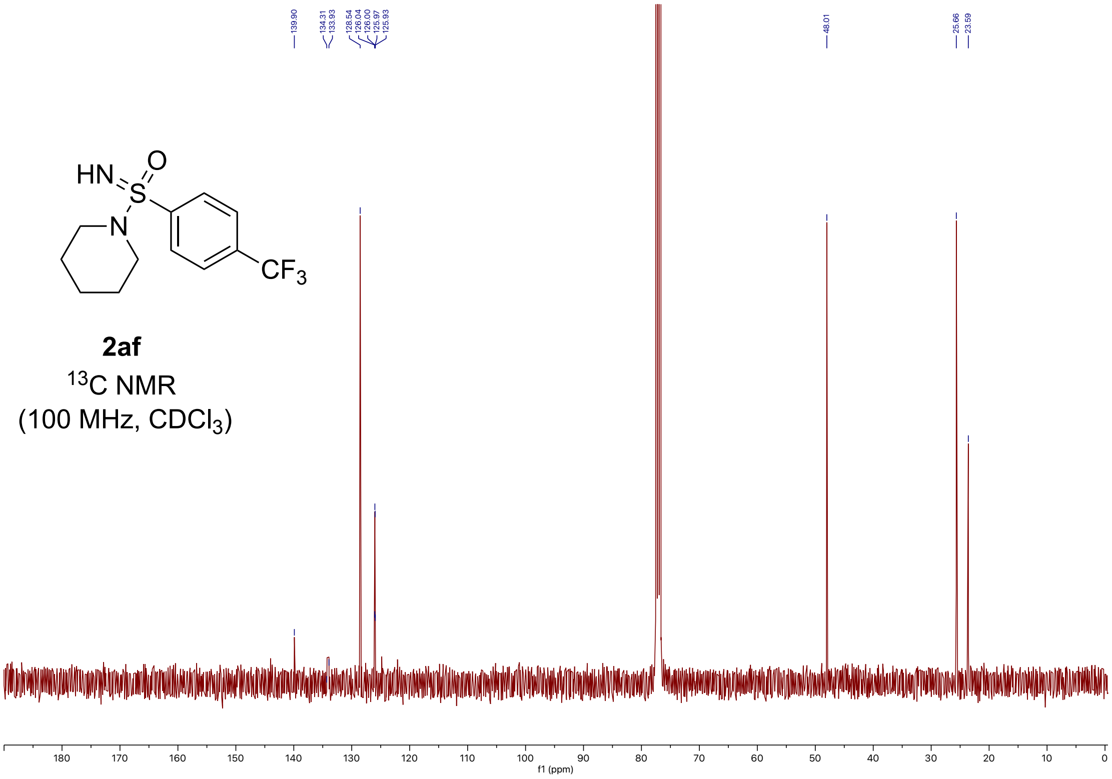
139.90
134.31
133.93
128.54
126.04
126.00
125.97
125.93
48.01
25.66
23.59
HN
S
O
N
CF3
2af
13C NMR
(100 MHz, CDCl3)
f1 (ppm)

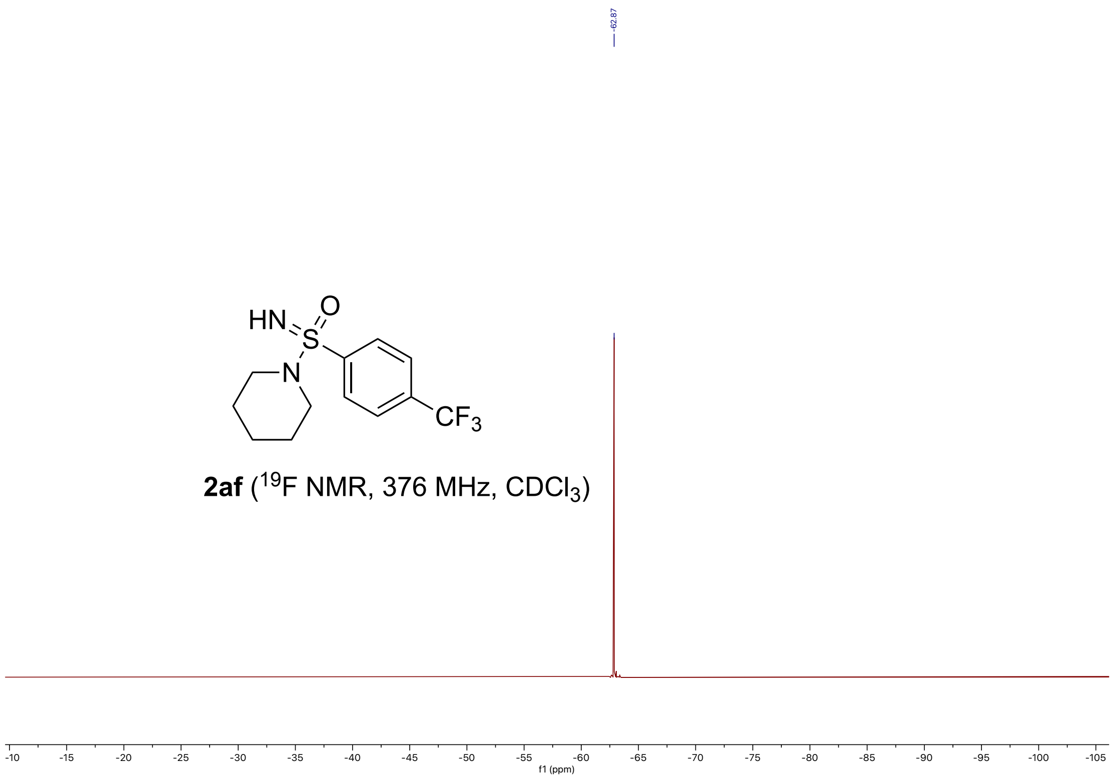

2af (^{19}F NMR, 376 MHz, $CDCl_3$)

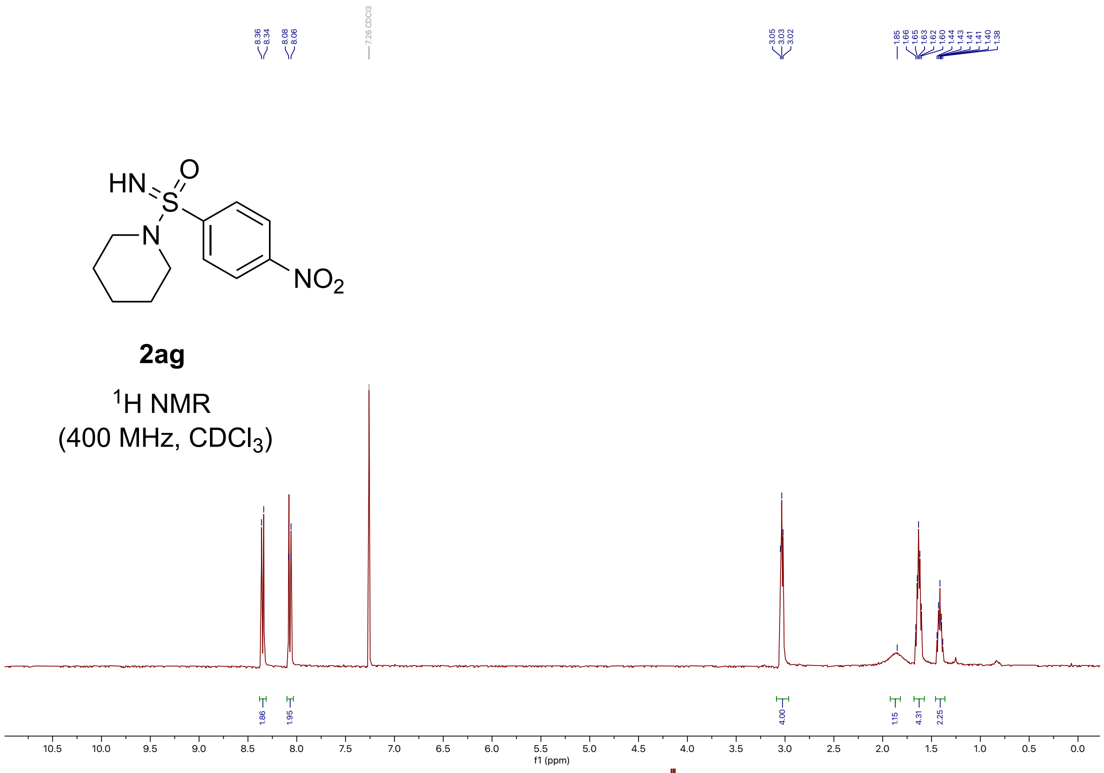
8.36
8.34
8.08
8.06
7.26 CDCl3
3.05
3.03
3.02
1.85
1.66
1.65
1.63
1.62
1.60
1.44
1.43
1.41
1.41
1.40
1.38
HN
O
S
N
NO2
2ag
1H NMR
(400 MHz, CDCl3)
1.86
1.95
4.00
1.15
4.31
2.25
f1 (ppm)

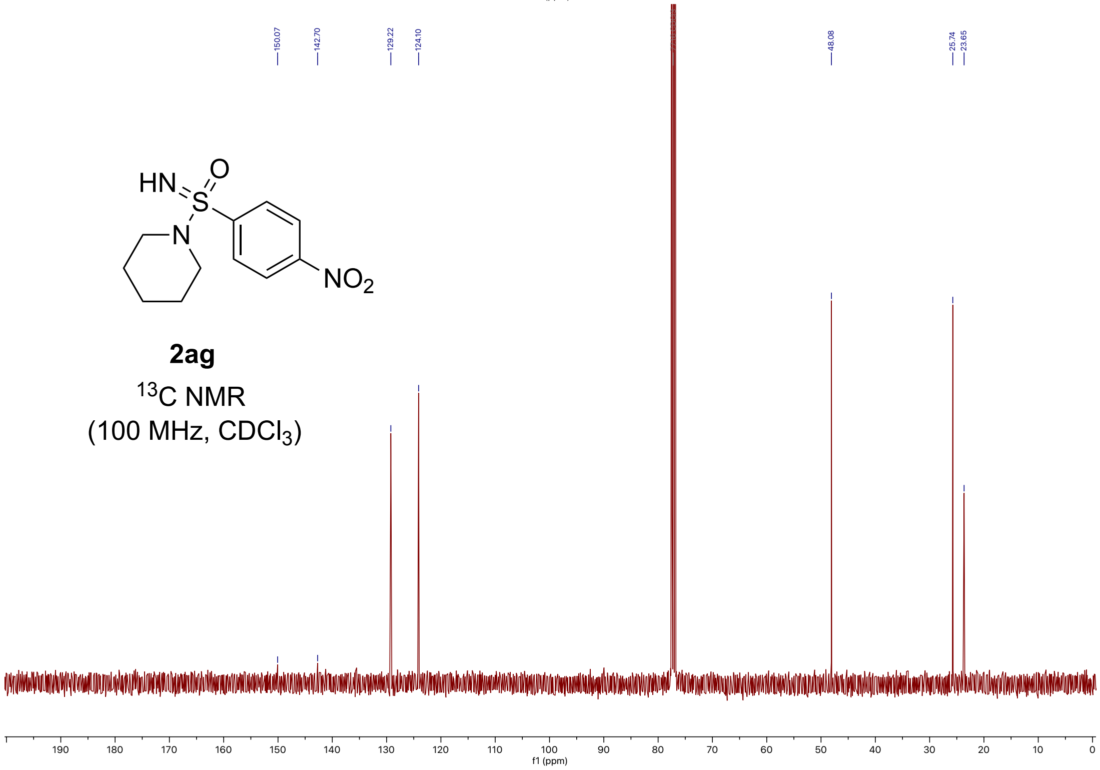
150.07
142.70
129.22
124.10
48.08
25.74
23.65
HN
O
S
N
NO2
2ag
13C NMR
(100 MHz, CDCl3)
f1 (ppm)

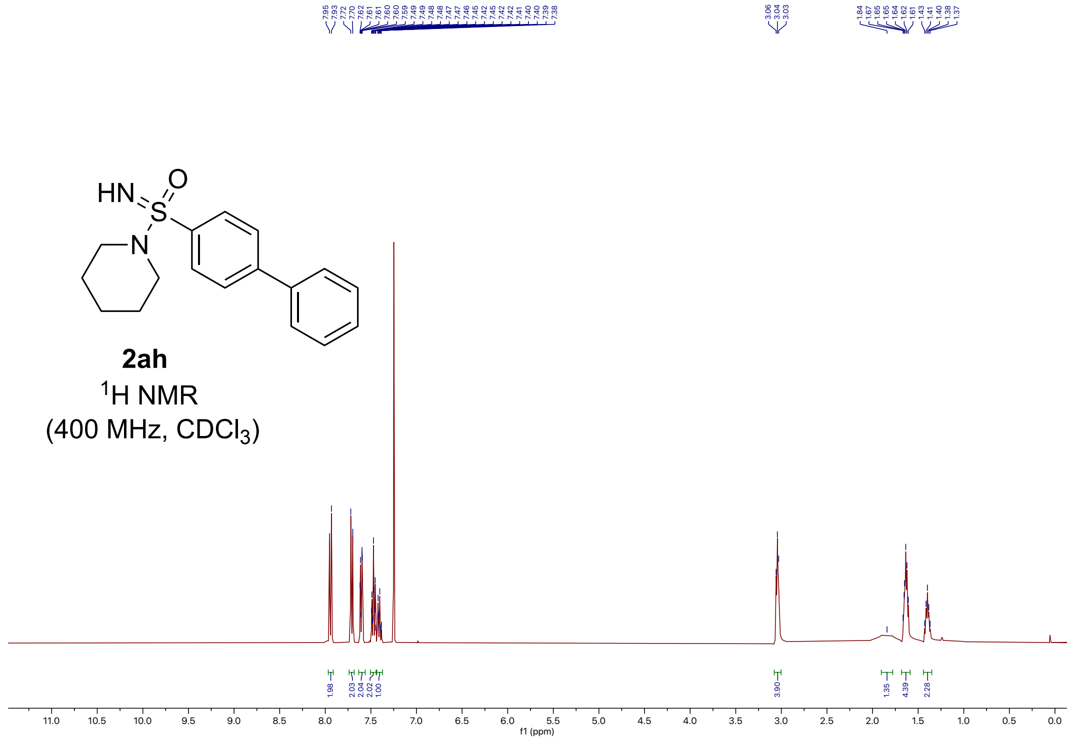
HN
O
S
N
2ah
1H NMR
(400 MHz, CDCl3)
f1 (ppm)

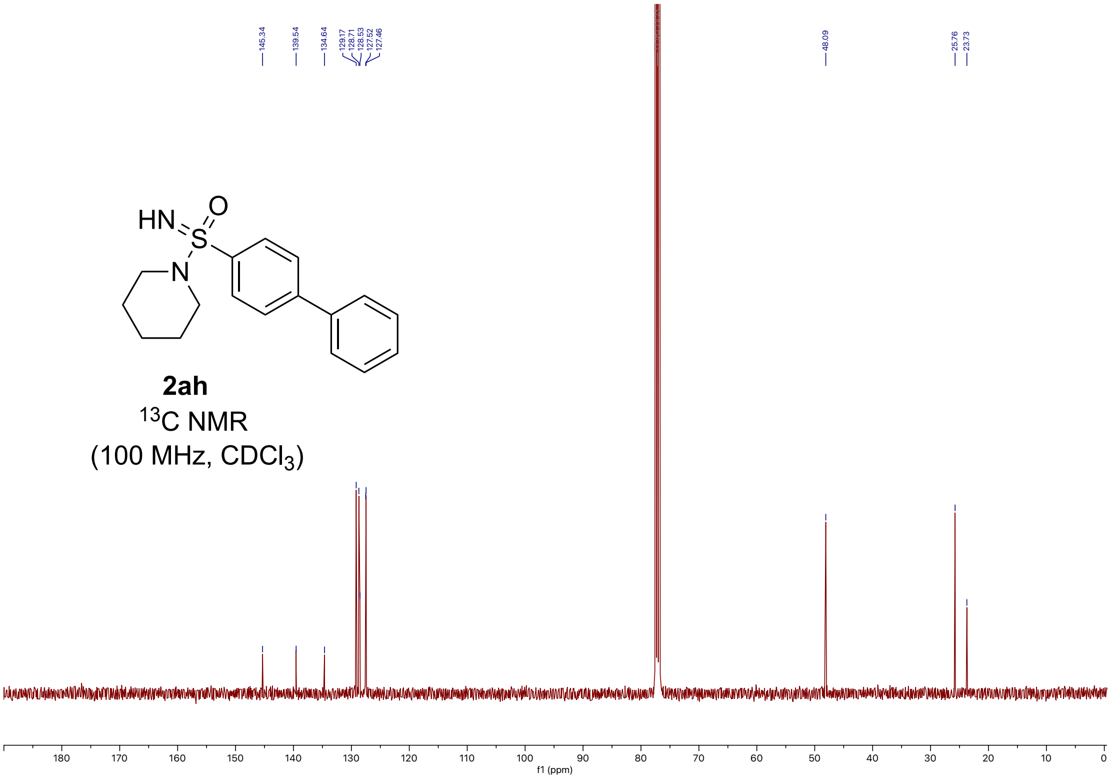
145.34
139.54
134.64
129.17
128.71
128.53
127.52
127.46
48.09
25.76
23.73
HN
O
S
N
2ah
13C NMR
(100 MHz, CDCl3)
f1 (ppm)

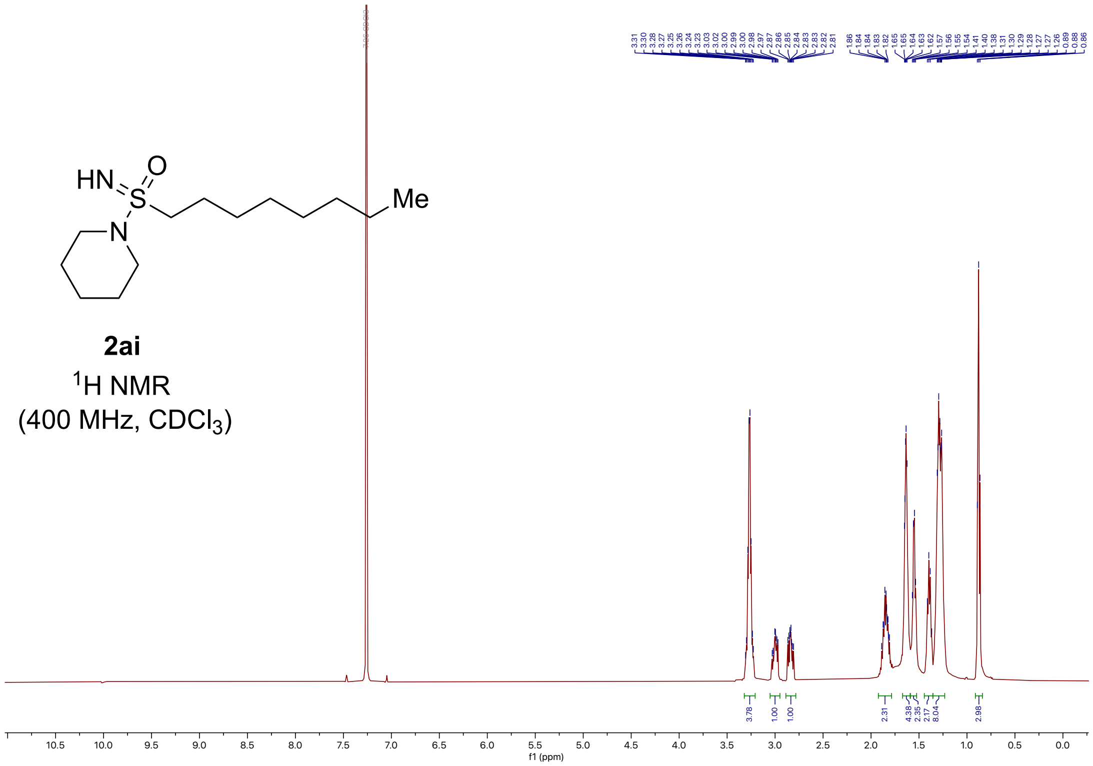
HN
O
S
N
Me
2ai
1H NMR
(400 MHz, CDCl3)
f1 (ppm)

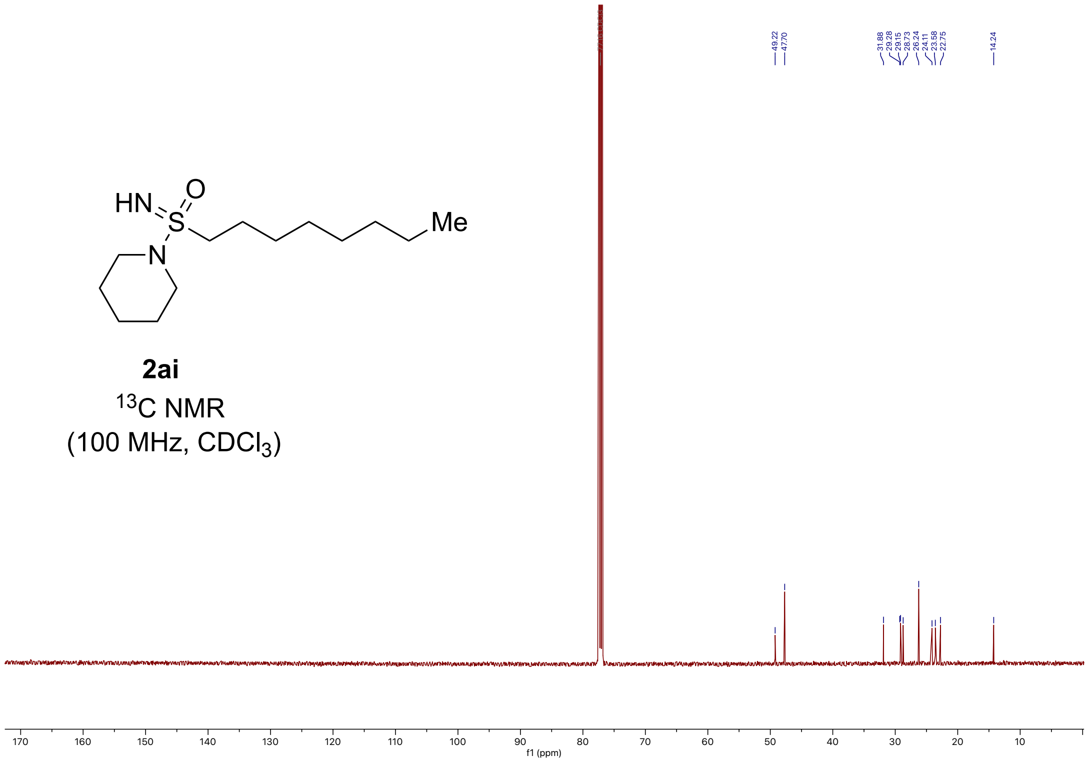
HN
O
S
N
Me
2ai
13C NMR
(100 MHz, CDCl3)
49.22
47.70
31.88
29.28
29.15
28.73
26.24
24.11
23.58
22.75
14.24
f1 (ppm)

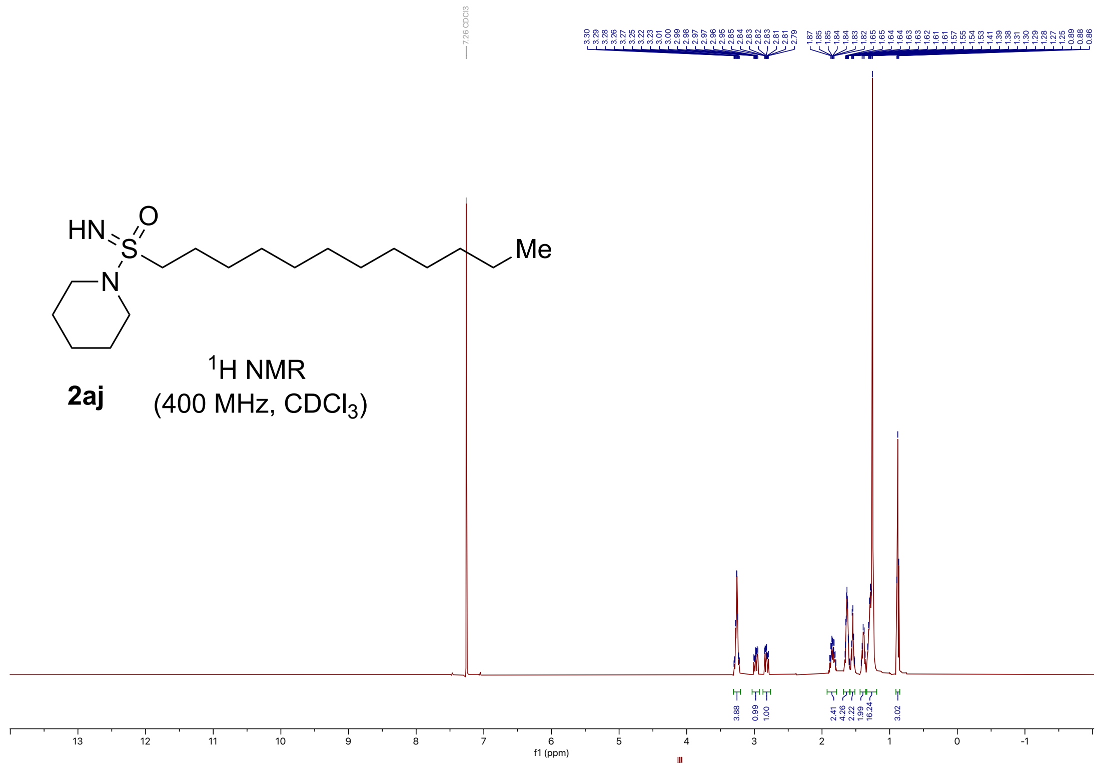
HN
O
S
N
Me
2aj
^{1}H NMR
(400 MHz, $CDCl_3$)
f1 (ppm)

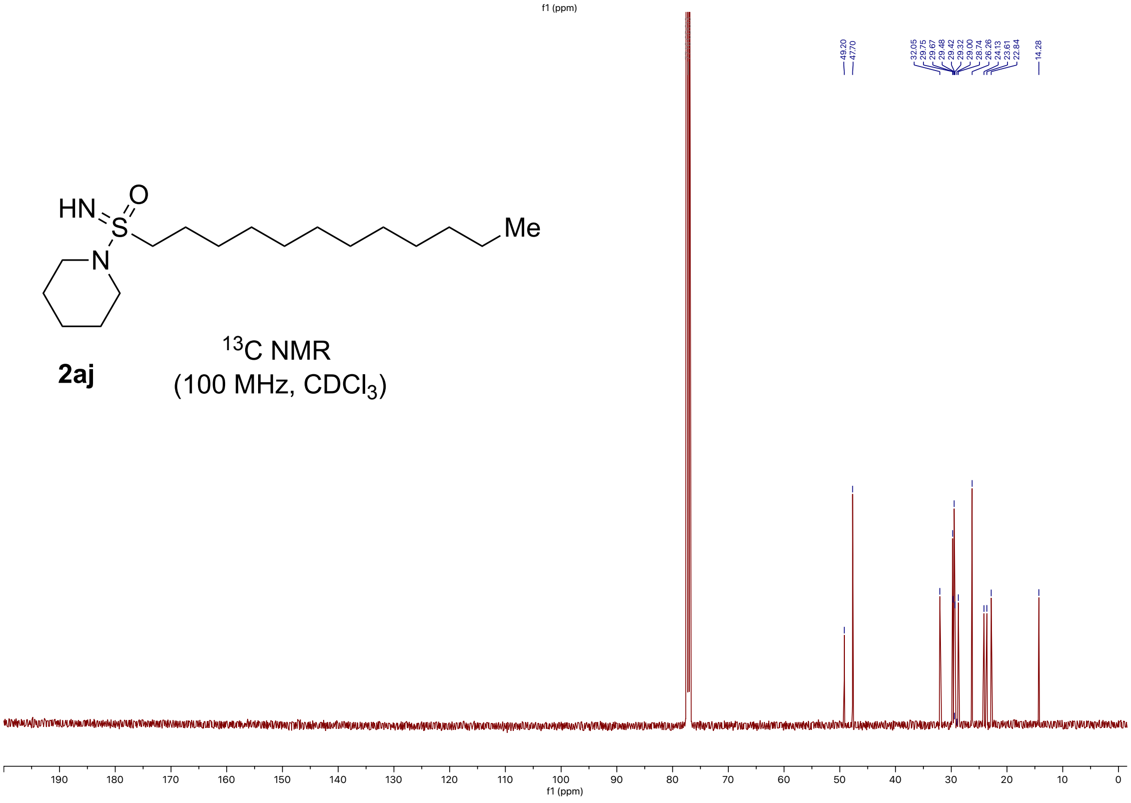
HN
O
S
N
Me
2aj
^{13}C NMR
(100 MHz, $CDCl_3$)
f1 (ppm)

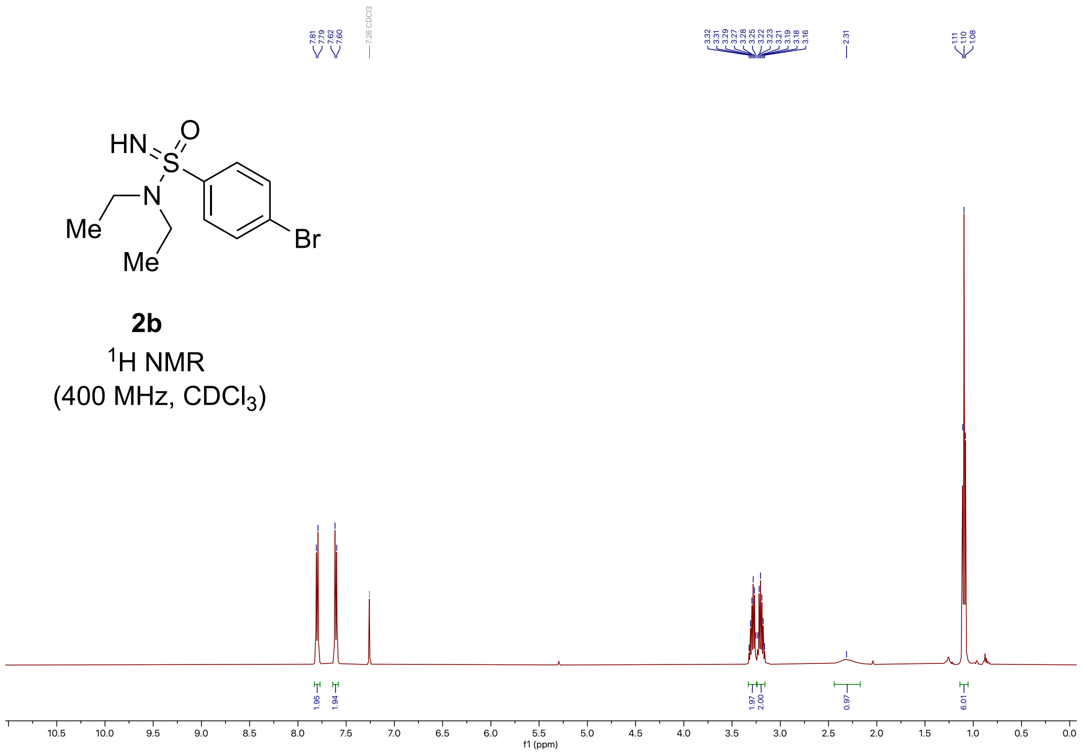
HN
O
S
N
Me
Me
Br
2b
1H NMR
(400 MHz, CDCl3)
f1 (ppm)

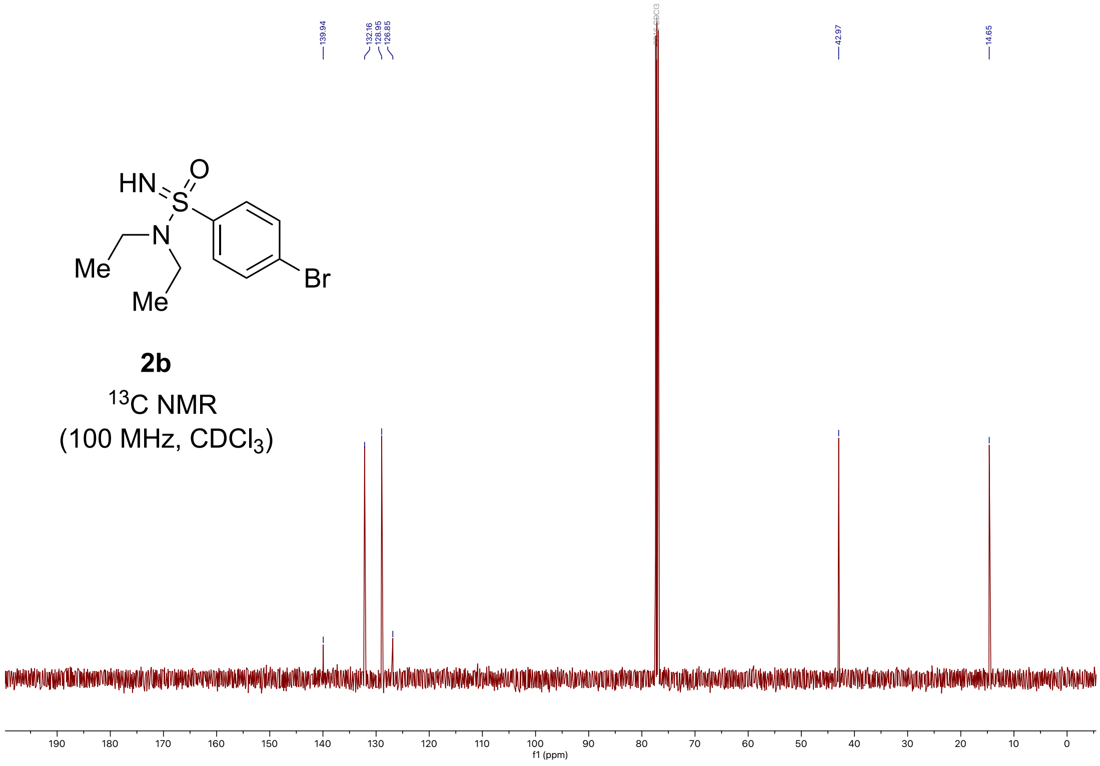
HN
O
S
N
Me
Me
Br
2b
13C NMR
(100 MHz, CDCl3)
f1 (ppm)

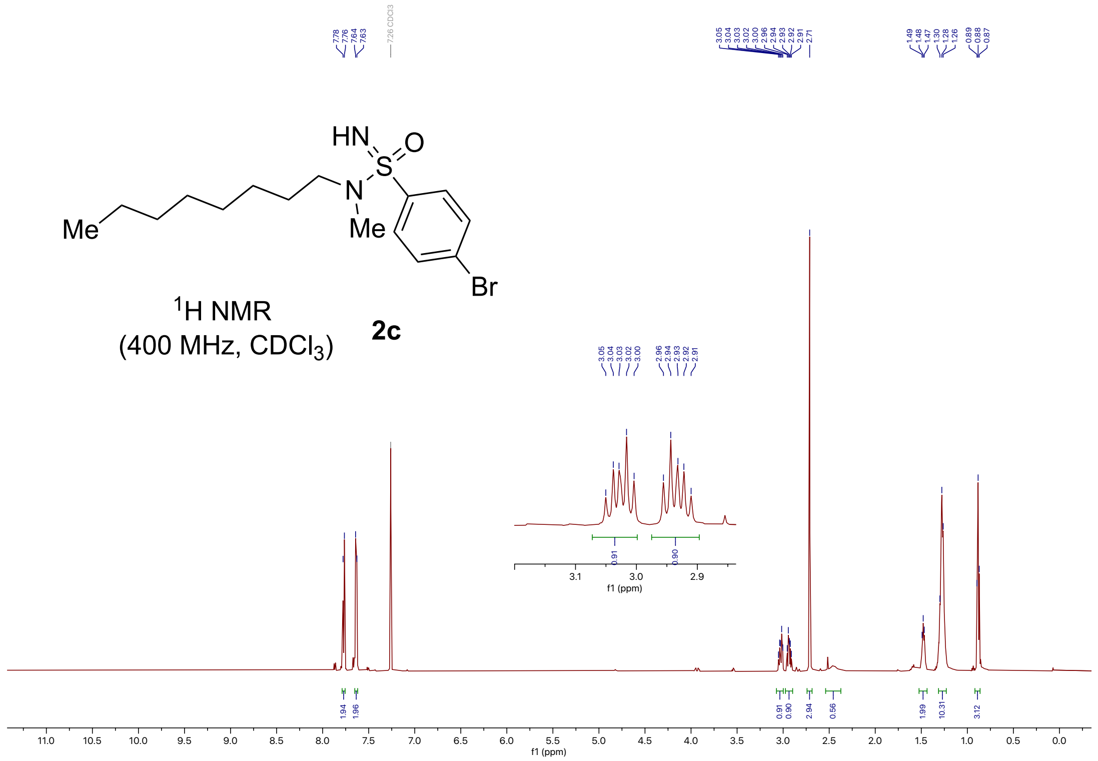

HN
O
S
N
Me
Me
Br
^{1}H NMR
(400 MHz, $CDCl_3$)
2c
f1 (ppm)

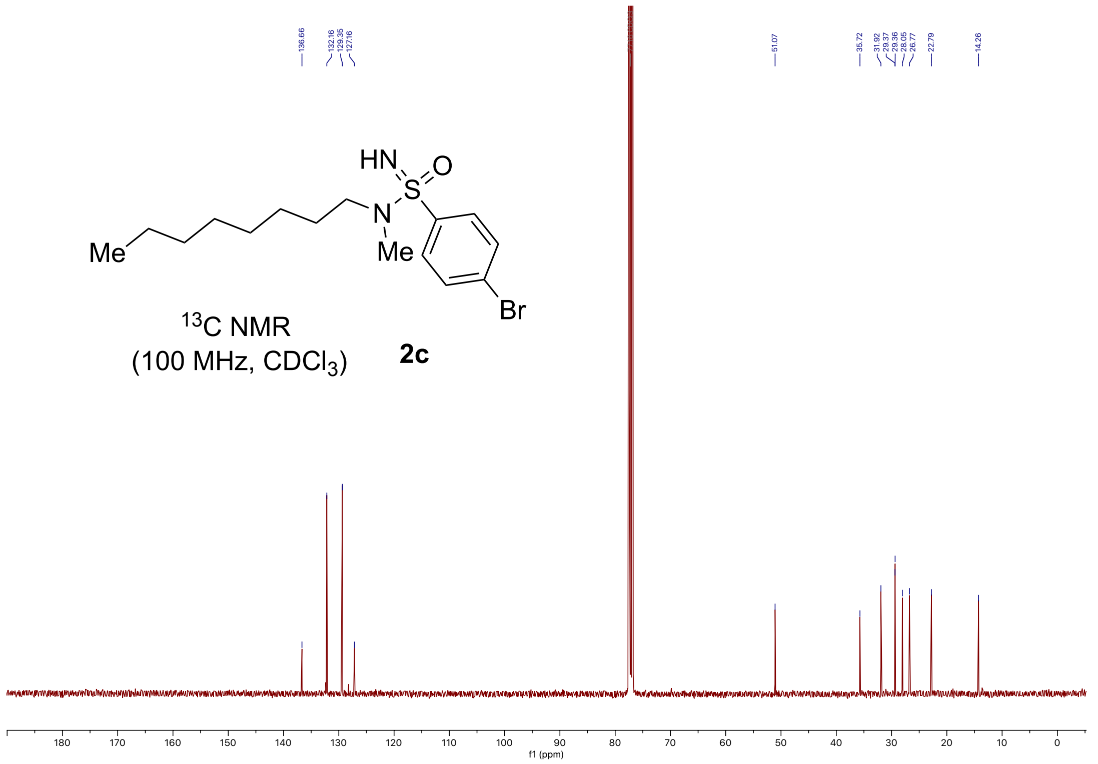

HN
O
S
N
Me
Me
Br
^{13}C NMR
(100 MHz, $CDCl_3$)
2c
f1 (ppm)

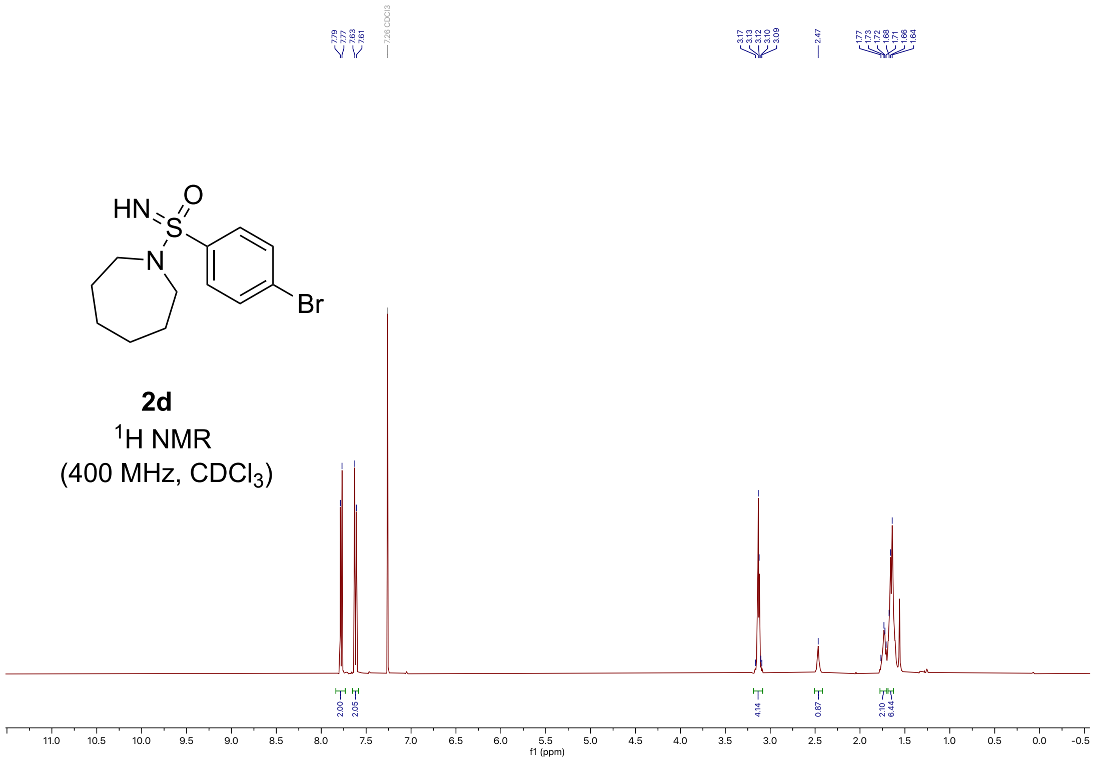

2d
^{1}H NMR
(400 MHz, CDCl$_3$)

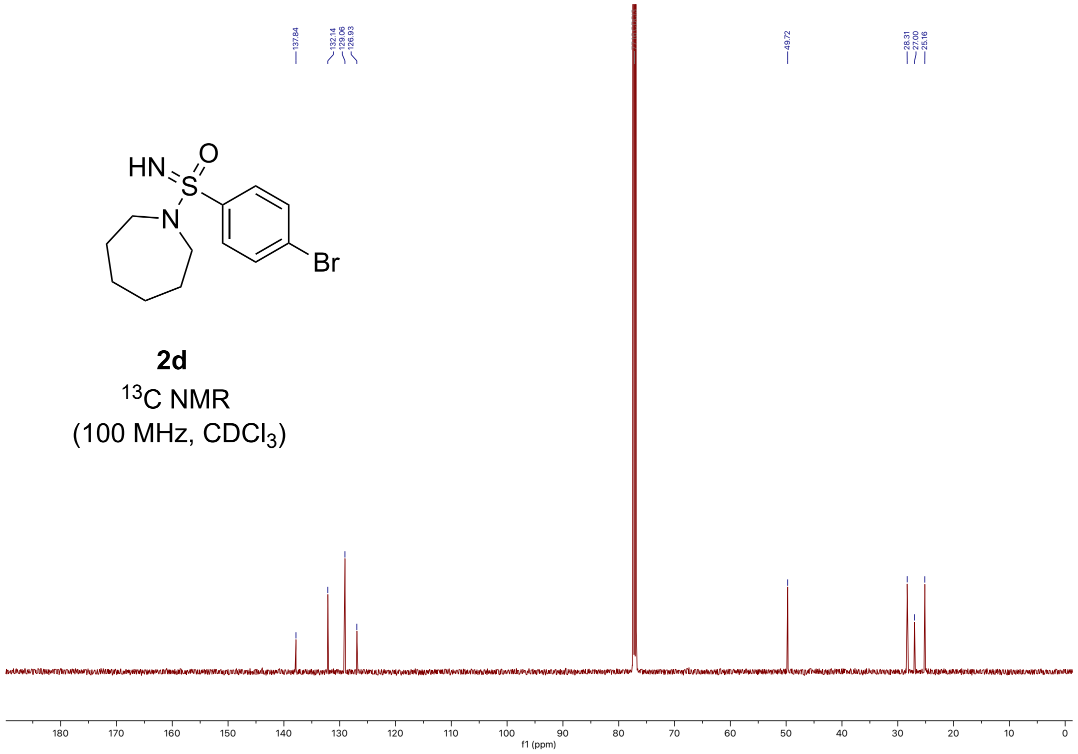

2d
^{13}C NMR
(100 MHz, CDCl$_3$)

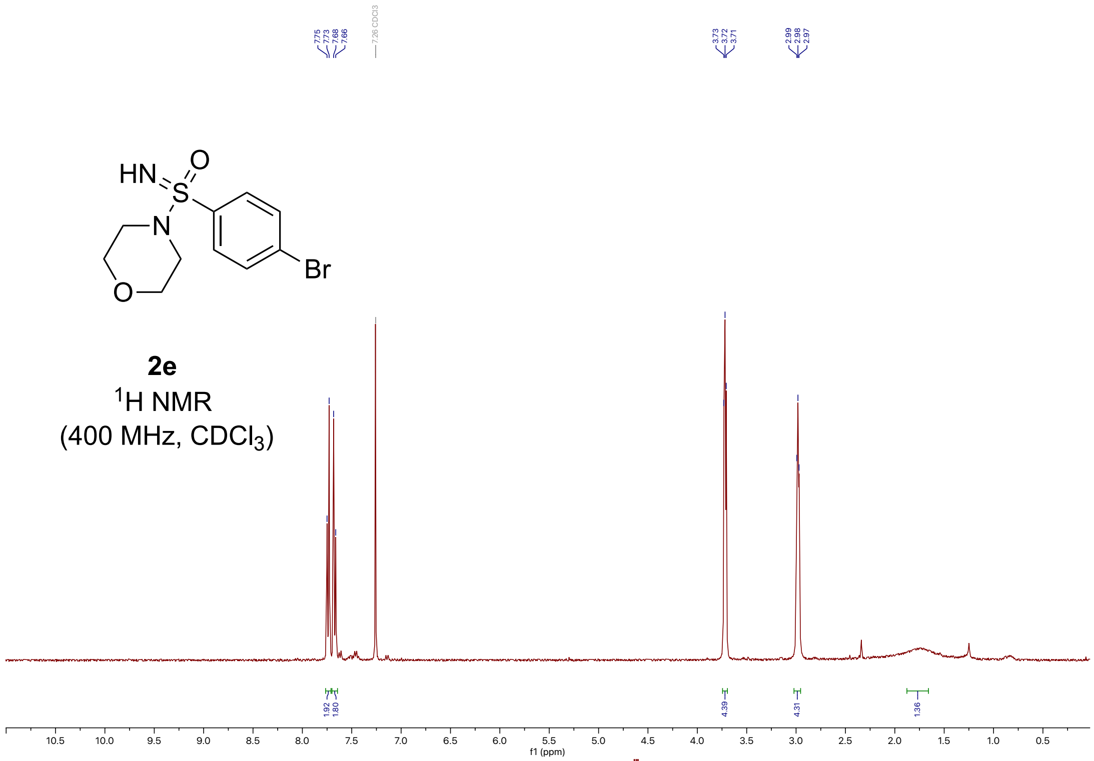
HN
O
S
N
O
Br
2e
1H NMR
(400 MHz, CDCl3)
f1 (ppm)

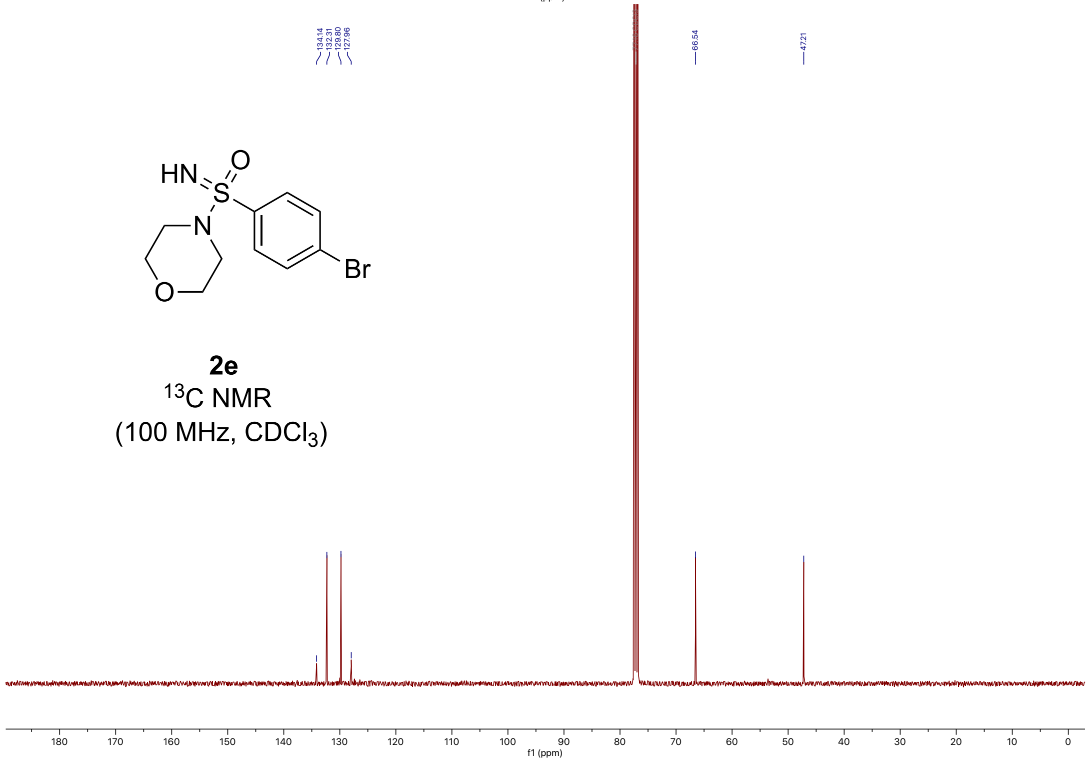
HN
O
S
N
O
Br
2e
13C NMR
(100 MHz, CDCl3)
f1 (ppm)

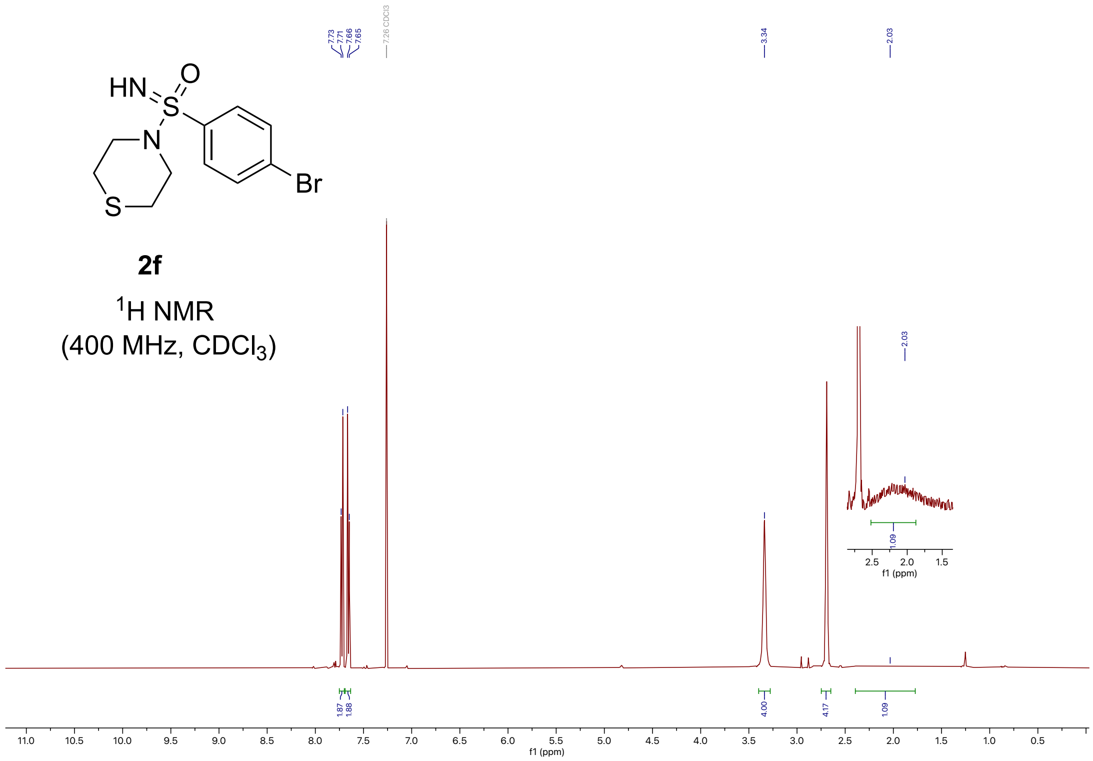
HN
O
S
N
S
Br
2f
1H NMR
(400 MHz, CDCl3)
7.73
7.71
7.66
7.65
7.26 CDCl3
3.34
2.03
1.87
1.88
4.00
4.17
1.09
f1 (ppm)

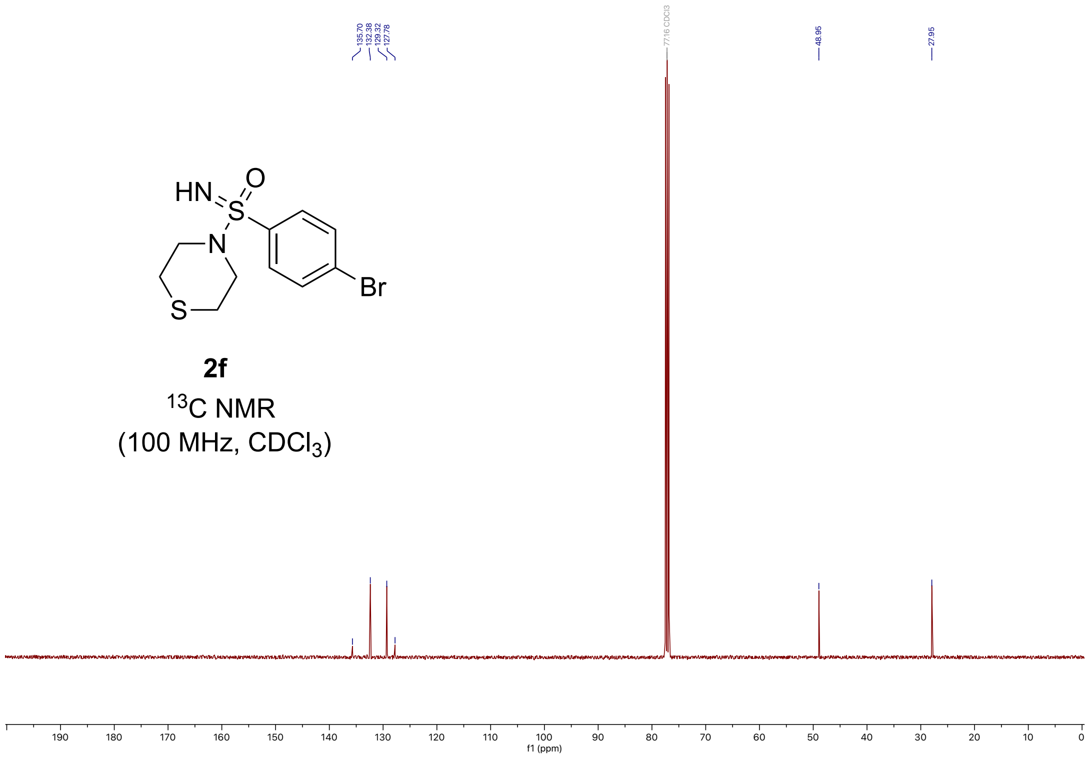
HN
O
S
N
S
Br
2f
13C NMR
(100 MHz, CDCl3)
135.70
132.38
129.32
127.78
77.16 CDCl3
48.95
27.95
f1 (ppm)

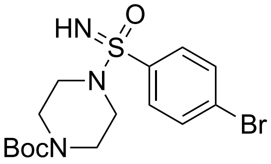

2g
^{1}H NMR
(400 MHz, $CDCl_3$)

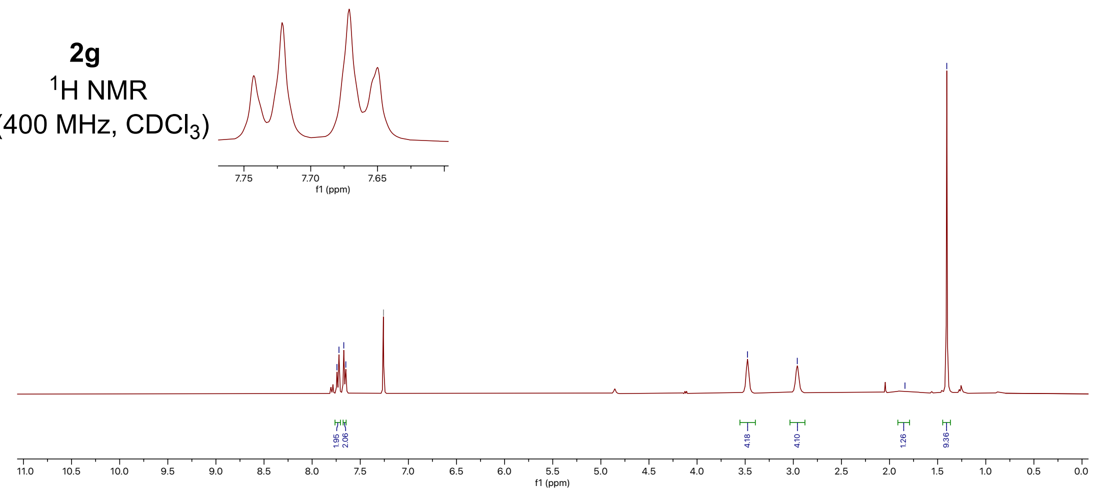

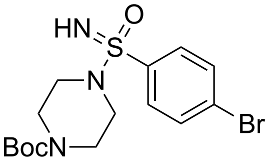

2g
^{13}C NMR
(100 MHz, $CDCl_3$)

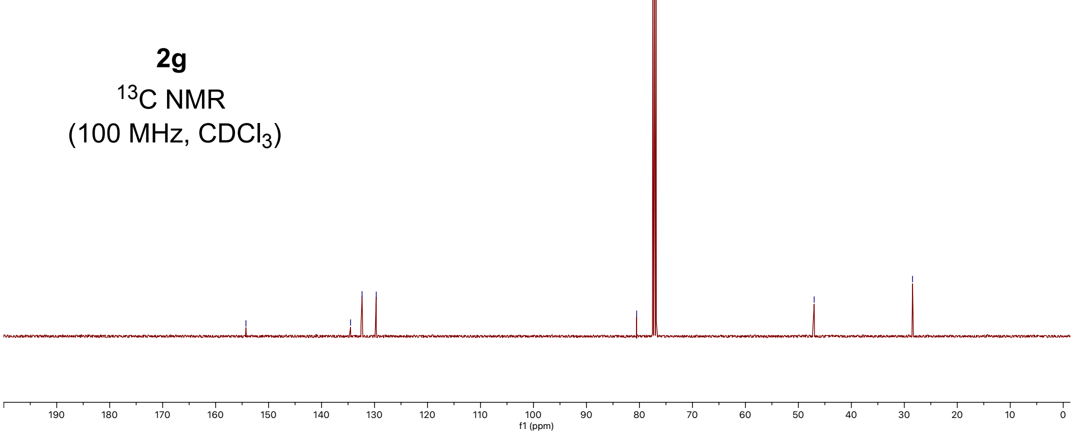

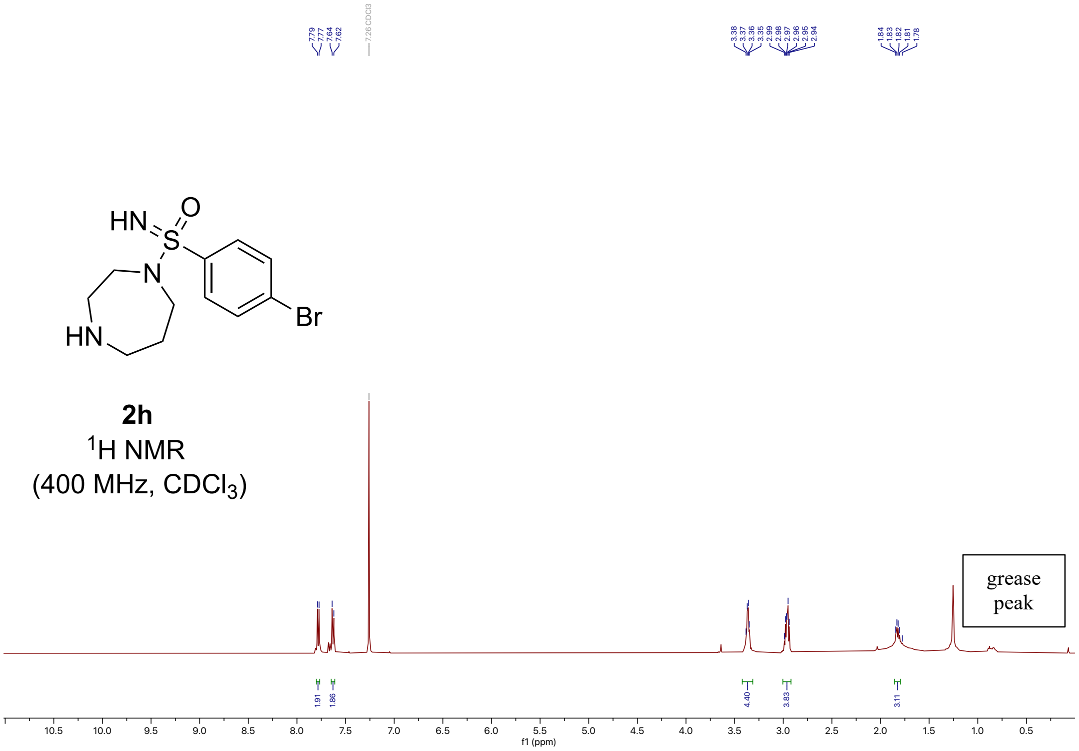

HN
O
S
N
HN
Br
2h
1H NMR
(400 MHz, CDCl3)
grease
peak
f1 (ppm)

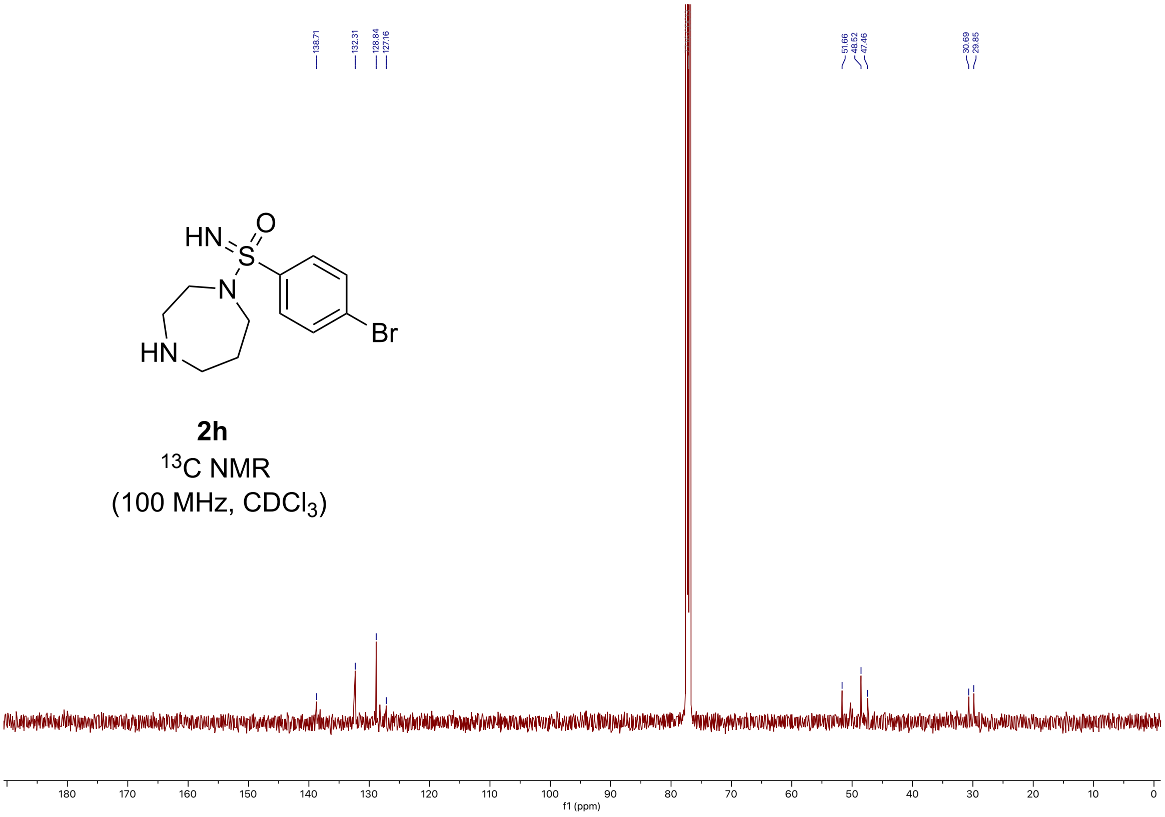

HN
O
S
N
HN
Br
2h
13C NMR
(100 MHz, CDCl3)
138.71
132.31
128.84
127.16
51.66
48.52
47.46
30.69
29.85
f1 (ppm)

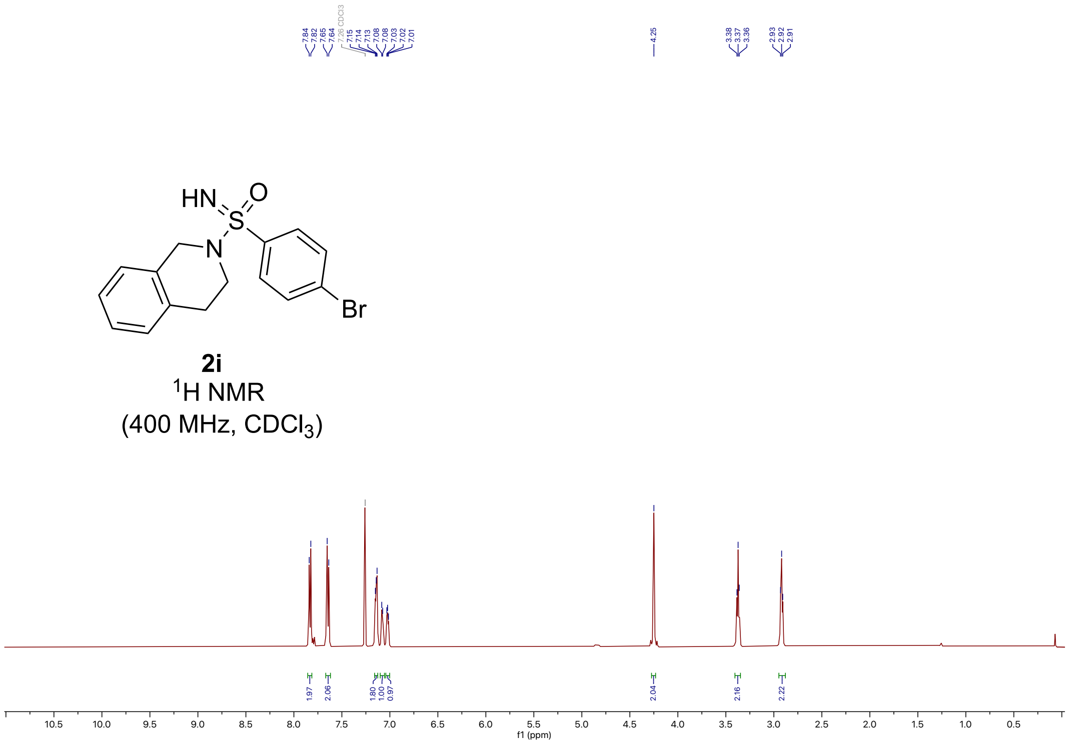
7.84
7.82
7.65
7.64
7.26 CDCl3
7.15
7.14
7.13
7.08
7.08
7.03
7.02
7.01
4.25
3.38
3.37
3.36
2.93
2.92
2.91
HN
O
S
N
Br
2i
1H NMR
(400 MHz, CDCl3)
1.97
2.06
1.80
1.00
0.97
2.04
2.16
2.22
10.5
10.0
9.5
9.0
8.5
8.0
7.5
7.0
6.5
6.0
5.5
5.0
4.5
4.0
3.5
3.0
2.5
2.0
1.5
1.0
0.5
f1 (ppm)

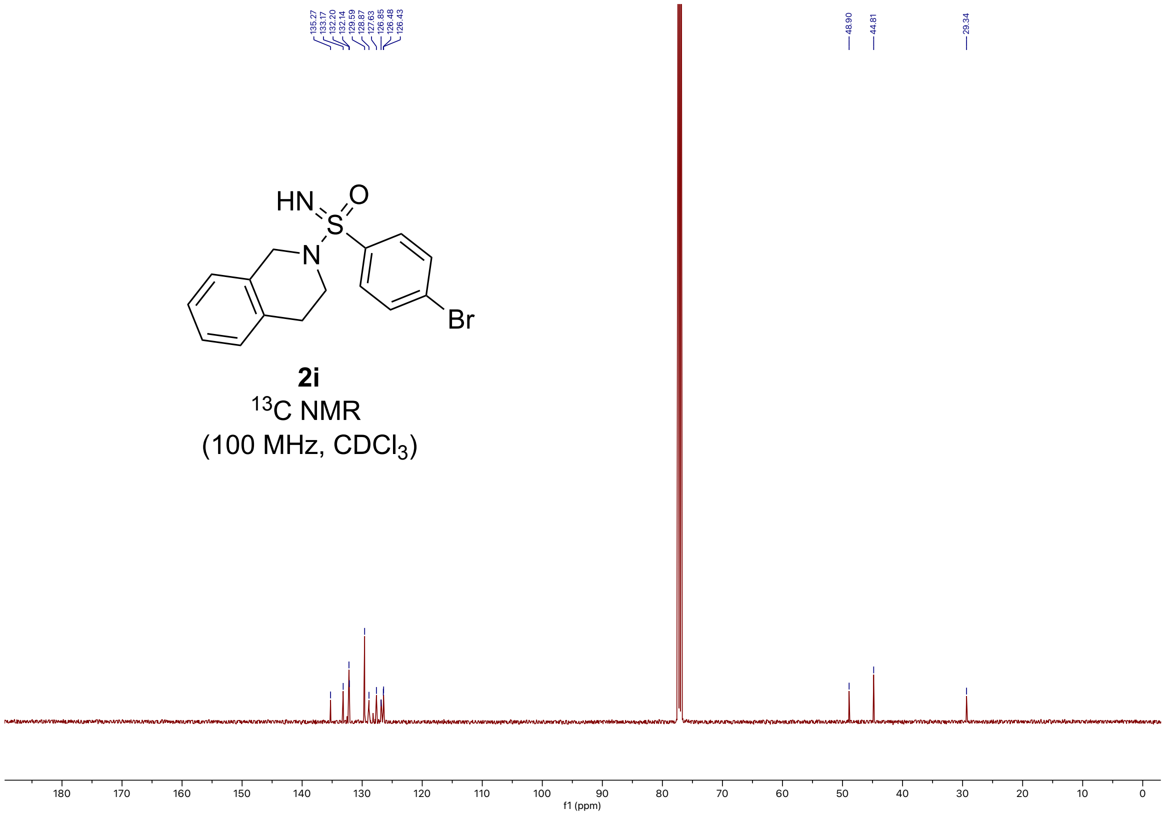
135.27
133.17
132.20
132.14
129.59
128.87
127.63
126.85
126.48
126.43
48.90
44.81
29.34
HN
O
S
N
Br
2i
13C NMR
(100 MHz, CDCl3)
180
170
160
150
140
130
120
110
100
90
80
70
60
50
40
30
20
10
0
f1 (ppm)

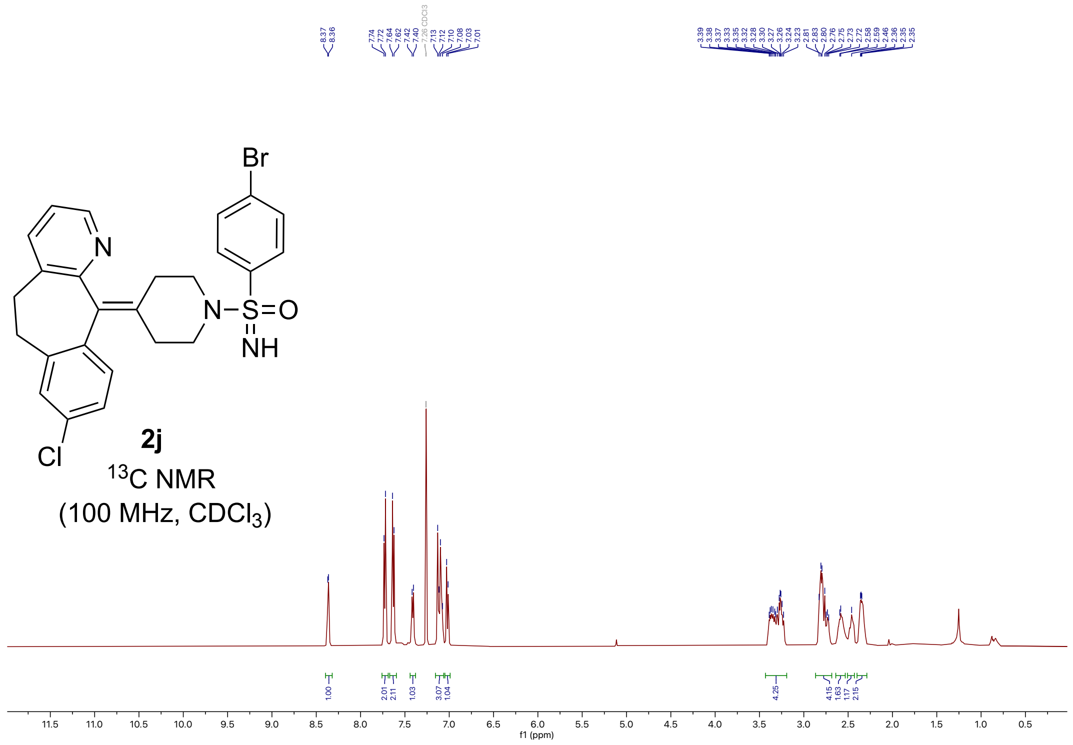
2j
^{13}C NMR
(100 MHz, CDCl$_3$)
f1 (ppm)

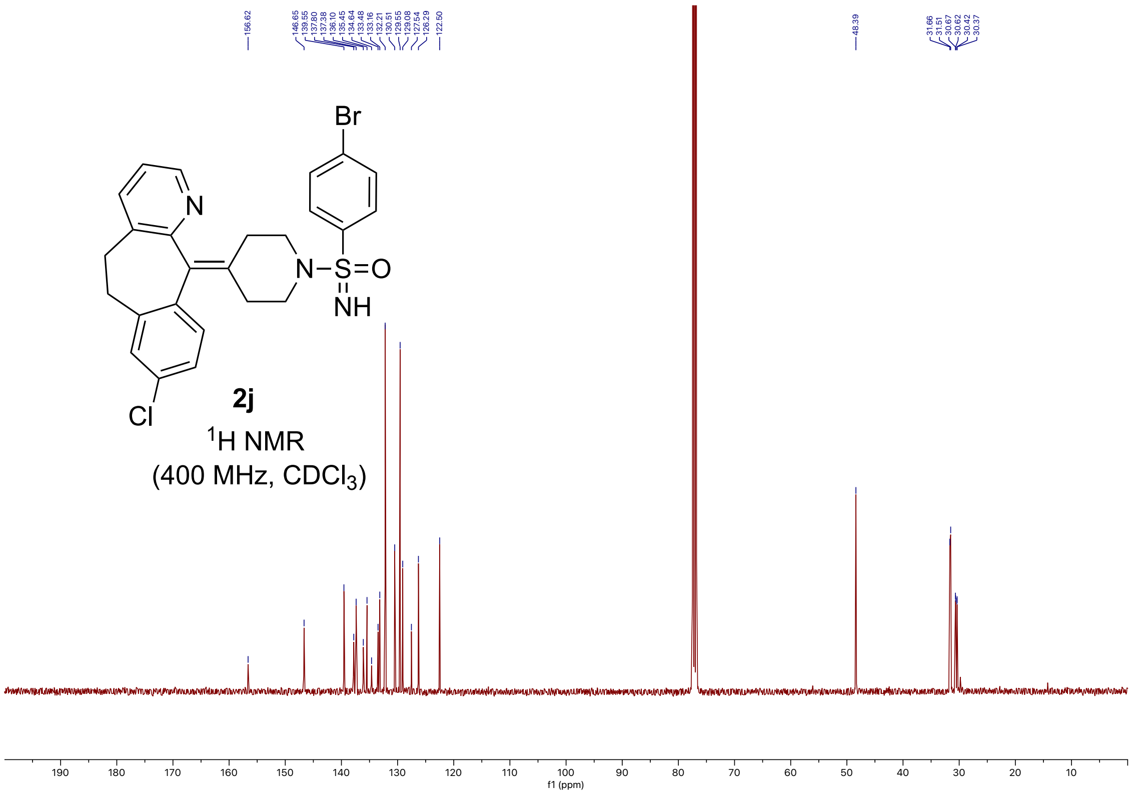
2j
^{1}H NMR
(400 MHz, CDCl$_3$)
f1 (ppm)

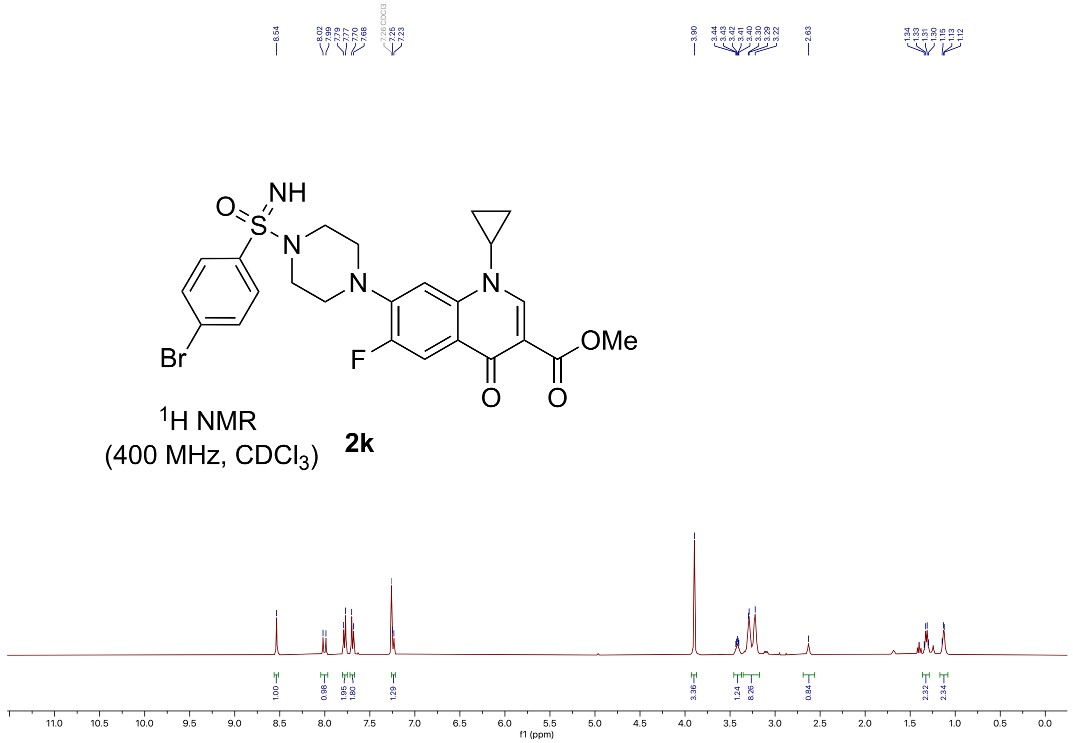

^{1}H NMR
(400 MHz, $CDCl_3$)
2k
f1 (ppm)

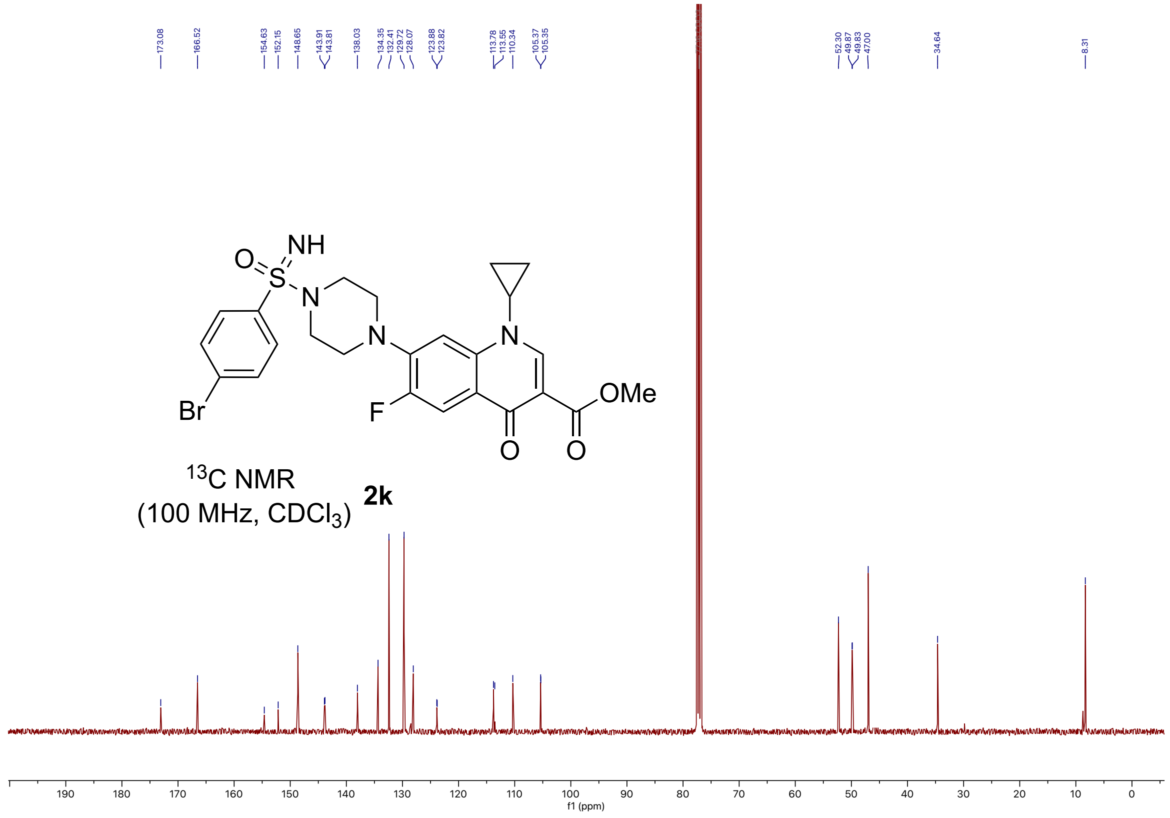

^{13}C NMR
(100 MHz, $CDCl_3$)
2k
f1 (ppm)

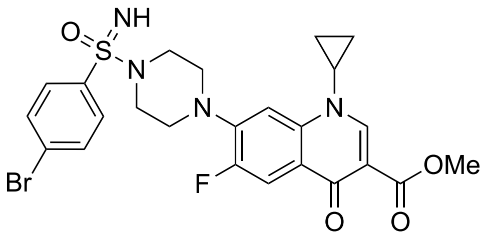

2k (^{19}F NMR, 376 MHz, $CDCl_3$)

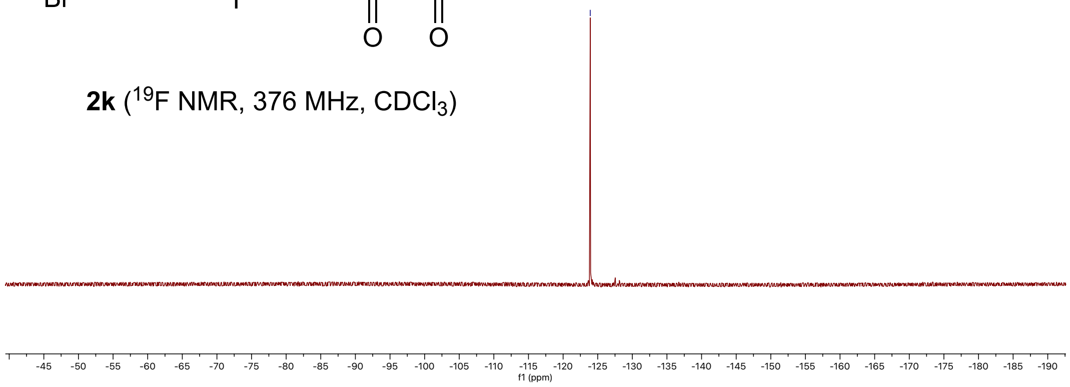

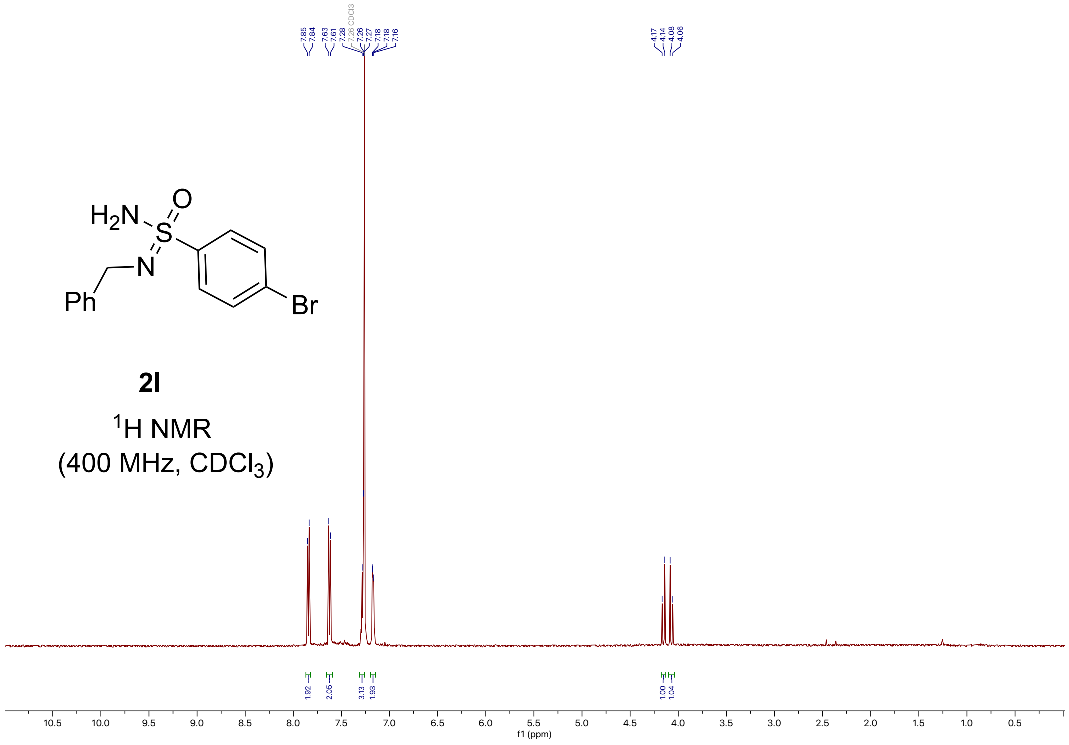
7.85
7.84
7.63
7.61
7.28
7.26 CDCl3
7.26
7.27
7.18
7.18
7.16
4.17
4.14
4.08
4.06
H2N
O
S
N
Ph
Br
2l
1H NMR
(400 MHz, CDCl3)
1.92
2.05
3.13
1.93
1.00
1.04
10.5
10.0
9.5
9.0
8.5
8.0
7.5
7.0
6.5
6.0
5.5
5.0
4.5
4.0
3.5
3.0
2.5
2.0
1.5
1.0
0.5
f1 (ppm)

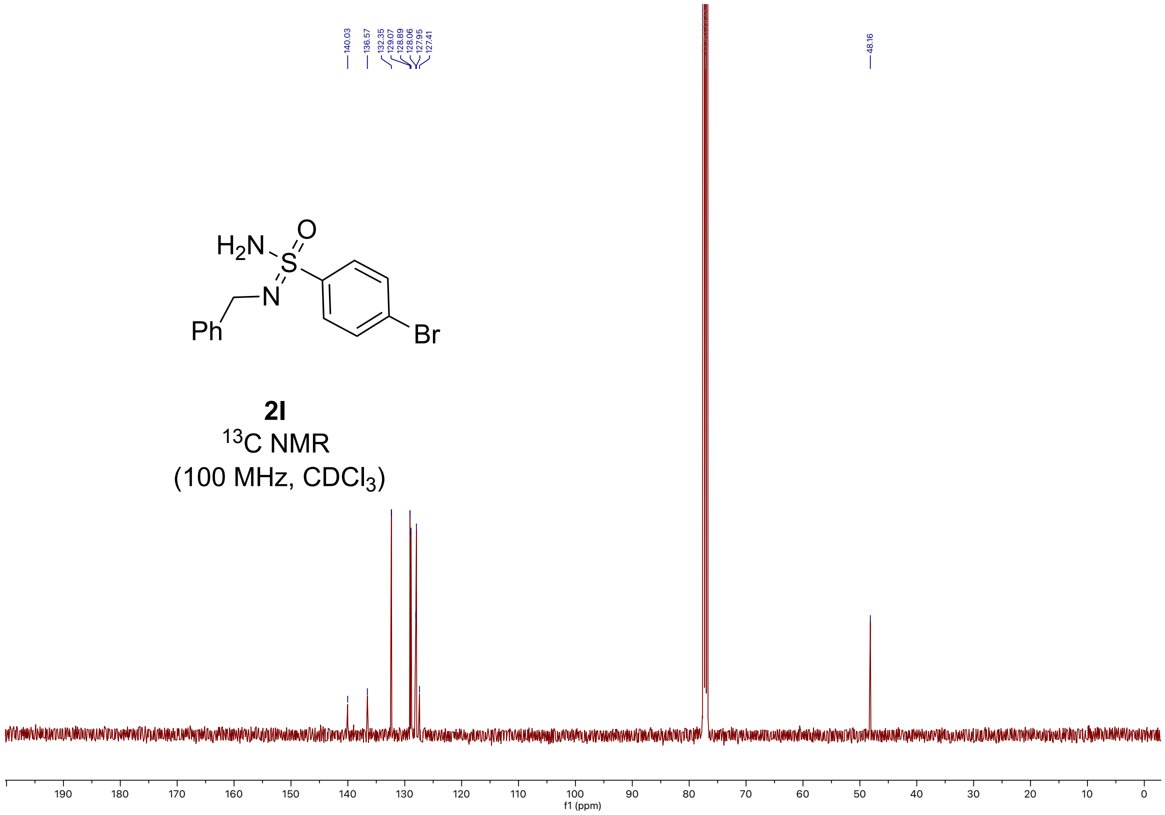
140.03
136.57
132.35
129.07
128.89
128.06
127.95
127.41
48.16
H2N
O
S
N
Ph
Br
2l
13C NMR
(100 MHz, CDCl3)
190
180
170
160
150
140
130
120
110
100
90
80
70
60
50
40
30
20
10
0
f1 (ppm)

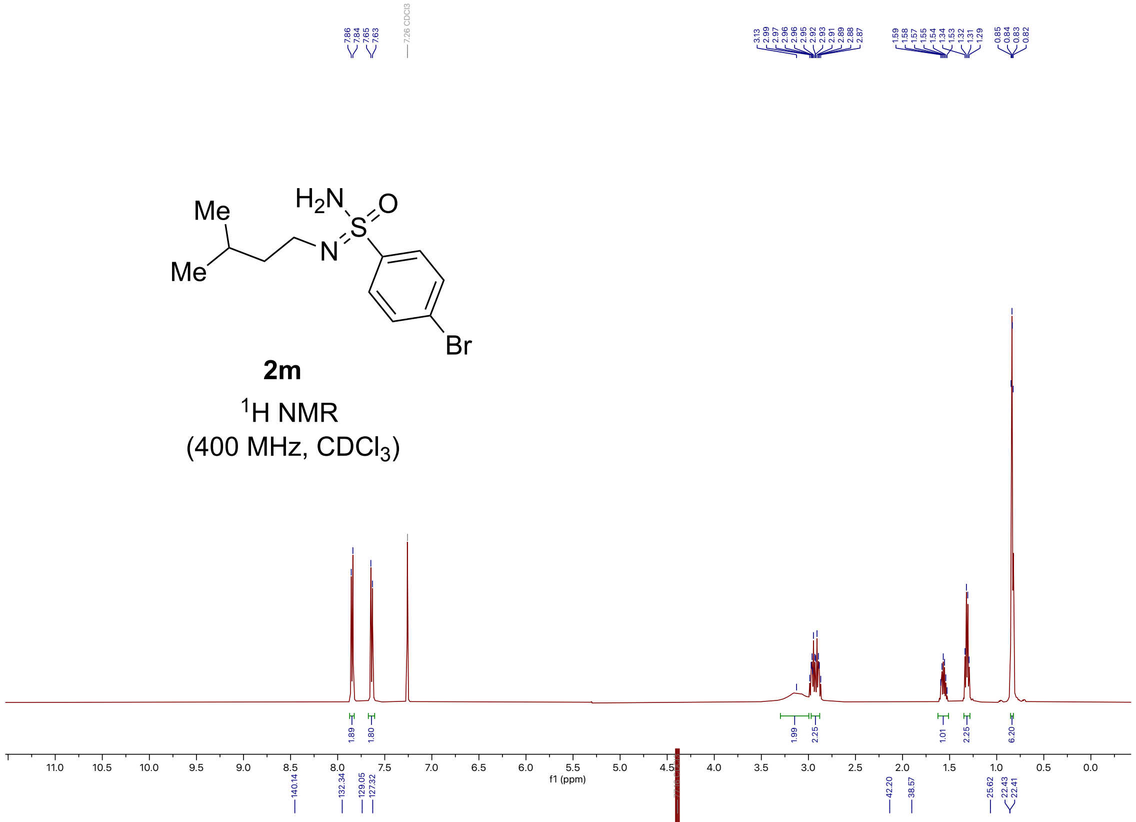

2m

^{1}H NMR

(400 MHz, $CDCl_3$)

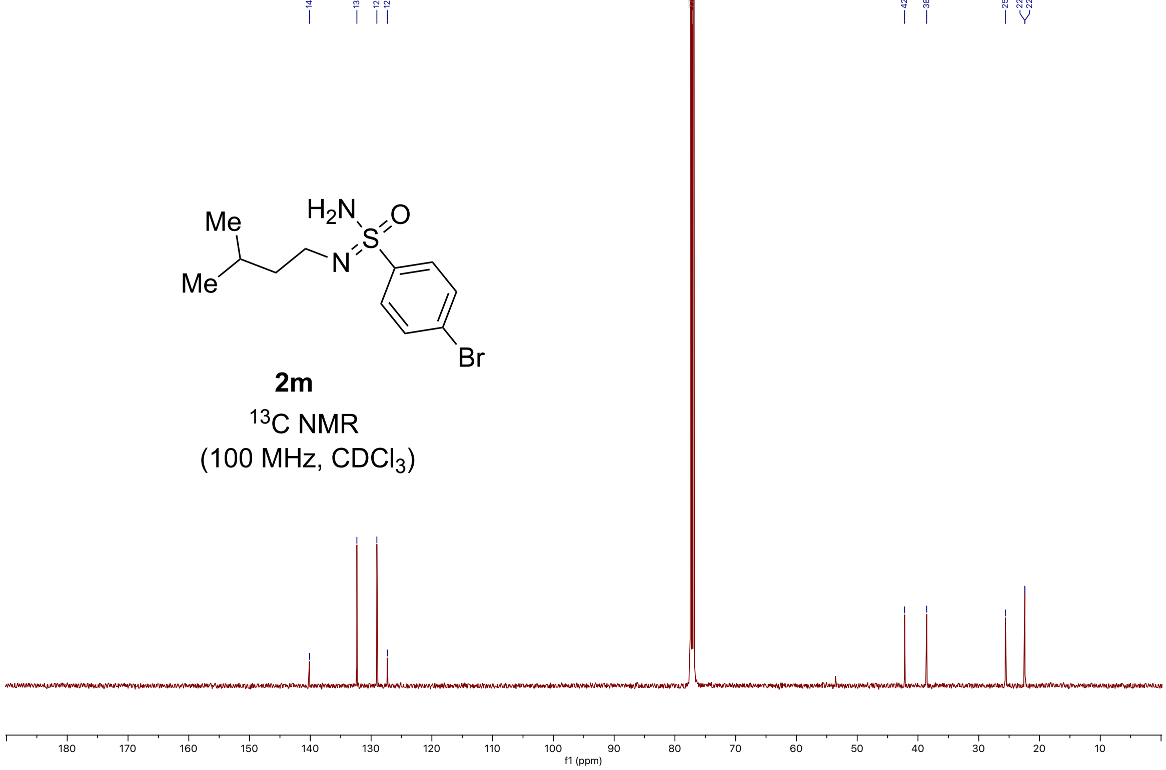

2m

^{13}C NMR

(100 MHz, $CDCl_3$)

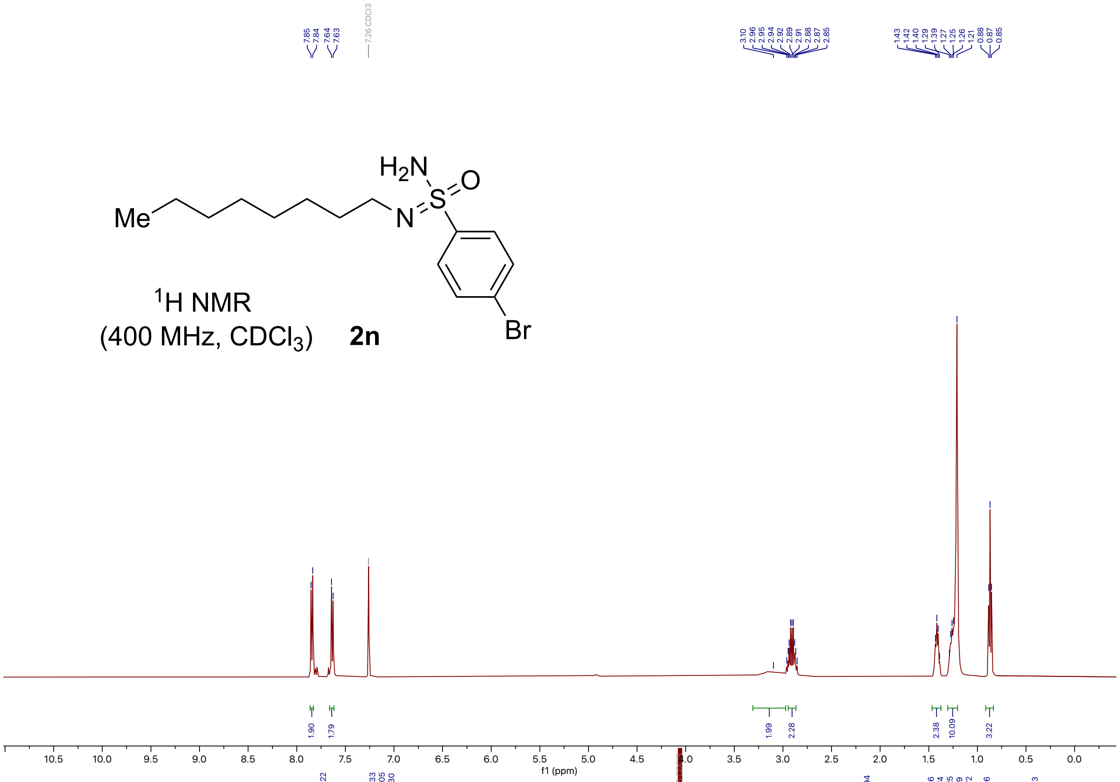
H2N
S
O
Me
N
Br
1H NMR
(400 MHz, CDCl3)
2n
f1 (ppm)

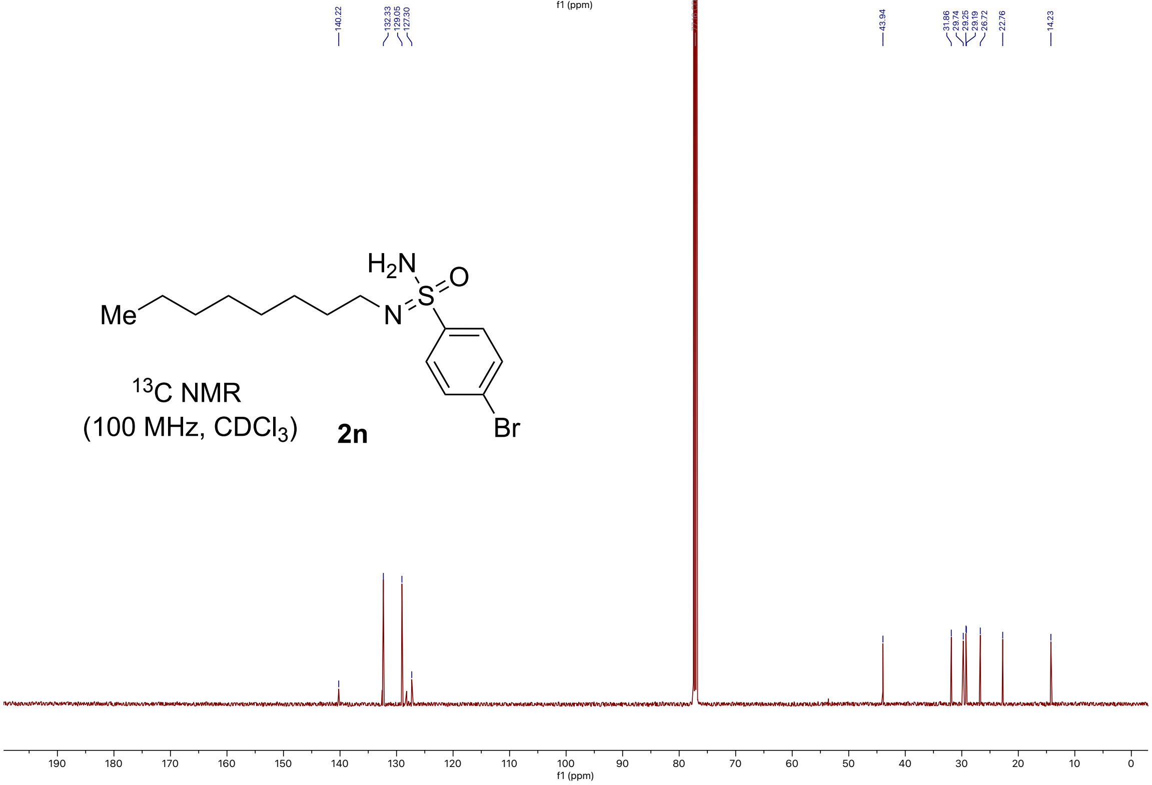
H2N
S
O
Me
N
Br
13C NMR
(100 MHz, CDCl3)
2n
f1 (ppm)

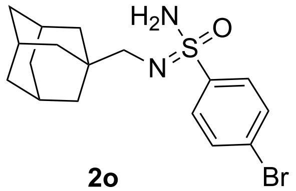

2o

^{1}H NMR
(400 MHz, $CDCl_3$)

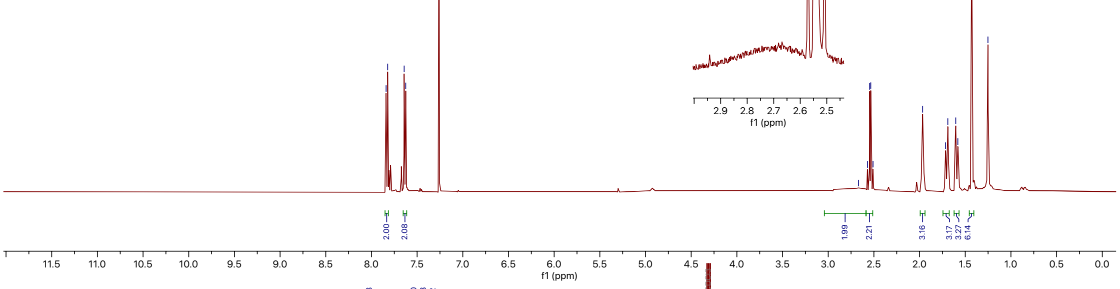

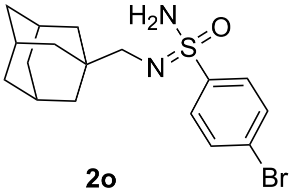

2o

^{13}C NMR
(100 MHz, $CDCl_3$)

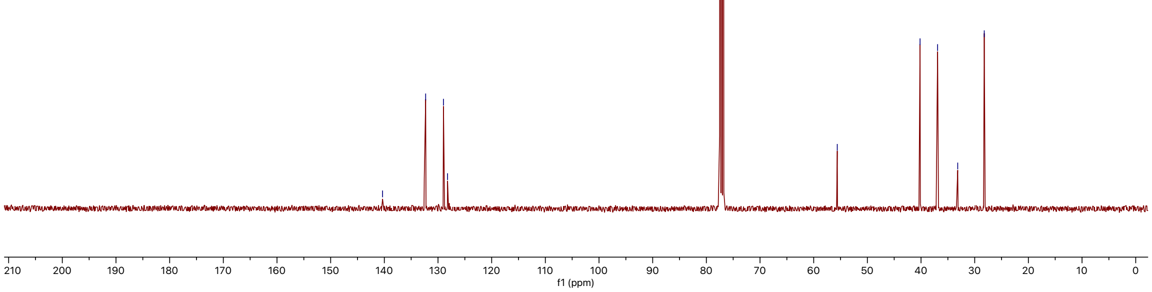

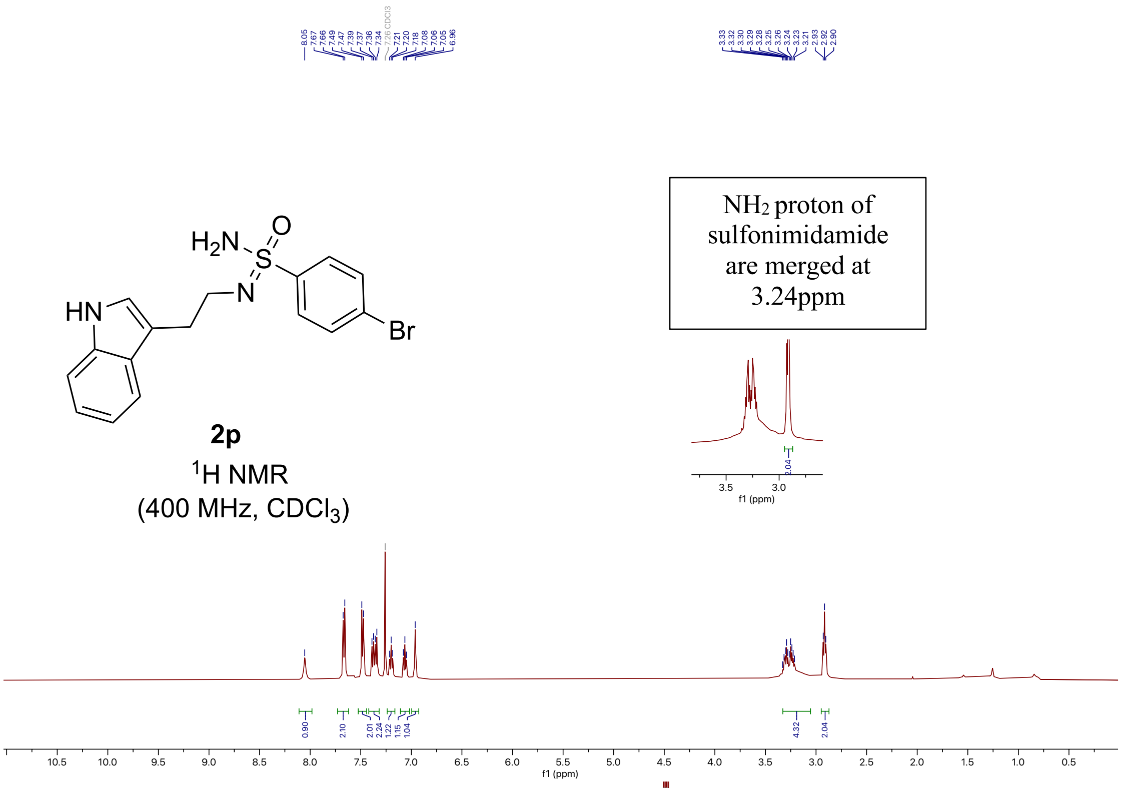

2p

^{1}H NMR (400 MHz, $CDCl_3$)

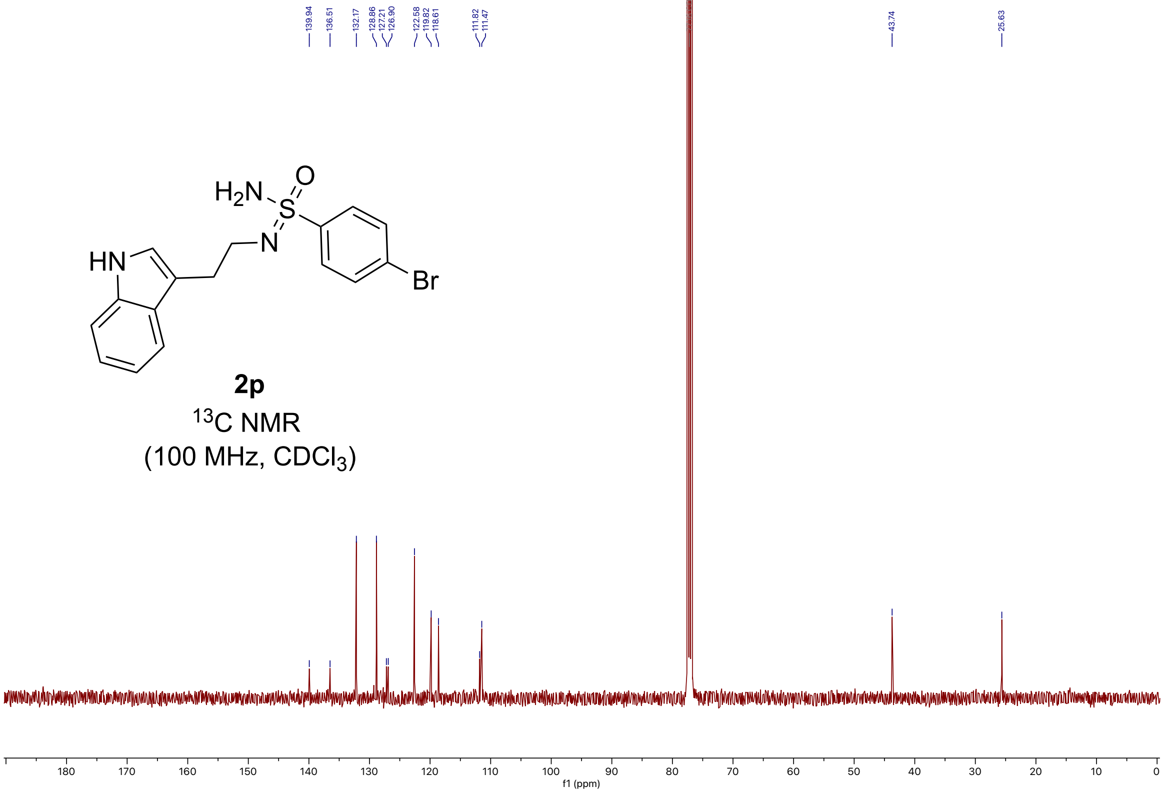

2p

^{13}C NMR (100 MHz, $CDCl_3$)

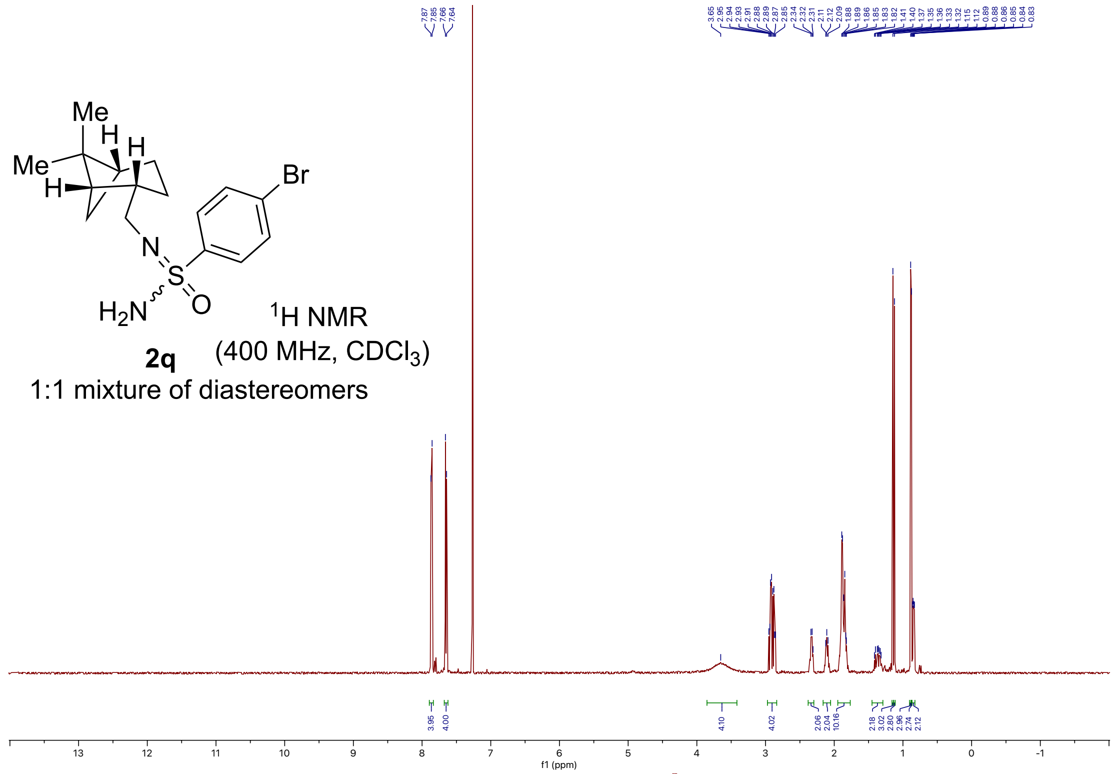
Me
H
H
Me
H
Br
N
S
H_2N
O
2q
1:1 mixture of diastereomers
^{1}H NMR
(400 MHz, $CDCl_3$)
f1 (ppm)

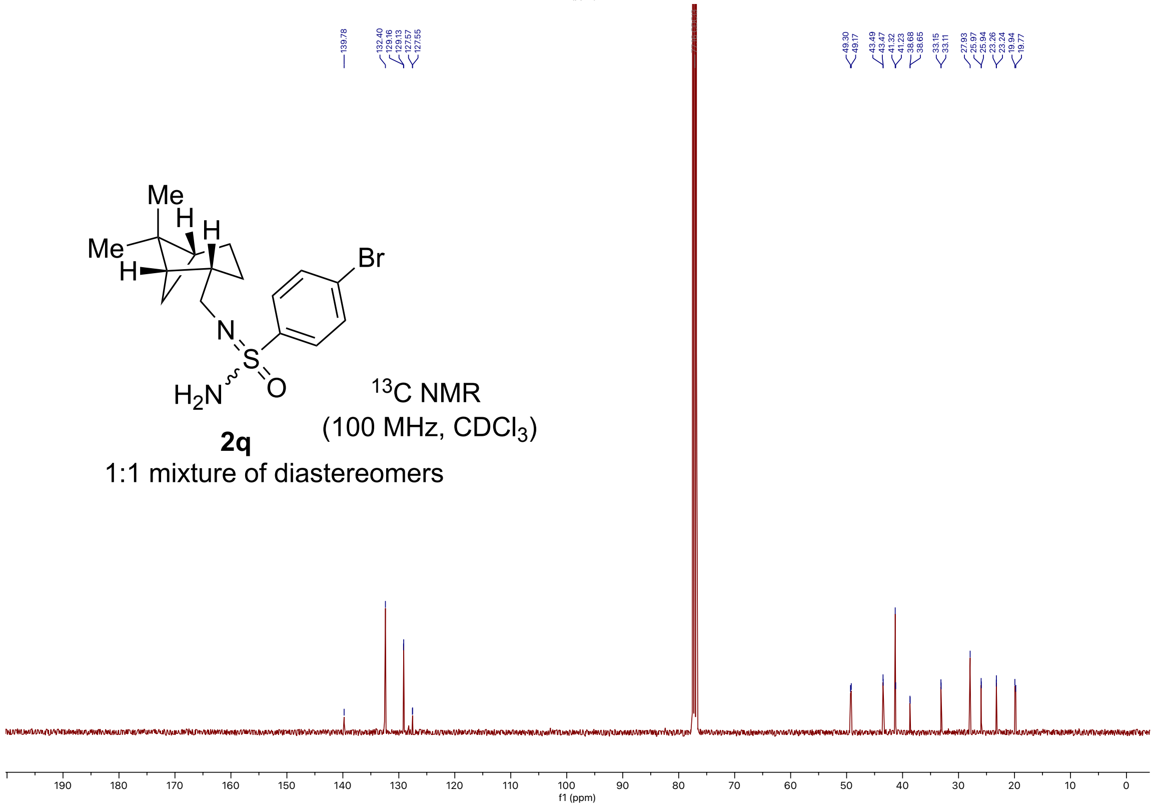
Me
H
H
Me
H
Br
N
S
H_2N
O
2q
1:1 mixture of diastereomers
^{13}C NMR
(100 MHz, $CDCl_3$)
f1 (ppm)

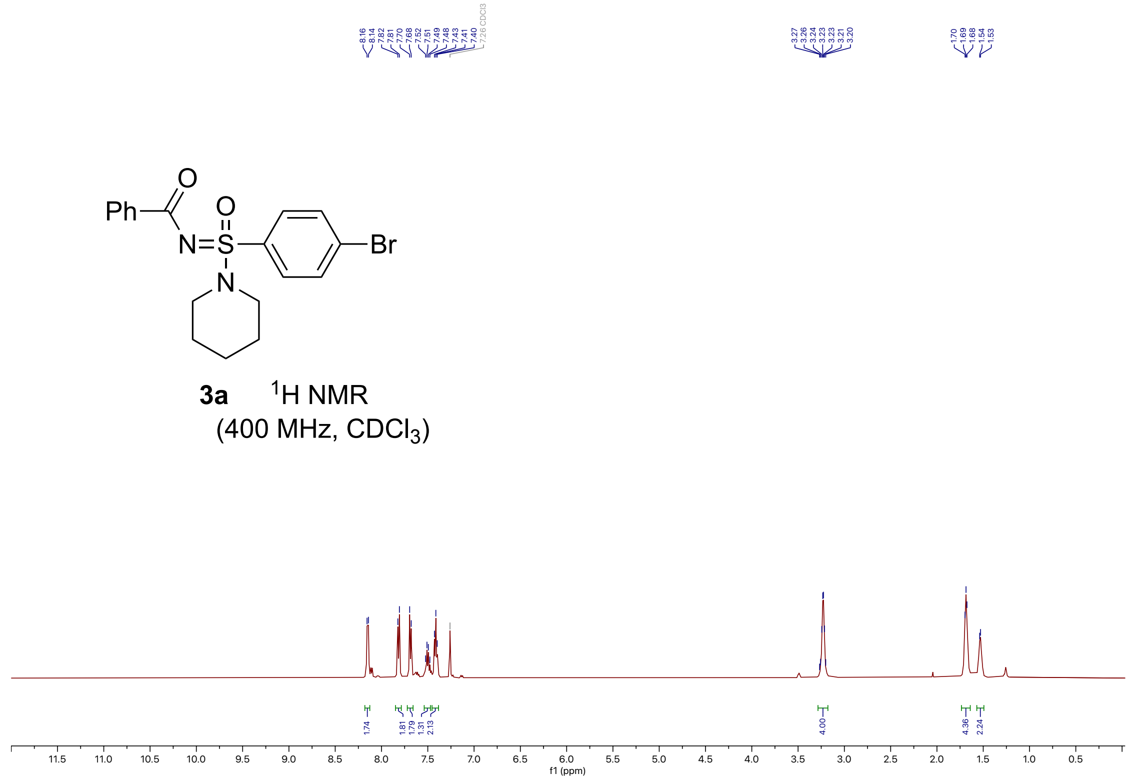
8.16
8.14
7.82
7.81
7.70
7.68
7.52
7.51
7.49
7.48
7.43
7.41
7.40
7.26 CDCl3
3.27
3.26
3.24
3.23
3.23
3.21
3.20
1.70
1.69
1.68
1.54
1.53
Ph
O
O
N
S
N
Br
3a
^{1}H NMR
(400 MHz, $CDCl_3$)
1.74
1.81
1.79
1.31
2.13
4.00
4.36
2.24
11.5
11.0
10.5
10.0
9.5
9.0
8.5
8.0
7.5
7.0
6.5
6.0
5.5
5.0
4.5
4.0
3.5
3.0
2.5
2.0
1.5
1.0
0.5
f1 (ppm)

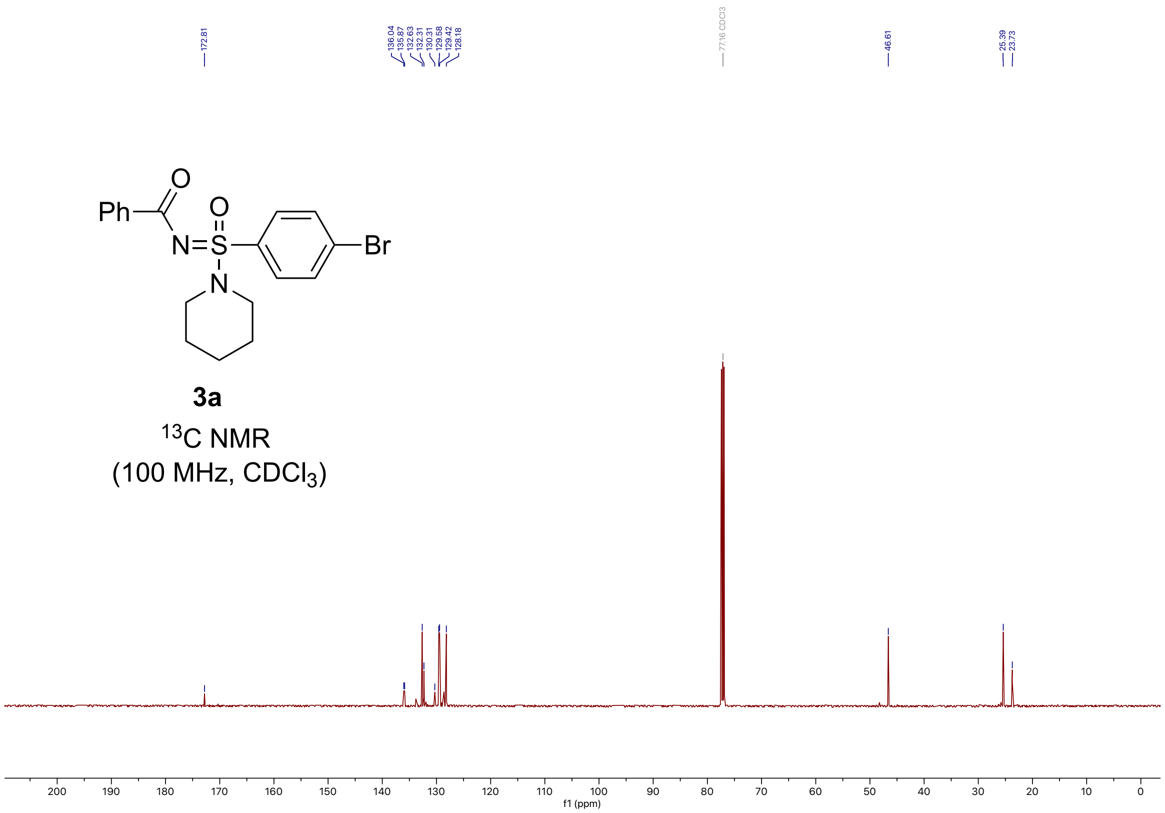
172.81
136.04
135.87
132.63
132.31
130.31
129.58
129.42
128.18
77.16 CDCl3
46.61
25.39
23.73
Ph
O
O
N
S
N
Br
3a
^{13}C NMR
(100 MHz, $CDCl_3$)
200
190
180
170
160
150
140
130
120
110
100
90
80
70
60
50
40
30
20
10
0
f1 (ppm)

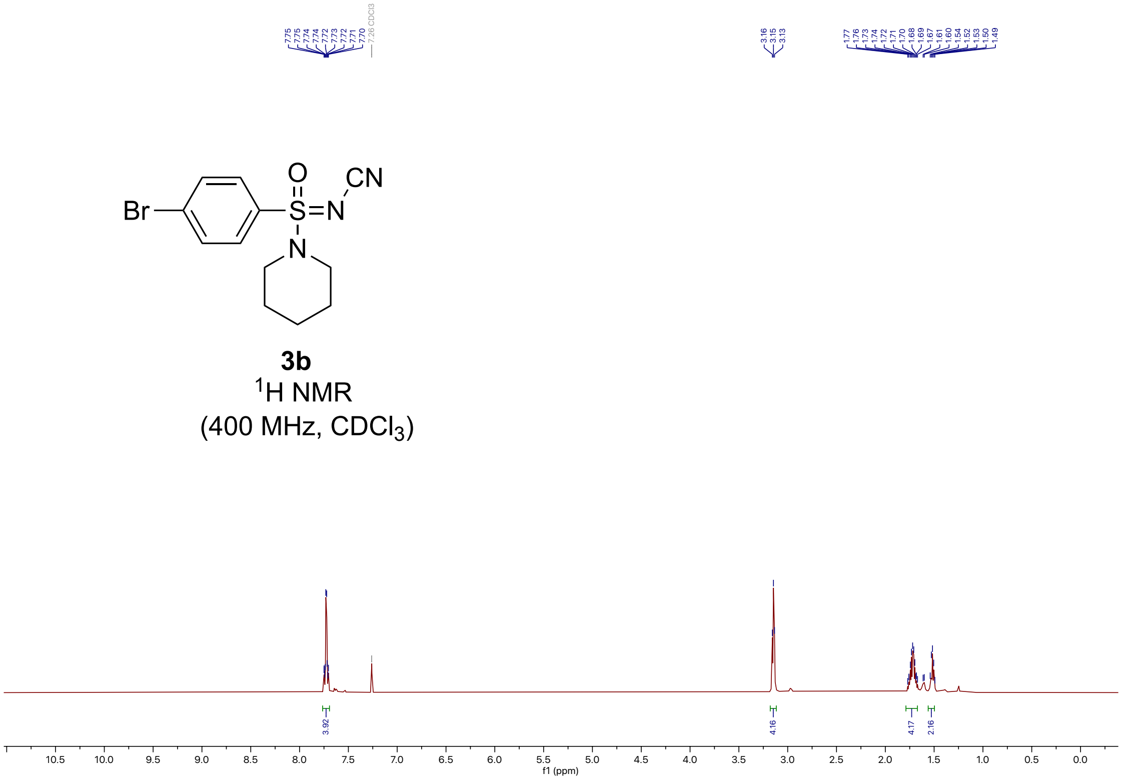

3b
^{1}H NMR
(400 MHz, $CDCl_3$)

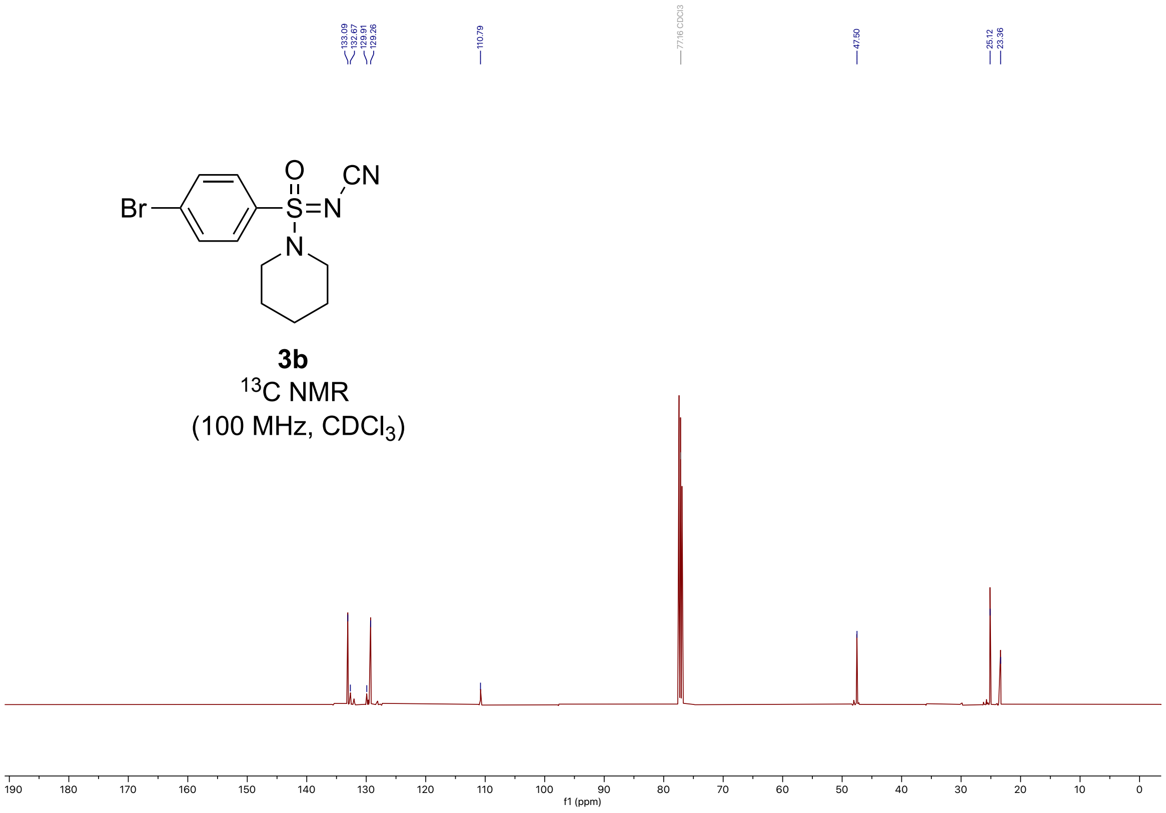

3b
^{13}C NMR
(100 MHz, $CDCl_3$)

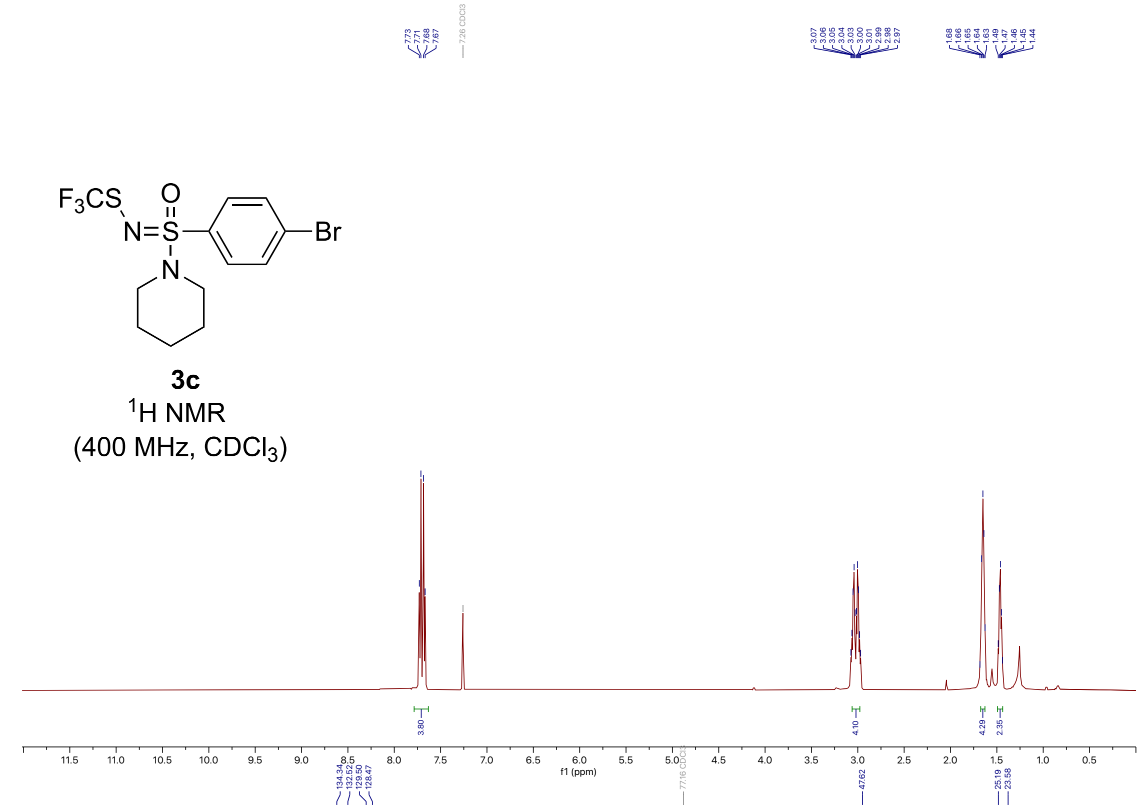

3c
^{1}H NMR
(400 MHz, $CDCl_3$)

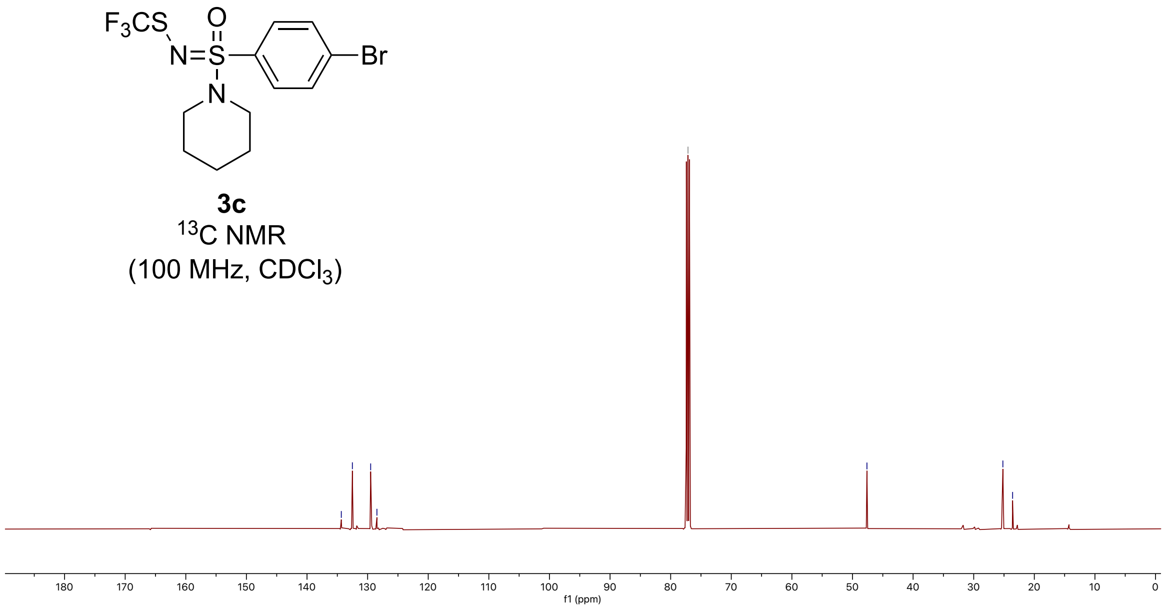

3c
^{13}C NMR
(100 MHz, $CDCl_3$)

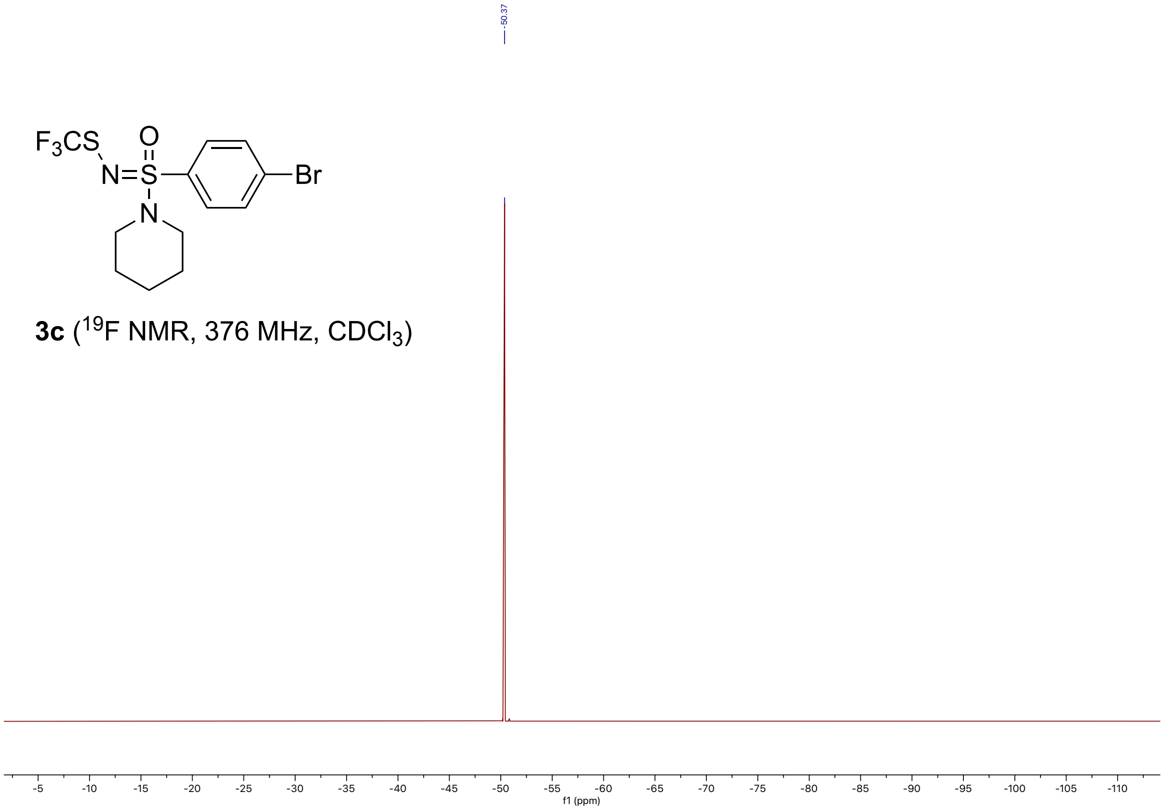

3c (^{19}F NMR, 376 MHz, $CDCl_3$)

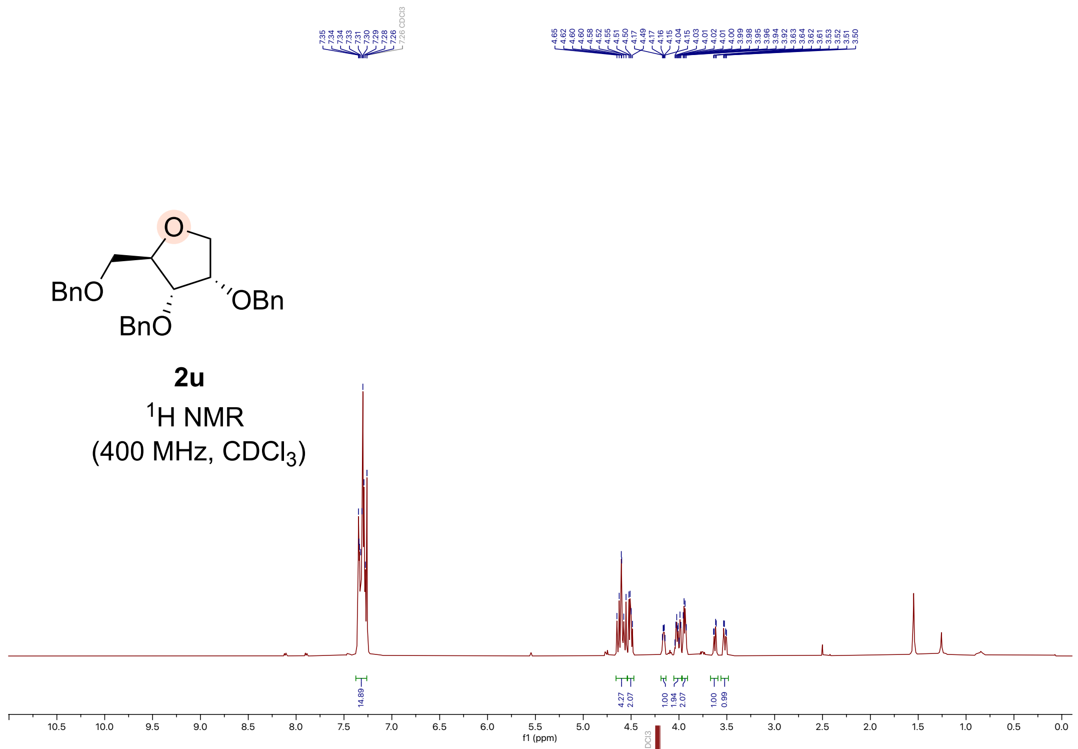
O
BnO
OBn
BnO
2u
1H NMR
(400 MHz, CDCl3)
f1 (ppm)

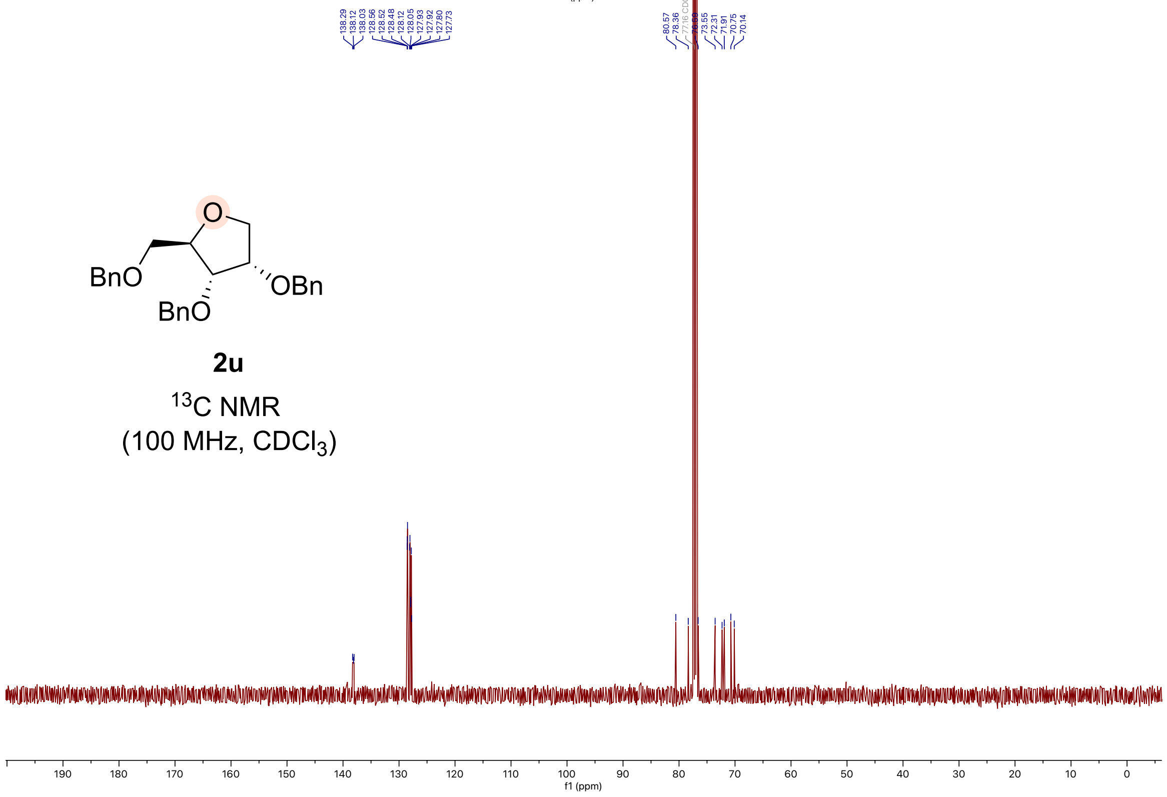
O
BnO
OBn
BnO
2u
13C NMR
(100 MHz, CDCl3)
f1 (ppm)
